# Crystal structure report of the ImmR transcriptional regulator DNA-binding domain of the *Bacillus subtilis* ICE*Bs1* transposon

**DOI:** 10.1038/s41598-022-09237-2

**Published:** 2022-03-28

**Authors:** Rosanna Caliandro, Iñaki de Diego, F. Xavier Gomis-Rüth

**Affiliations:** 1 Proteolysis Laboratory, Department of Structural Biology, Molecular Biology Institute of Barcelona (CSIC), Barcelona Science Park, C/Baldiri Reixac, 15-21, 08028 Barcelona, Catalonia Spain; 2 Present Address: LenioBio GmbH, Erkrather Str. 401, 40231 Düsseldorf, Germany; 3 Present Address: Sample Environment and Characterization Group, European XFEL GmbH, Holzkoppel 4, 22869 Schenefeld, Germany

**Keywords:** Biochemistry, Structural biology

## Abstract

*Bacillus subtilis* is a commensal member of the human oral and gut microbiomes, which can become infectious to immunocompromised patients. It possesses a conjugative transposon, ICE*Bs1*, which includes > 20 genes and can be passed by horizontal gene transfer to other bacteria, including pathogenic *Bacillus anthracis* and *Listeria monocytogenes*. ICE*Bs1* is regulated by the ImmR/ImmA tandem, which are a transcriptional repressor that constitutively blocks transcription and a metallopeptidase that acts as anti-repressor and inactivates ImmR by proteolytic cleavage. We here report the production and purification of 127-residue ImmR from ICE*Bs1* and the crystal structure of its DNA-binding domain. It features a five-helix bundle centred on a helix-turn-helix motif potentially binding the major grove of double-stranded target DNA. ImmR shows structural and mechanistic similarity with the *B. subtilis* SinR repressor, which is engaged in sporulation inhibition.

## Introduction

*Bacillus subtilis* is a Gram-positive bacterium found in the gastrointestinal tract^1^ and the oral cavity of humans^2^, for which it is classified as generally regarded as safe (GRAS)^3^. However, it has been occasionally associated with food poisoning that leads to diarrhea, sickness, and fever in immunocompromised patients^4–6^. Moreover, it shares many features with members of the *Bacillus Cereus Group*^7^, which includes human pathogens such as *Bacillus anthracis* and *B. cereus*.

Conjugative transposons, also referred to as “integrative and conjugative elements” (ICEs), are widespread mobile genetic elements that integrate into the genome of bacteria and provide extra functionalities^8^. They can be excised and shared with other bacteria through conjugation, thus contributing to genome plasticity and the spreading of antibiotic resistance and virulence factors across species^9^. One such ICE from *B. subtilis* is ICE*Bs1*^10^, which can be transferred to *B. anthracis, Bacillus licheniformis*, and *Listeria monocytogenes*^11^. It spans 20 kb and contains over 20 genes transcribed from the P*xis* promoter, which code for the excisionase Xis, the relaxase NicK, the regulator RapI, and the regulatory peptide PhrL, among others^9,12^. Regulation of ICE*Bs1* is exerted by the Int integrase, the ImmR transcriptional repressor, and the ImmA anti-repressor metallopeptidase, which are counter-transcribed from the P*immR* promoter of the transposon^9^. By binding to six sites within the regulatory regions of both promoters, ImmR exerts a repressing function that ensures that a single stable copy of ICE*Bs1* is maintained in the cell in the quiescent state^13^. In contrast, if the global DNA damage response is launched or if potential recipient cells lacking the transposon are nearby, ImmA inactivates ImmR by proteolytic cleavage, which unleashes ICE*Bs1* expression and promotes transposon transfer^11,14^.

ImmR is a 127-residue intracellular protein (UniProt^15^ access code P96631), which was identified as a transcriptional regulator based on sequence similarity with bacteriophage(-like) double-stranded(ds) DNA-binding repressors^10,11,14^. It was predicted to encompass a DNA-binding domain (DBD) with a helix-turn-helix (HTH) motif within its first 61 residues^11,14^. Moreover, the protein was annotated within UniProt as a “HTH-type transcriptional regulator” based on PROSITE-ProRule annotation (PRU00257^16^) but experimental validation is missing. We hereby report the recombinant protein production and purification of ImmR and the crystal structure determination of its DBD.

## Results and discussion

### Structure analysis of the ImmR-DBD

Full-length ImmR of *B. subtilis* was produced by recombinant overexpression in *Escherichia coli* and purified through two chromatography steps. Apparently suitable crystals were routinely obtained but diffraction was consistently restricted to 7–8 Å. Eventually, crystals diffracting to around 2 Å were measured back in 2013 at the ESRF synchrotron beamline ID23-2 (Table 1). However, these crystals suffered from high mosaicity and anisotropy. Moreover, diffraction showed diffuse streaks in several regions of the reciprocal space, potentially arising from planar or linear lattice defects, so that individual diffraction spots were not properly resolved. Given the absence of heavy-atom/ion derivatives or a suitable model for molecular replacement, the project was discontinued until this year, when a predicted model for full-length 127-residue ImmR was obtained with *AlphaFold*^17^. This model divides into a compact high-confidence (∅pLDDT = 96.7%; see^17^ for definition) N-terminal DBD (M^1^–G^63^) and a loose C-terminal domain (K^64^–E^127^) containing two large isolated α-helices (K^64^–K^88^ and E^103^–K^126^), which was predicted with lower overall confidence (∅pLDDT = 74.8%). This result motivated us to reprocess the original diffraction data with up-to-date software.

**Table 1:** Crystallographic data.

**Dataset**	
Beam line (synchrotron)	ID23-2 (ESRF)
Space group/protomers per a.u	I2/2
Cell constants (a, b, and c in Å; β in °)	54.03, 48.34, 64.46, 97.47
Wavelength (Å)	0.87260
Measurements/unique reflections after anisotropy cut-off	26,247/9322
Resolution range (Å) (outermost shell)^a^	43.97–2.10 (2.15–2.10)
Spherical/ellipsoidal completeness (%)^b^	94.0 (71.5)/94.7 (80.8)
R_merge_/R_pim_/CC(^1^/_2_)^c^	0.255 (0.617)/0.186 (0.433)/0.904 (0.591)
< I > /σ(I)^d^/average multiplicity	3.3 (1.7)/2.8 (2.9)
Overall anisotropy B-tensor	19.4, 9.7, 33.41
Resolution range used for refinement (Å)	43.97–2.10
Reflections used (test set)	8867 (454)
Crystallographic R_factor_ (free R_factor_)^c^	0.259 (0.346)
Non-H protein atoms/waters per a.u	1062/142
***Rmsd*** **from target values**	
Bonds (Å)/angles (°)	0.014/1.29
Average B-factor (Å^2^)	12.8
**Protein contacts and geometry analysis**^**e**^	
Ramachandran favoured/allowed/outliers/all analysed	126 (100%)/0/0/126
Bond-length/bond-angle/chirality/planarity outliers	2/0/0/0
Side-chain outliers	8 (7.1%)
All-atom clashes/clashscore^e^	24/10.5
RSRZ outliers^e^/F_o_:F_c_ correlation	5 (3.9%)/0.84
PDB access code	7T8I

*a.u.* asymmetric unit, *rmsd* root-mean square deviation, *RSRZ* real-space R-value Z-score.

^a^Values for data processing in parenthesis refer to the outermost resolution shell if not otherwise indicated.

^b^According to *Mrfana* within *Staraniso*^21^.

^c^For definitions, see^22^.

^d^< I > /σ(I) of unique reflections after merging according to *Mrfana*.

^e^According to the wwPDB Validation Service (https://wwpdb-validation.wwpdb.org/validservice).

**Figure 1:**
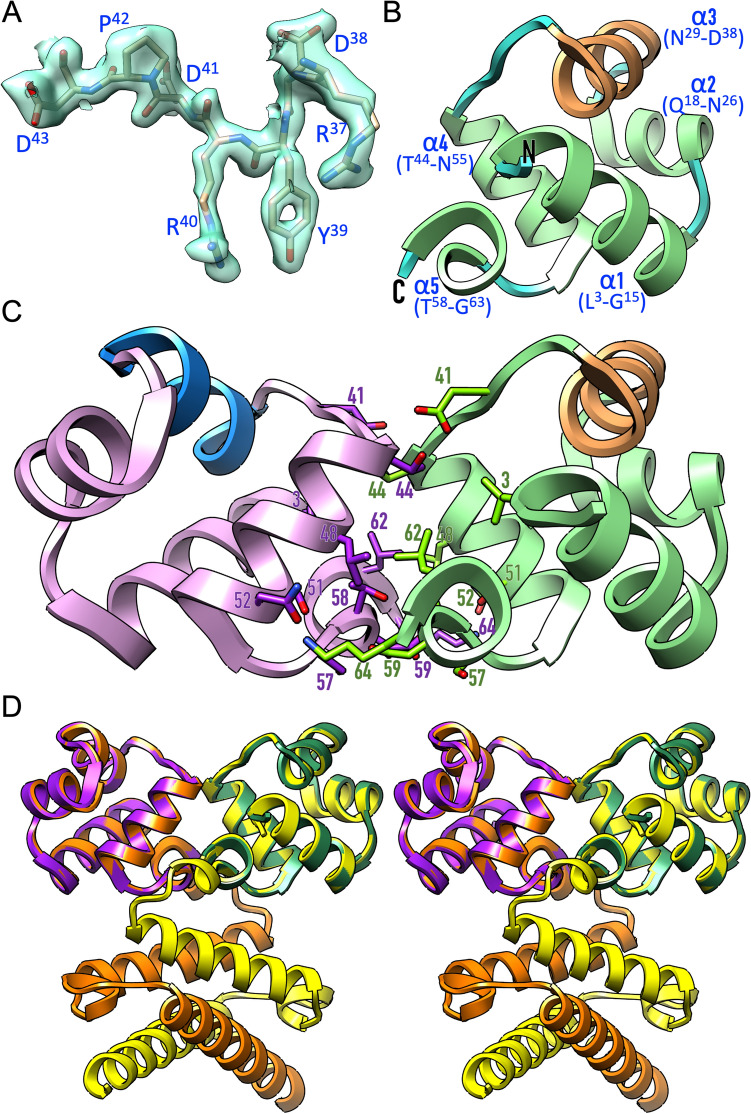
Structure of *B. subtilis* ImmR. **(A)** Representative fragment of the final (2m*F*_obs_ − D*F*_calc_)-type Fourier map displayed at 1 σ above threshold as a semi-transparent turquoise surface, superimposed with segment R^37^–D^43^ of the final refined experimental structure. **(B)** Ribbon-type plot of the ImmR DBD, which consists of five helices (α1–α5). The recognition helix is shown in sandy brown. **(C)** Dimeric arrangement of the ImmR DBD in the crystals. Each protomer is shown in one colour (light green and plum), except the recognition helices (sandy brown and dodger blue). Residues from each protomer contributing to the interface are shown as sticks, with carbons coloured as the respective ribbon, and labelled. **(D)** Superposition in cross-eye stereo of the experimental DBD dimer (chains in purple and green) and the predicted *AlphaFold* dimer of the full-length structure (chains in orange and yellow).

Data processing with *Xds*^18^ and *Dials*^19^ failed in our hands to yield data that would enable crystallographic refinement. Eventually, *iMosflm*^20^ processing, which reportedly deals better with data with large mosaicity and ΔΦ values, followed by anisotropy correction with *Staraniso*^21^, enabled us to get a suitable reflection file for model refinement. This processing yielded comparably high values for the R_merge_ parameter^22^ (see Table 1) but absence of twinning and translational non-crystallographic symmetry. Subsequently, the structure was solved by molecular replacement. While no solution satisfying the packing criterium was obtained for the full-length searching model, two clear solutions were found for the DBD model alone. These solutions showed values for the refined translation functions of 9.9 and 20.4, respectively, and a final log-likelihood gain of 356. After successive rounds of model building and refinement, the final model comprised residues M^1^–K^64^ of molecules A and B, plus respective N-terminal alanines (A^0^) from the purification tag^23^, and 167 solvent molecules. The final values for R_factor_ and R_free_^22^ were comparably high for a dataset to 2.1 Å resolution (Table 1), which we attribute to the above crystal pathologies. This notwithstanding, the final (2m*F*_obs_ − D*F*_calc_)-type Fourier map was of excellent quality for both molecules (Fig. 1A), as were the general model validation parameters (Table 1), so that we are confident that our experimental structure provides a valuable model for the protein. Remarkably, the final Fourier map did not show relevant density beyond K^64^ of either protomer, which would be compatible with the full-length molecule set up for crystallisation being proteolytically processed after this lysine by a trypsin-like contaminant. This hypothesis is supported by calculation of the Matthews-coefficient^24^, which would be 1.4 Å^3^/Da (14% solvent contents) for the full-length protein, which is unlikely. In contrast, the values for the current experimental model (2.8 Å^3^/Da; 57% solvent contents) are in accordance with the literature^25^.

### Description of the ImmR-DBD

The protein is a compact almost spherical pentahelical bundle (α1–α5) cohered by a central hydrophobic core, in which the N- and the C-terminal helices are nearly antiparallel, so that the chain termini are adjacent (Fig. 1B). Helices α2–α4 form a flap that folds back onto the two terminal helices. Overall, the five helices are connected by short linkers of 2-to-5 residues and each helix is approximately perpendicular to the preceding one. Following the nomenclature of HTH_GBB_-DBDs^26^, helices α2 and α3 would correspond to the “positioning helix” and the “recognition helix” of the HTH-motif engaged in double-stranded DNA recognition.

The two protomers in the asymmetric unit (a.u.) are related by a dyad, which gives rise to an interface of 573 Å^2^ (Δ^i^G = − 2.1 kcal/mol; Δ^i^G P-value = 0.424^27^). The interface involves 56 and 49 atoms of 18 residues of molecules A and B, respectively, which overall perform nine hydrogen bonds, as well as symmetric hydrophobic interactions between 11 residues of either molecule. The main participating residues are L^3^, D^41^, T^44^, L^47^, L^48^, S^51^, N^52^, T^58^, D^59^, L^62^, and K^64^ (Fig. 1C), which are provided by helices α4 and α5 plus the linker preceding α4. Finally, the experimental structure is in very good agreement with the predicted dimer (Fig. 1D). Indeed, the 130 residues of the former coincided with the predicted model with a core *rmsd* of 0.43 Å. Moreover, this superposition further revealed that the C-terminal α-helix of the full-length protein would clash with a symmetric DBD mate, which further supports that the crystal only contained the DBD (see “Structure analysis of the ImmR-DBD” section).

### Similar structure

**Figure 2:**
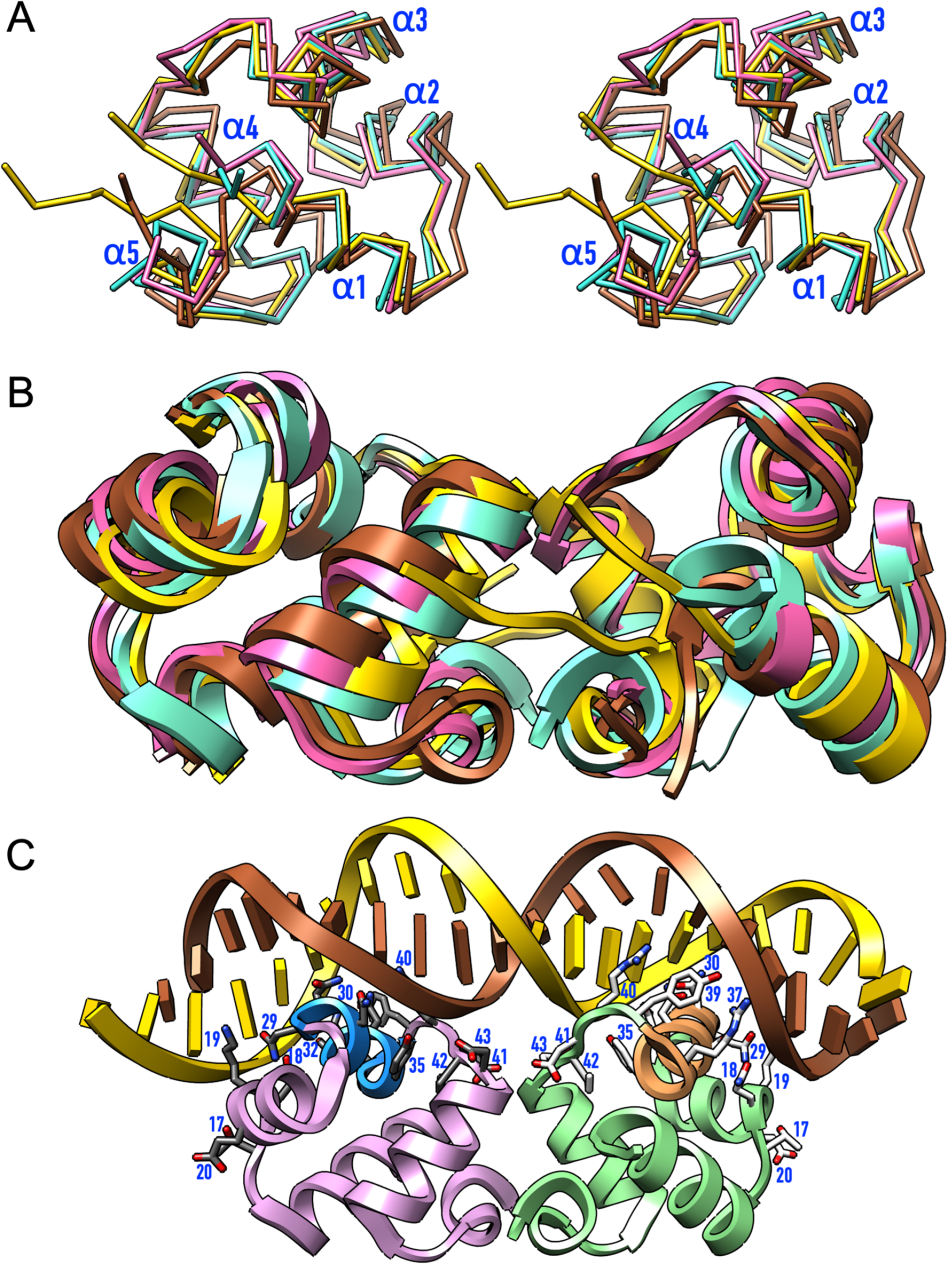
Structural similarities and presumable DNA binding. **(A)** Superposition in cross-eye stereo of the Cα-traces of the monomer of ImmR (aquamarine), which is shown in the orientation of Fig. 1B, onto SinR (hot pink; 62 aligned residues with ImmR show a core *rmsd* of 1.11 Å, 35% sequence identity, and a *Dali* Z-score of 11.4; PDB 1B0N^32^), CylR2 (gold; 56 residues, 0.91 Å, 23%, 11.0; PDB 1UTX^45^), and DdrO (sienna; 63 residues, 1.64 Å, 33%, 10.9; PDB 6JQ1^46^). The five helices are labelled. **(B)** Superposition of the DBD dimers of ImmR (aquamarine), SinR (hot pink; *rmsd* = 1.92 Å for 118 aligned residues; PDB 3ZKC^29^), CylR2 (gold; *rmsd* = 1.45 Å for 119 aligned residues; PDB 1UTX) and DdrO (sienna; *rmsd* = 2.12 Å for 119 aligned residues; PDB 6JQ1). **(C)** Homology model of the protein:dsDNA complex of ImmR based on the structure of the equivalent SinR complex (PDB 3ZKC^29^). The orientation of the protein is the same as in Fig. 1C. Protein residues hypothetically participating in the protein:dsDNA interface are shown as sticks with white and grey carbons for either protomer, respectively, and labelled.

A search with *Dali* identified several members of the “434 Cro family” of HTH-DBDs from bacteria or bacteriophages^26^ as structurally related. Closest similarity was found with 111-residue SinR from *B. subtilis*, followed by the C2 repressor of *Salmonella* bacteriophage P22 (PDB 1ADR^28^), CylR2 of *Enterococcus faecalis*, and DdrO of *Deinococcus geothermalis* (Fig. 2A).

In all structures, the first four helices have a very similar arrangement (Fig. 2A), and significant differences are only found in the respective fifth helices. These have variable length and are shifted along the polypeptide chain in the different structures, which supports that the minimal functional unit for these domains is a four-helix bundle^26^. Moreover, SinR, CylR2, and DdrO evince dimeric crystal structures that are equivalent to that of ImmR (Fig. 2B). In the case of SinR, this dimeric arrangement was functionally validated through the crystal structure of a dsDNA complex^29^ and further suggests that ImmR may oligomerize for the production of DNA-loop structures similar to SinR^30^. We constructed a homology model for the DNA-complex of the ImmR-DBD dimer based on the SinR complex (Fig. 2C). Accordingly, the DNA major groove would be contacted through the recognition helices, and flanking helices α3 and α4 would play a supportive role. Putative residues engaged in binding would encompass T^17^–E^20^, N^29^–N^31^, S^33^–Y^35^, R^37^, and Y^39^–D^43^ of either protomer.

Remarkably, archetypal 434 Cro repressor just spans the pentahelical HTH-DBD^31^ but other family members are C-terminally extended and comprise additional domains. This is the case for SinR, which has two helices engaged in dimerization and binding to other proteins (PDB 1B0N^32^) that are very similar to the *AlphaFold* prediction for ImmR (see “Structure analysis of the ImmR-DBD” section). Given that SinR is currently the closest structural relative of ImmR, both C-terminal regions may have similar functions. Indeed, ImmA inactivates ImmR through cleavage at F^95^–M^96^, which is in the linker between the two predicted helices^14^. This would be consistent with the protein:dsDNA complex falling apart upon cleavage, thus releasing transcriptional repression.

## Materials and methods

### Protein production and purification

The ImmR gene was amplified from *Bacillus subtilis* strain 168 using 5ʹ-CAATCATATGAGCCTAGGCAAACGATTAAAAGAAG-3ʹ and 5ʹ-CAATCTCGAGTCAC TCTTTCTTCTTTAATTCGTCAATG-3ʹ as forward and backward primers, respectively. The PCR product was cloned into the pCri8b vector using *Nde*I and *Xho*I restriction sites, which attaches an N-terminal hexahistidine (His_6_)-tag followed by a tobacco-etch virus (TEV) recognition sequence to the target protein^23^. The plasmid was transformed into *Escherichia coli* BL21 (DE3) cells, which were grown at 20 °C in Luria Bertani medium containing ampicillin (30 μg/mL) and chloramphenicol (34 μg/mL) under agitation (220 rpm) until an OD_600_ of 0.6–1.0 was reached. Expression was then induced by adding 400 μM isopropyl-β-d-thiogalactopyranoside, and the culture was incubated for further 12 h. Cells were harvested by centrifugation at 4000×*g* for 15 min at 4 °C and resuspended in lysis buffer (20 mM Tris–HCl pH 7.5, 5 mM magnesium chloride, 20 mM imidazole, 10 μg/mL DNAse). Cells were lysed in a cell disruptor (Constant Systems, Ltd.), and the lysate was clarified by centrifugation for 1 h at 30,000×*g* at 4 °C. Sodium chloride (1.5 M) was then added to the supernatant and incubated at room temperature for 45 min prior to nickel nitrilotriacetic affinity chromatography purification (NiNTA resin from Invitrogen). The resin had been preequilibrated with buffer A (20 mM Tris–HCl pH 7.5, 1.5 M sodium chloride, 20 mM imidazole), and the protein was eluted with buffer B (20 mM Tris–HCl pH 7.5, 1.5 M sodium chloride, 300 mM imidazole). The protein was then dialysed against buffer A to remove excess of imidazole and incubated with His_6_-tagged TEV protease at a 1:10 molar ratio over night at 4 °C to cleave the N-terminal His_6_-tag. The protein solution was then reapplied to the NiNTA resin pre-equilibrated as before to remove the TEV protease, the cleaved His_6_-tags and non-cleaved N-terminally His_6_-tagged ImmR. The flow through was collected and concentrated to ~ 2 mL using a Vivaspin 20 ultrafiltration device of 5-kDa cut-off (Sartorius). The sample was then run through a Superdex 200 16/60 column (GE Healthcare), which had been attached to an ÄKTA liquid chromatography system (GE Healthcare) and equilibrated with buffer C (20 mM Tris–HCl pH 7.5, 1 M sodium chloride). Fractions corresponding to the protein of interest were collected, and the protein purity and molecular mass (theoretic value 14.8 kDa) were assessed through SDS-PAGE. Protein concentration was determined with a Nanodrop spectrophotometer (Thermo Fisher Scientific) using the theoretical absorption coefficient (ε = 7450 M^−1^ cm^−1^) calculated by *ProtParam* within *Expasy*^33^. Protein identity was confirmed by peptide mass fingerprinting analysis at the Protein Chemistry Service and the Proteomics Facilities of the Centro de Investigaciones Biológicas (Madrid, Spain). Briefly, samples were subjected to 10% SDS-PAGE, and gels were stained for 5 min with freshly prepared Coomasie Blue Stain (0.1% solution in 40% methanol/10% acetic acid) and destained for 15 min in 50% methanol. Gel bands were excised with a clean razor blade and placed in a 1.5-mL Eppendorf tube with 50 μL H_2_O for wet shipment.

### Crystallisation and data collection

Pure protein in 20 mM Tris–HCl pH 7.5, 100 mM sodium chloride was concentrated to 6.5 mg/mL and employed to screen crystallisation conditions applying the sitting-drop vapor diffusion method at the Automated Crystallography Platform (https://www.ibmb.csic.es/en/facilities/automated-crystallographic-platform). Crystallization solutions were prepared with a Freedom EVO robot (Tecan) and pipetted into the reservoir wells of 96 × 2-well MRC crystallization plates (Innovadyne Tech.). Nanodrops consisting of 100 nL of each reservoir solution and protein solution were dispensed by a Cartesian Microsys 4000 XL robot (Genomic Solutions) into the shallow wells of the crystallization plates, which were stored at 4 °C or 20 °C in thermostatic crystal farms (Bruker). Upscaling and optimization were performed by sitting-drop vapor diffusion, using 2 μL protein solution and 1 μL precipitant solution in 24-well Cryschem crystallization plates (Hampton Research).

Suitable crystals of ImmR-DBD were obtained with 18% (w/v) PEG 3350, 10 mM magnesium chloride, 50 mM Tris–HCl pH 8.5 as reservoir solution. Crystals were harvested with cryo-loops (Molecular Dimensions), cryoprotected, flash-vitrified in liquid nitrogen, and stored for data collection. X-ray diffraction data were recorded at 100 K on a 225-mm MARMOSAIC CCD detector (MAR Research) at the ID23-2 beamline^34^ of the ESRF synchrotron (Grenoble, France). Crystals were indexed as space group I2, with two protomers per a.u.. Diffraction data were processed with programs *iMoslfm*^20^ and *Staraniso*^21^, which included the *Mrfana* analysis routine, to obtain structure-factor amplitudes in MTZ-format for the *Phenix*^35^ and *Ccp4*^36^ suites of programs. Data were further assessed with *Xtriage*^37^ within *Phenix* and *Pointless*^38^ within *Ccp4*. Statistics on data collection and processing are provided in Table 1.

### Structure solution and refinement

The structure was solved by molecular replacement with the *Phaser* program^39^ using a homology model for the ImmR-DBD monomer obtained with *AlphaFold*^17^. These calculations yielded two unique solutions at Eulerian angles (in °) α = 116.1, β = 73.5, γ = 25.4 (fractional cell coordinates 0.214, 0.998, 0.333) and α = 296.7, β = 73.6, γ = 25.2 (fractional cell coordinates 0.815, 0.893, 0.331), respectively, which are related by a dyad parallel to cell axis c. The associated values for the translation functions after refinement were 9.9 and 20.4, respectively, and the final log-likelihood gain was 356. The adequately rotated and translated molecules were subjected to the *Autobuild*^40^ protocol within *Phenix*, which yielded a Fourier map of high quality for manual model building with the *Coot* program^41^. The latter alternated with crystallographic refinement using the *Refine* protocol of *Phenix*^35^, which included hydrogens in riding positions and translation/libration/screw-motion plus non-crystallographic symmetry restraints, until completion of the model. Table 1 provides essential statistics on the final refined model, which was validated trough the wwPDB validation service (https://validate-rcsb-1.wwpdb.org/validservice). The coordinates can be retrieved from the Protein Data Bank (www.pdb.org) under access code 7T8I.

### Miscellaneous

Structural relatives were identified through the *Dali*^42^ server (ekhidna2.biocenter.helsinki.fi/dali). Structure superpositions were calculated with *Ssm*^43^ in *Coot*. Figures were prepared using *Chimera*^44^. Protein interfaces and intermolecular interactions were analyzed using *PDBePISA* (www.ebi.ac.uk/pdbe/pisa)^27^ and verified by visual inspection. The interacting surface of a complex was taken as half the sum of the buried surface areas of either molecule. A homology model of the complex between the ImmR-DBD dimer and target dsDNA was obtained by superposing the ImmR dimer onto the SinR dimer within its experimental DNA complex (PDB 3ZKC^29^). This model is provided as Supplementary File 1. The ImmR chain was then slightly readjusted manually with *Coot* and geometry-minimised with the same program to iron out clashes and unfavourable side-chain conformations. The dsDNA part was kept intact.

## Supplementary Information

Supplementary Information.

## Data Availability

The coordinates and structure factors generated during the current study are available from the Protein Data Bank (www.pdb.org) under access code 7T8I.
