# Supplementary material for: Crystal structure report of the ImmR transcriptional regulator DNA-binding domain of the Bacillus subtilis ICEBs1 transposon
Source: Sci Rep. 2022 Mar 28;12:5258. doi: 10.1038/s41598-022-09237-2 (PMC8960796; doi:10.1038/s41598-022-09237-2)
Supplement: Supplementary file 1 — Supplementary Information. [file 41598_2022_9237_MOESM1_ESM.pdf]

ATOM	1	N	GLY	A	0	15.868	4.231	10.648	1.00	47.80	N
ATOM	2	CA	GLY	A	0	16.542	3.488	11.755	1.00	25.62	C
ATOM	3	C	GLY	A	0	15.730	2.327	12.308	1.00	14.60	C
ATOM	4	O	GLY	A	0	16.237	1.221	12.361	1.00	16.84	O
ATOM	10	N	MET	A	1	14.503	2.573	12.780	1.00	17.39	N
ATOM	11	CA	MET	A	1	13.627	1.505	13.249	1.00	15.65	C
ATOM	12	C	MET	A	1	12.331	1.444	12.441	1.00	13.14	C
ATOM	13	O	MET	A	1	11.830	2.459	11.945	1.00	10.41	O
ATOM	14	CB	MET	A	1	13.321	1.657	14.711	1.00	4.73	C
ATOM	15	CG	MET	A	1	12.610	0.467	15.342	1.00	13.08	C
ATOM	16	SD	MET	A	1	13.364	-1.172	15.149	1.00	27.34	S
ATOM	17	CE	MET	A	1	14.643	-1.152	16.376	1.00	27.41	C
ATOM	27	N	SER	A	2	11.777	0.240	12.344	1.00	13.46	N
ATOM	28	CA	SER	A	2	10.708	-0.041	11.399	1.00	21.26	C
ATOM	29	C	SER	A	2	9.869	-1.214	11.899	1.00	5.34	C
ATOM	30	O	SER	A	2	10.217	-1.891	12.856	1.00	5.22	O
ATOM	31	CB	SER	A	2	11.289	-0.330	10.001	1.00	10.49	C
ATOM	32	OG	SER	A	2	12.013	-1.551	9.991	1.00	7.44	O
ATOM	38	N	LEU	A	3	8.720	-1.394	11.249	1.00	5.51	N
ATOM	39	CA	LEU	A	3	7.888	-2.574	11.439	1.00	5.62	C
ATOM	40	C	LEU	A	3	8.634	-3.853	11.054	1.00	5.83	C
ATOM	41	O	LEU	A	3	8.591	-4.855	11.764	1.00	5.81	O
ATOM	42	CB	LEU	A	3	6.640	-2.390	10.593	1.00	10.75	C
ATOM	43	CG	LEU	A	3	5.627	-3.524	10.507	1.00	12.79	C
ATOM	44	CD1	LEU	A	3	4.995	-3.658	11.870	1.00	5.80	C
ATOM	45	CD2	LEU	A	3	4.606	-3.205	9.435	1.00	13.64	C
ATOM	57	N	GLY	A	4	9.314	-3.838	9.915	1.00	13.05	N
ATOM	58	CA	GLY	A	4	10.095	-4.984	9.494	1.00	7.32	C
ATOM	59	C	GLY	A	4	10.991	-5.436	10.637	1.00	6.16	C
ATOM	60	O	GLY	A	4	11.035	-6.611	10.984	1.00	7.13	O
ATOM	64	N	MLZ	A	5	11.694	-4.478	11.230	1.00	7.62	N
ATOM	65	CA	MLZ	A	5	12.725	-4.772	12.249	1.00	5.75	C
ATOM	66	C	MLZ	A	5	12.058	-5.344	13.499	1.00	5.56	C
ATOM	67	O	MLZ	A	5	12.608	-6.340	14.029	1.00	5.64	O
ATOM	68	CB	MLZ	A	5	13.560	-3.525	12.530	1.00	5.56	C
ATOM	69	CG	MLZ	A	5	14.533	-3.130	11.420	1.00	24.90	C
ATOM	70	CD	MLZ	A	5	15.538	-2.060	11.836	1.00	15.89	C
ATOM	71	CE	MLZ	A	5	16.716	-1.890	10.887	1.00	9.73	C
ATOM	72	NZ	MLZ	A	5	17.794	-1.072	11.456	1.00	5.82	N
ATOM	73	CM	MLZ	A	5	18.693	-1.754	12.408	1.00	5.83	C
ATOM	88	N	ARG	A	6	10.888	-4.851	13.899	1.00	5.38	N
ATOM	89	CA	ARG	A	6	10.213	-5.422	15.064	1.00	11.87	C
ATOM	90	C	ARG	A	6	9.595	-6.804	14.783	1.00	6.51	C
ATOM	91	O	ARG	A	6	9.546	-7.640	15.667	1.00	5.47	O
ATOM	92	CB	ARG	A	6	9.140	-4.465	15.602	1.00	5.81	C
ATOM	93	CG	ARG	A	6	9.712	-3.122	16.093	1.00	4.82	C
ATOM	94	CD	ARG	A	6	8.678	-2.260	16.817	1.00	4.67	C
ATOM	95	NE	ARG	A	6	9.133	-0.878	17.009	1.00	10.93	N
ATOM	96	CZ	ARG	A	6	8.958	0.133	16.157	1.00	5.93	C
ATOM	97	NH1	ARG	A	6	8.226	0.012	15.061	1.00	8.27	N
ATOM	98	NH2	ARG	A	6	9.550	1.296	16.401	1.00	7.15	N
ATOM	112	N	LEU	A	7	9.121	-7.038	13.565	1.00	5.75	N
ATOM	113	CA	LEU	A	7	8.638	-8.365	13.208	1.00	6.06	C
ATOM	114	C	LEU	A	7	9.758	-9.372	13.302	1.00	6.24	C
ATOM	115	O	LEU	A	7	9.560	-10.477	13.813	1.00	6.36	O
ATOM	116	CB	LEU	A	7	8.075	-8.371	11.785	1.00	6.39	C
ATOM	117	CG	LEU	A	7	6.677	-7.831	11.558	1.00	6.37	C
ATOM	118	CD1	LEU	A	7	6.532	-7.507	10.109	1.00	11.65	C

ATOM	119	CD2	LEU	A	7	5.650	-8.880	12.031	1.00	6.49	C
ATOM	131	N	LYS	A	8	10.940	-8.997	12.792	1.00	6.31	N
ATOM	132	CA	LYS	A	8	12.097	-9.868	12.885	1.00	6.52	C
ATOM	133	C	LYS	A	8	12.407	-10.187	14.332	1.00	6.30	C
ATOM	134	O	LYS	A	8	12.466	-11.359	14.720	1.00	6.79	O
ATOM	135	CB	LYS	A	8	13.287	-9.219	12.181	1.00	6.62	C
ATOM	136	CG	LYS	A	8	14.509	-10.031	12.213	1.00	6.89	C
ATOM	137	CD	LYS	A	8	15.573	-9.485	11.298	1.00	7.09	C
ATOM	138	CE	LYS	A	8	16.771	-10.426	11.298	1.00	19.22	C
ATOM	139	NZ	LYS	A	8	16.447	-11.842	10.837	1.00	27.01	N
ATOM	153	N	GLU	A	9	12.550	-9.148	15.158	1.00	5.94	N
ATOM	154	CA	GLU	A	9	12.900	-9.366	16.560	1.00	5.77	C
ATOM	155	C	GLU	A	9	11.840	-10.175	17.285	1.00	15.99	C
ATOM	156	O	GLU	A	9	12.164	-11.058	18.089	1.00	5.84	O
ATOM	157	CB	GLU	A	9	13.071	-8.022	17.275	1.00	12.17	C
ATOM	158	CG	GLU	A	9	13.161	-8.144	18.800	1.00	15.78	C
ATOM	159	CD	GLU	A	9	13.829	-6.946	19.446	1.00	27.46	C
ATOM	160	OE1	GLU	A	9	14.106	-5.935	18.738	1.00	17.35	O
ATOM	161	OE2	GLU	A	9	14.106	-7.033	20.663	1.00	39.41	O
ATOM	168	N	ALA	A	10	10.564	-9.859	17.052	1.00	5.67	N
ATOM	169	CA	ALA	A	10	9.494	-10.542	17.759	1.00	9.93	C
ATOM	170	C	ALA	A	10	9.477	-12.016	17.384	1.00	6.03	C
ATOM	171	O	ALA	A	10	9.308	-12.877	18.252	1.00	15.14	O
ATOM	172	CB	ALA	A	10	8.163	-9.822	17.469	1.00	5.56	C
ATOM	178	N	ARG	A	11	9.716	-12.323	16.098	1.00	12.42	N
ATOM	179	CA	ARG	A	11	9.809	-13.716	15.639	1.00	13.50	C
ATOM	180	C	ARG	A	11	10.976	-14.460	16.274	1.00	7.91	C
ATOM	181	O	ARG	A	11	10.871	-15.644	16.612	1.00	7.12	O
ATOM	182	CB	ARG	A	11	9.972	-13.749	14.127	1.00	9.16	C
ATOM	183	CG	ARG	A	11	10.303	-15.128	13.606	1.00	9.66	C
ATOM	184	CD	ARG	A	11	10.441	-15.188	12.069	1.00	8.00	C
ATOM	185	NE	ARG	A	11	11.355	-14.217	11.495	1.00	9.88	N
ATOM	186	CZ	ARG	A	11	12.668	-14.344	11.540	1.00	8.03	C
ATOM	187	NH1	ARG	A	11	13.232	-15.346	12.185	1.00	11.72	N
ATOM	188	NH2	ARG	A	11	13.430	-13.461	10.904	1.00	8.44	N
ATOM	202	N	GLN	A	12	12.118	-13.800	16.368	1.00	6.75	N
ATOM	203	CA	GLN	A	12	13.251	-14.409	17.045	1.00	14.93	C
ATOM	204	C	GLN	A	12	12.945	-14.627	18.520	1.00	6.68	C
ATOM	205	O	GLN	A	12	13.204	-15.706	19.055	1.00	6.91	O
ATOM	206	CB	GLN	A	12	14.495	-13.554	16.820	1.00	6.82	C
ATOM	207	CG	GLN	A	12	14.895	-13.619	15.332	1.00	7.15	C
ATOM	208	CD	GLN	A	12	16.155	-12.828	14.943	1.00	15.35	C
ATOM	209	OE1	GLN	A	12	16.653	-11.950	15.662	1.00	10.42	O
ATOM	210	NE2	GLN	A	12	16.667	-13.153	13.780	1.00	8.31	N
ATOM	219	N	LYS	A	13	12.294	-13.670	19.169	1.00	6.29	N
ATOM	220	CA	LYS	A	13	11.929	-13.884	20.560	1.00	9.00	C
ATOM	221	C	LYS	A	13	11.003	-15.087	20.705	1.00	12.30	C
ATOM	222	O	LYS	A	13	11.041	-15.773	21.724	1.00	7.22	O
ATOM	223	CB	LYS	A	13	11.305	-12.610	21.149	1.00	12.35	C
ATOM	224	CG	LYS	A	13	12.368	-11.630	21.619	1.00	9.44	C
ATOM	225	CD	LYS	A	13	11.840	-10.390	22.292	1.00	29.73	C
ATOM	226	CE	LYS	A	13	12.967	-9.344	22.369	1.00	31.58	C
ATOM	227	NZ	LYS	A	13	12.544	-7.992	22.843	1.00	24.45	N
ATOM	241	N	ALA	A	14	10.128	-15.327	19.727	1.00	9.51	N
ATOM	242	CA	ALA	A	14	9.212	-16.453	19.843	1.00	15.92	C
ATOM	243	C	ALA	A	14	9.867	-17.804	19.555	1.00	7.19	C
ATOM	244	O	ALA	A	14	9.284	-18.844	19.891	1.00	9.71	O
ATOM	245	CB	ALA	A	14	8.010	-16.234	18.925	1.00	12.97	C

ATOM	251	N	GLY	A	15	11.041	-17.808	18.921	1.00	9.37	N
ATOM	252	CA	GLY	A	15	11.775	-19.002	18.607	1.00	7.83	C
ATOM	253	C	GLY	A	15	11.687	-19.455	17.175	1.00	8.23	C
ATOM	254	O	GLY	A	15	12.443	-20.361	16.796	1.00	10.93	O
ATOM	258	N	TYR	A	16	10.830	-18.841	16.352	1.00	8.13	N
ATOM	259	CA	TYR	A	16	10.621	-19.315	14.983	1.00	8.73	C
ATOM	260	C	TYR	A	16	11.712	-18.839	14.027	1.00	8.73	C
ATOM	261	O	TYR	A	16	12.128	-17.685	14.054	1.00	8.37	O
ATOM	262	CB	TYR	A	16	9.272	-18.843	14.430	1.00	14.68	C
ATOM	263	CG	TYR	A	16	8.042	-19.292	15.170	1.00	8.41	C
ATOM	264	CD1	TYR	A	16	7.444	-20.505	14.878	1.00	9.27	C
ATOM	265	CD2	TYR	A	16	7.422	-18.461	16.100	1.00	7.92	C
ATOM	266	CE1	TYR	A	16	6.310	-20.911	15.537	1.00	8.84	C
ATOM	267	CE2	TYR	A	16	6.300	-18.857	16.754	1.00	7.90	C
ATOM	268	CZ	TYR	A	16	5.750	-20.095	16.481	1.00	8.35	C
ATOM	269	OH	TYR	A	16	4.599	-20.478	17.130	1.00	17.33	O
ATOM	279	N	THR	A	17	12.122	-19.732	13.132	1.00	9.31	N
ATOM	280	CA	THR	A	17	12.849	-19.325	11.941	1.00	13.39	C
ATOM	281	C	THR	A	17	11.906	-18.670	10.927	1.00	11.73	C
ATOM	282	O	THR	A	17	10.687	-18.749	11.027	1.00	9.45	O
ATOM	283	CB	THR	A	17	13.528	-20.520	11.277	1.00	11.04	C
ATOM	284	OG1	THR	A	17	12.542	-21.403	10.733	1.00	10.69	O
ATOM	285	CG2	THR	A	17	14.355	-21.261	12.273	1.00	10.36	C
ATOM	293	N	GLN	A	18	12.488	-18.040	9.918	1.00	9.68	N
ATOM	294	CA	GLN	A	18	11.662	-17.335	8.927	1.00	14.58	C
ATOM	295	C	GLN	A	18	10.655	-18.298	8.241	1.00	10.31	C
ATOM	296	O	GLN	A	18	9.464	-17.988	8.101	1.00	10.09	O
ATOM	297	CB	GLN	A	18	12.564	-16.638	7.884	1.00	16.43	C
ATOM	298	CG	GLN	A	18	11.958	-15.394	7.243	1.00	9.65	C
ATOM	299	CD	GLN	A	18	12.917	-14.687	6.315	1.00	9.82	C
ATOM	300	OE1	GLN	A	18	13.177	-15.152	5.220	1.00	14.63	O
ATOM	301	NE2	GLN	A	18	13.394	-13.530	6.724	1.00	19.85	N
ATOM	310	N	MLZ	A	19	11.066	-19.382	7.735	1.00	14.41	N
ATOM	311	CA	MLZ	A	19	10.304	-20.424	6.984	1.00	13.10	C
ATOM	312	C	MLZ	A	19	9.362	-21.131	7.959	1.00	13.30	C
ATOM	313	O	MLZ	A	19	8.237	-21.443	7.554	1.00	11.53	O
ATOM	314	CB	MLZ	A	19	11.209	-21.444	6.275	1.00	23.52	C
ATOM	315	CG	MLZ	A	19	10.714	-21.984	4.942	1.00	31.88	C
ATOM	316	CD	MLZ	A	19	11.710	-21.761	3.810	1.00	31.81	C
ATOM	317	CE	MLZ	A	19	11.071	-21.737	2.431	1.00	13.12	C
ATOM	318	NZ	MLZ	A	19	12.031	-22.055	1.370	1.00	10.98	N
ATOM	319	CM	MLZ	A	19	11.759	-23.376	0.760	1.00	29.77	C
ATOM	334	N	GLU	A	20	9.715	-21.393	9.277	1.00	10.95	N
ATOM	335	CA	GLU	A	20	8.832	-22.025	10.217	1.00	10.84	C
ATOM	336	C	GLU	A	20	7.618	-21.111	10.395	1.00	10.38	C
ATOM	337	O	GLU	A	20	6.487	-21.553	10.300	1.00	10.90	O
ATOM	338	CB	GLU	A	20	9.556	-22.294	11.542	1.00	10.55	C
ATOM	339	CG	GLU	A	20	10.407	-23.539	11.567	1.00	14.09	C
ATOM	340	CD	GLU	A	20	11.220	-23.681	12.867	1.00	10.83	C
ATOM	341	OE1	GLU	A	20	11.297	-22.699	13.631	1.00	10.22	O
ATOM	342	OE2	GLU	A	20	11.763	-24.781	13.102	1.00	11.28	O
ATOM	349	N	ALA	A	21	7.872	-19.812	10.599	1.00	9.84	N
ATOM	350	CA	ALA	A	21	6.786	-18.848	10.795	1.00	9.43	C
ATOM	351	C	ALA	A	21	5.946	-18.680	9.536	1.00	17.19	C
ATOM	352	O	ALA	A	21	4.700	-18.621	9.593	1.00	14.94	O
ATOM	353	CB	ALA	A	21	7.370	-17.511	11.202	1.00	8.87	C
ATOM	359	N	ALA	A	22	6.615	-18.592	8.390	1.00	10.14	N
ATOM	360	CA	ALA	A	22	5.890	-18.474	7.138	1.00	24.50	C

ATOM	361	C	ALA	A	22	4.953	-19.660	6.970	1.00	18.06	C
ATOM	362	O	ALA	A	22	3.786	-19.495	6.595	1.00	18.36	O
ATOM	363	CB	ALA	A	22	6.882	-18.379	5.974	1.00	10.97	C
ATOM	369	N	GLU	A	23	5.441	-20.864	7.257	1.00	11.40	N
ATOM	370	CA	GLU	A	23	4.615	-22.054	7.076	1.00	16.26	C
ATOM	371	C	GLU	A	23	3.454	-22.079	8.059	1.00	11.61	C
ATOM	372	O	GLU	A	23	2.333	-22.433	7.691	1.00	33.87	O
ATOM	373	CB	GLU	A	23	5.479	-23.311	7.175	1.00	27.24	C
ATOM	374	CG	GLU	A	23	6.402	-23.477	5.957	1.00	22.52	C
ATOM	375	CD	GLU	A	23	7.415	-24.611	6.090	1.00	41.21	C
ATOM	376	OE1	GLU	A	23	7.213	-25.526	6.926	1.00	31.71	O
ATOM	377	OE2	GLU	A	23	8.420	-24.582	5.348	1.00	46.84	O
ATOM	384	N	LYS	A	24	3.693	-21.704	9.315	1.00	23.58	N
ATOM	385	CA	LYS	A	24	2.583	-21.626	10.253	1.00	31.91	C
ATOM	386	C	LYS	A	24	1.492	-20.676	9.759	1.00	34.84	C
ATOM	387	O	LYS	A	24	0.303	-20.921	9.990	1.00	23.02	O
ATOM	388	CB	LYS	A	24	3.097	-21.194	11.615	1.00	24.53	C
ATOM	389	CG	LYS	A	24	3.642	-22.328	12.436	1.00	25.14	C
ATOM	390	CD	LYS	A	24	3.242	-22.196	13.879	1.00	37.53	C
ATOM	391	CE	LYS	A	24	1.749	-22.321	14.059	1.00	39.65	C
ATOM	392	NZ	LYS	A	24	1.367	-23.652	14.573	1.00	17.97	N
ATOM	406	N	LEU	A	25	1.870	-19.574	9.112	1.00	28.11	N
ATOM	407	CA	LEU	A	25	0.889	-18.600	8.656	1.00	17.81	C
ATOM	408	C	LEU	A	25	0.472	-18.798	7.210	1.00	20.91	C
ATOM	409	O	LEU	A	25	-0.341	-18.026	6.697	1.00	25.72	O
ATOM	410	CB	LEU	A	25	1.437	-17.197	8.868	1.00	9.74	C
ATOM	411	CG	LEU	A	25	1.472	-16.875	10.373	1.00	22.17	C
ATOM	412	CD1	LEU	A	25	1.902	-15.440	10.644	1.00	20.35	C
ATOM	413	CD2	LEU	A	25	0.106	-17.148	11.069	1.00	14.97	C
ATOM	425	N	ASN	A	26	0.977	-19.835	6.559	1.00	24.21	N
ATOM	426	CA	ASN	A	26	0.652	-20.136	5.163	1.00	29.16	C
ATOM	427	C	ASN	A	26	0.843	-18.907	4.284	1.00	30.90	C
ATOM	428	O	ASN	A	26	0.001	-18.569	3.451	1.00	36.70	O
ATOM	429	CB	ASN	A	26	-0.761	-20.702	4.995	1.00	33.99	C
ATOM	430	CG	ASN	A	26	-0.954	-21.363	3.642	1.00	48.17	C
ATOM	431	OD1	ASN	A	26	-0.006	-21.913	3.069	1.00	41.55	O
ATOM	432	ND2	ASN	A	26	-2.180	-21.321	3.126	1.00	48.90	N
ATOM	439	N	ILE	A	27	1.949	-18.198	4.519	1.00	13.98	N
ATOM	440	C	ILE	A	27	3.634	-17.420	2.968	1.00	23.10	C
ATOM	441	O	ILE	A	27	4.364	-18.274	3.522	1.00	34.84	O
ATOM	458	CA	ILE	A	27	2.306	-17.049	3.637	1.00	24.38	C
ATOM	459	CB	ILE	A	27	2.402	-15.721	4.418	1.00	26.60	C
ATOM	460	CG1	ILE	A	27	3.309	-15.832	5.647	1.00	19.82	C
ATOM	461	CG2	ILE	A	27	1.019	-15.201	4.784	1.00	29.01	C
ATOM	462	CD1	ILE	A	27	3.995	-14.539	6.014	1.00	14.57	C
ATOM	474	N	GLY	A	28	3.936	-16.816	1.817	1.00	30.50	N
ATOM	475	CA	GLY	A	28	5.226	-17.086	1.155	1.00	33.27	C
ATOM	476	C	GLY	A	28	6.399	-16.770	2.066	1.00	34.74	C
ATOM	477	O	GLY	A	28	6.322	-15.762	2.797	1.00	21.66	O
ATOM	481	N	ASN	A	29	7.332	-17.716	2.255	1.00	30.95	N
ATOM	482	CA	ASN	A	29	8.554	-17.382	3.043	1.00	27.30	C
ATOM	483	C	ASN	A	29	9.056	-16.104	2.398	1.00	23.75	C
ATOM	484	O	ASN	A	29	9.553	-15.218	3.125	1.00	11.80	O
ATOM	485	CB	ASN	A	29	9.580	-18.514	3.033	1.00	29.58	C
ATOM	486	CG	ASN	A	29	10.989	-18.055	3.343	1.00	23.30	C
ATOM	487	OD1	ASN	A	29	11.820	-17.944	2.446	1.00	38.74	O
ATOM	488	ND2	ASN	A	29	11.267	-17.787	4.608	1.00	35.34	N
ATOM	495	N	ASN	A	30	8.925	-16.044	1.077	1.00	30.09	N

ATOM	496	CA	ASN	A	30	9.218	-14.826	0.305	1.00	12.44	C
ATOM	497	C	ASN	A	30	8.742	-13.576	1.025	1.00	19.81	C
ATOM	498	O	ASN	A	30	9.532	-12.619	1.126	1.00	24.04	O
ATOM	499	CB	ASN	A	30	8.279	-14.899	-0.885	1.00	15.84	C
ATOM	500	CG	ASN	A	30	8.481	-13.786	-1.872	1.00	39.18	C
ATOM	501	OD1	ASN	A	30	7.842	-12.736	-1.811	1.00	34.90	O
ATOM	502	ND2	ASN	A	30	9.357	-14.054	-2.812	1.00	34.31	N
ATOM	509	N	ASN	A	31	7.458	-13.577	1.409	1.00	16.46	N
ATOM	510	CA	ASN	A	31	6.818	-12.388	2.029	1.00	13.20	C
ATOM	511	C	ASN	A	31	7.296	-12.184	3.464	1.00	10.27	C
ATOM	512	O	ASN	A	31	7.568	-11.027	3.797	1.00	9.83	O
ATOM	513	CB	ASN	A	31	5.293	-12.507	2.001	1.00	31.75	C
ATOM	514	CG	ASN	A	31	4.738	-12.506	0.595	1.00	38.73	C
ATOM	515	OD1	ASN	A	31	4.984	-11.576	-0.170	1.00	39.56	O
ATOM	516	ND2	ASN	A	31	3.990	-13.540	0.249	1.00	53.17	N
ATOM	523	N	LEU	A	32	7.369	-13.238	4.287	1.00	10.16	N
ATOM	524	CA	LEU	A	32	7.918	-13.001	5.639	1.00	9.50	C
ATOM	525	C	LEU	A	32	9.229	-12.250	5.407	1.00	9.30	C
ATOM	526	O	LEU	A	32	9.479	-11.312	6.147	1.00	8.76	O
ATOM	527	CB	LEU	A	32	8.122	-14.306	6.412	1.00	11.84	C
ATOM	528	CG	LEU	A	32	7.940	-14.184	7.928	1.00	8.93	C
ATOM	529	CD1	LEU	A	32	7.452	-12.801	8.335	1.00	8.46	C
ATOM	530	CD2	LEU	A	32	6.960	-15.217	8.432	1.00	9.10	C
ATOM	542	N	SER	A	33	9.947	-12.537	4.315	1.00	9.76	N
ATOM	543	CA	SER	A	33	11.219	-11.836	4.195	1.00	9.65	C
ATOM	544	C	SER	A	33	11.029	-10.418	3.681	1.00	9.45	C
ATOM	545	O	SER	A	33	11.649	-9.497	4.194	1.00	9.03	O
ATOM	546	CB	SER	A	33	12.160	-12.595	3.276	1.00	10.38	C
ATOM	547	OG	SER	A	33	13.351	-11.832	3.069	1.00	25.10	O
ATOM	553	N	ASN	A	34	10.178	-10.231	2.664	1.00	9.79	N
ATOM	554	CA	ASN	A	34	9.881	-8.893	2.169	1.00	11.31	C
ATOM	555	C	ASN	A	34	9.289	-8.011	3.261	1.00	8.99	C
ATOM	556	O	ASN	A	34	9.688	-6.847	3.403	1.00	8.67	O
ATOM	557	CB	ASN	A	34	8.945	-8.984	0.950	1.00	10.18	C
ATOM	558	CG	ASN	A	34	9.681	-9.381	-0.305	1.00	10.85	C
ATOM	559	OD1	ASN	A	34	10.895	-9.296	-0.363	1.00	10.87	O
ATOM	560	ND2	ASN	A	34	8.954	-9.828	-1.310	1.00	11.44	N
ATOM	567	N	TYR	A	35	8.409	-8.576	4.091	1.00	8.80	N
ATOM	568	CA	TYR	A	35	7.810	-7.804	5.213	1.00	8.22	C
ATOM	569	C	TYR	A	35	8.894	-7.411	6.222	1.00	10.55	C
ATOM	570	O	TYR	A	35	8.919	-6.231	6.620	1.00	10.23	O
ATOM	571	CB	TYR	A	35	6.706	-8.602	5.912	1.00	8.12	C
ATOM	572	CG	TYR	A	35	5.540	-8.998	5.042	1.00	8.55	C
ATOM	573	CD1	TYR	A	35	5.240	-8.307	3.879	1.00	8.86	C
ATOM	574	CD2	TYR	A	35	4.729	-10.066	5.388	1.00	15.48	C
ATOM	575	CE1	TYR	A	35	4.171	-8.673	3.077	1.00	9.30	C
ATOM	576	CE2	TYR	A	35	3.654	-10.442	4.600	1.00	9.12	C
ATOM	577	CZ	TYR	A	35	3.373	-9.742	3.441	1.00	9.44	C
ATOM	578	OH	TYR	A	35	2.314	-10.109	2.662	1.00	10.66	O
ATOM	588	N	GLU	A	36	9.757	-8.355	6.620	1.00	8.37	N
ATOM	589	CA	GLU	A	36	10.757	-8.066	7.637	1.00	10.93	C
ATOM	590	C	GLU	A	36	11.758	-7.017	7.130	1.00	15.43	C
ATOM	591	O	GLU	A	36	12.270	-6.220	7.915	1.00	12.07	O
ATOM	592	CB	GLU	A	36	11.462	-9.349	8.064	1.00	7.66	C
ATOM	593	CG	GLU	A	36	10.541	-10.383	8.735	1.00	10.34	C
ATOM	594	CD	GLU	A	36	11.261	-11.623	9.252	1.00	7.85	C
ATOM	595	OE1	GLU	A	36	12.468	-11.808	8.997	1.00	8.04	O
ATOM	596	OE2	GLU	A	36	10.621	-12.406	9.967	1.00	7.82	O

ATOM	603	N	ARG	A	37	12.117	-7.094	5.845	1.00	9.84	N
ATOM	604	CA	ARG	A	37	13.159	-6.177	5.303	1.00	13.24	C
ATOM	605	C	ARG	A	37	12.514	-4.876	4.818	1.00	8.21	C
ATOM	606	O	ARG	A	37	13.216	-4.112	4.120	1.00	9.10	O
ATOM	607	CB	ARG	A	37	13.930	-6.865	4.174	1.00	15.94	C
ATOM	608	CG	ARG	A	37	14.982	-7.852	4.654	1.00	25.84	C
ATOM	609	CD	ARG	A	37	15.122	-9.005	3.684	1.00	33.90	C
ATOM	610	NE	ARG	A	37	16.159	-8.780	2.688	1.00	24.19	N
ATOM	611	CZ	ARG	A	37	16.195	-9.350	1.489	1.00	24.94	C
ATOM	612	NH1	ARG	A	37	15.243	-10.191	1.120	1.00	18.61	N
ATOM	613	NH2	ARG	A	37	17.186	-9.076	0.661	1.00	16.83	N
ATOM	627	N	ASP	A	38	11.237	-4.650	5.149	1.00	7.61	N
ATOM	628	CA	ASP	A	38	10.510	-3.406	4.763	1.00	7.52	C
ATOM	629	C	ASP	A	38	10.531	-3.205	3.244	1.00	15.67	C
ATOM	630	O	ASP	A	38	10.444	-2.036	2.813	1.00	13.05	O
ATOM	631	CB	ASP	A	38	11.037	-2.188	5.526	1.00	7.12	C
ATOM	632	CG	ASP	A	38	10.958	-2.389	7.023	1.00	6.66	C
ATOM	633	OD1	ASP	A	38	9.887	-2.102	7.591	1.00	12.69	O
ATOM	634	OD2	ASP	A	38	11.959	-2.857	7.599	1.00	6.50	O
ATOM	639	N	TYR	A	39	10.633	-4.287	2.466	1.00	8.49	N
ATOM	640	CA	TYR	A	39	10.530	-4.159	0.993	1.00	15.60	C
ATOM	641	C	TYR	A	39	9.153	-3.571	0.705	1.00	9.18	C
ATOM	642	O	TYR	A	39	9.060	-2.617	-0.094	1.00	9.52	O
ATOM	643	CB	TYR	A	39	10.642	-5.527	0.320	1.00	9.60	C
ATOM	644	CG	TYR	A	39	12.025	-5.934	-0.117	1.00	10.26	C
ATOM	645	CD1	TYR	A	39	12.272	-6.325	-1.422	1.00	10.80	C
ATOM	646	CD2	TYR	A	39	13.084	-5.949	0.774	1.00	14.74	C
ATOM	647	CE1	TYR	A	39	13.536	-6.711	-1.834	1.00	11.10	C
ATOM	648	CE2	TYR	A	39	14.356	-6.329	0.379	1.00	15.35	C
ATOM	649	CZ	TYR	A	39	14.583	-6.713	-0.930	1.00	11.62	C
ATOM	650	OH	TYR	A	39	15.833	-7.089	-1.328	1.00	15.47	O
ATOM	660	N	ARG	A	40	8.116	-4.167	1.309	1.00	9.32	N
ATOM	661	CA	ARG	A	40	6.717	-3.718	1.087	1.00	11.91	C
ATOM	662	C	ARG	A	40	6.062	-3.441	2.444	1.00	8.60	C
ATOM	663	O	ARG	A	40	6.735	-2.840	3.307	1.00	12.48	O
ATOM	664	CB	ARG	A	40	5.925	-4.806	0.355	1.00	9.69	C
ATOM	665	CG	ARG	A	40	6.736	-5.657	-0.613	1.00	10.30	C
ATOM	666	CD	ARG	A	40	6.957	-4.990	-1.956	1.00	15.83	C
ATOM	667	NE	ARG	A	40	7.525	-5.909	-2.931	1.00	13.00	N
ATOM	668	CZ	ARG	A	40	8.774	-5.863	-3.377	1.00	16.74	C
ATOM	669	NH1	ARG	A	40	9.596	-4.918	-2.954	1.00	16.86	N
ATOM	670	NH2	ARG	A	40	9.193	-6.751	-4.261	1.00	12.79	N
ATOM	684	N	ASP	A	41	4.806	-3.870	2.623	1.00	8.54	N
ATOM	685	CA	ASP	A	41	4.082	-3.670	3.909	1.00	21.68	C
ATOM	686	C	ASP	A	41	2.934	-4.682	4.014	1.00	8.36	C
ATOM	687	O	ASP	A	41	2.054	-4.660	3.137	1.00	11.20	O
ATOM	688	CB	ASP	A	41	3.559	-2.237	4.030	1.00	31.55	C
ATOM	689	CG	ASP	A	41	2.713	-1.998	5.266	1.00	25.79	C
ATOM	690	OD1	ASP	A	41	3.184	-2.333	6.369	1.00	17.47	O
ATOM	691	OD2	ASP	A	41	1.589	-1.482	5.113	1.00	20.43	O
ATOM	696	N	PRO	A	42	2.887	-5.540	5.059	1.00	8.06	N
ATOM	697	CA	PRO	A	42	1.834	-6.558	5.195	1.00	10.78	C
ATOM	698	C	PRO	A	42	0.421	-5.973	5.351	1.00	12.57	C
ATOM	699	O	PRO	A	42	0.294	-4.893	5.905	1.00	8.10	O
ATOM	700	CB	PRO	A	42	2.211	-7.317	6.479	1.00	12.91	C
ATOM	701	CG	PRO	A	42	3.110	-6.363	7.234	1.00	11.44	C
ATOM	702	CD	PRO	A	42	3.852	-5.598	6.159	1.00	17.98	C
ATOM	710	N	ASP	A	43	-0.600	-6.695	4.870	1.00	8.87	N

```
ATOM    711  CA  ASP A  43      -2.013  -6.242   5.023  1.00  8.99           C
ATOM    712  C   ASP A  43      -2.408  -6.320   6.501  1.00  8.60           C
ATOM    713  O   ASP A  43      -1.699  -7.005   7.267  1.00 11.01           O
ATOM    714  CB  ASP A  43      -2.964  -7.030   4.116  1.00 16.63           C
ATOM    715  CG  ASP A  43      -2.730  -8.530   4.127  1.00 13.16           C
ATOM    716  OD1 ASP A  43      -3.623  -9.255   4.605  1.00 14.21           O
ATOM    717  OD2 ASP A  43      -1.666  -8.963   3.643  1.00 36.15           O
ATOM    722  N   THR A  44      -3.512  -5.674   6.890  1.00  8.55           N
ATOM    723  CA  THR A  44      -3.839  -5.634   8.313  1.00  9.41           C
ATOM    724  C   THR A  44      -4.169  -7.023   8.857  1.00  8.26           C
ATOM    725  O   THR A  44      -3.865  -7.310  10.019  1.00  7.91           O
ATOM    726  CB  THR A  44      -5.006  -4.654   8.547  1.00 11.36           C
ATOM    727  OG1 THR A  44      -6.131  -5.049   7.755  1.00  8.86           O
ATOM    728  CG2 THR A  44      -4.627  -3.234   8.142  1.00  9.58           C
ATOM    736  N   ASP A  45      -4.729  -7.898   8.006  1.00  8.77           N
ATOM    737  CA  ASP A  45      -5.026  -9.303   8.348  1.00 10.61           C
ATOM    738  C   ASP A  45      -3.763 -10.068   8.725  1.00  8.74           C
ATOM    739  O   ASP A  45      -3.699 -10.765   9.757  1.00  8.53           O
ATOM    740  CB  ASP A  45      -5.669  -9.989   7.126  1.00 10.99           C
ATOM    741  CG  ASP A  45      -7.049 -10.496   7.397  1.00 27.01           C
ATOM    742  OD1 ASP A  45      -7.356 -10.769   8.573  1.00 39.19           O
ATOM    743  OD2 ASP A  45      -7.854 -10.545   6.435  1.00 33.70           O
ATOM    748  N   THR A  46      -2.753  -9.956   7.859  1.00  8.80           N
ATOM    749  CA  THR A  46      -1.459 -10.579   8.077  1.00  8.64           C
ATOM    750  C   THR A  46      -0.771  -9.988   9.306  1.00  8.04           C
ATOM    751  O   THR A  46      -0.172 -10.722  10.093  1.00 10.70           O
ATOM    752  CB  THR A  46      -0.660 -10.445   6.783  1.00  8.93           C
ATOM    753  OG1 THR A  46      -1.449 -10.997   5.707  1.00 14.66           O
ATOM    754  CG2 THR A  46       0.647 -11.154   6.826  1.00  8.90           C
ATOM    762  N   LEU A  47      -0.874  -8.667   9.499  1.00  8.24           N
ATOM    763  CA  LEU A  47      -0.278  -8.011  10.656  1.00  7.24           C
ATOM    764  C   LEU A  47      -0.816  -8.594  11.949  1.00  7.48           C
ATOM    765  O   LEU A  47      -0.052  -8.848  12.894  1.00 11.91           O
ATOM    766  CB  LEU A  47      -0.539  -6.497  10.606  1.00  9.58           C
ATOM    767  CG  LEU A  47      -0.020  -5.680  11.812  1.00  6.59           C
ATOM    768  CD1 LEU A  47       1.448  -5.830  11.925  1.00  6.39           C
ATOM    769  CD2 LEU A  47      -0.344  -4.256  11.835  1.00  6.47           C
ATOM    781  N   LEU A  48      -2.149  -8.759  12.034  1.00  7.76           N
ATOM    782  CA  LEU A  48      -2.751  -9.374  13.221  1.00  7.21           C
ATOM    783  C   LEU A  48      -2.346 -10.844  13.384  1.00  7.36           C
ATOM    784  O   LEU A  48      -2.090 -11.304  14.503  1.00  7.15           O
ATOM    785  CB  LEU A  48      -4.270  -9.235  13.169  1.00  7.48           C
ATOM    786  CG  LEU A  48      -5.088  -9.956  14.242  1.00  7.51           C
ATOM    787  CD1 LEU A  48      -4.829  -9.362  15.573  1.00  7.11           C
ATOM    788  CD2 LEU A  48      -6.558  -9.913  13.980  1.00  7.88           C
ATOM    800  N   LYS A  49      -2.323 -11.607  12.299  1.00 11.74           N
ATOM    801  CA  LYS A  49      -1.893 -13.005  12.400  1.00 10.03           C
ATOM    802  C   LYS A  49      -0.468 -13.104  12.937  1.00  7.65           C
ATOM    803  O   LYS A  49      -0.179 -13.880  13.858  1.00  7.57           O
ATOM    804  CB  LYS A  49      -2.005 -13.663  11.025  1.00 15.01           C
ATOM    805  CG  LYS A  49      -3.333 -14.320  10.750  1.00 23.25           C
ATOM    806  CD  LYS A  49      -3.557 -14.629   9.250  1.00 51.38           C
ATOM    807  CE  LYS A  49      -2.550 -15.632   8.666  1.00 30.00           C
ATOM    808  NZ  LYS A  49      -1.222 -14.999   8.358  1.00 25.98           N
ATOM    822  N   LEU A  50       0.444 -12.298  12.387  1.00  7.51           N
ATOM    823  CA  LEU A  50       1.827 -12.351  12.861  1.00  9.78           C
ATOM    824  C   LEU A  50       1.933 -11.913  14.320  1.00  6.82           C
ATOM    825  O   LEU A  50       2.636 -12.548  15.117  1.00 15.01           O
```

ATOM	826	CB	LEU	A	50	2.725	-11.515	11.939	1.00	12.79	C
ATOM	827	CG	LEU	A	50	3.205	-12.180	10.642	1.00	21.27	C
ATOM	828	CD1	LEU	A	50	3.668	-11.141	9.615	1.00	18.46	C
ATOM	829	CD2	LEU	A	50	4.367	-13.203	10.929	1.00	17.36	C
ATOM	841	N	SER	A	51	1.234	-10.843	14.711	1.00	6.60	N
ATOM	842	CA	SER	A	51	1.282	-10.455	16.126	1.00	8.98	C
ATOM	843	C	SER	A	51	0.749	-11.585	17.011	1.00	6.36	C
ATOM	844	O	SER	A	51	1.205	-11.787	18.138	1.00	6.17	O
ATOM	845	CB	SER	A	51	0.499	-9.159	16.368	1.00	8.36	C
ATOM	846	OG	SER	A	51	-0.631	-9.401	17.168	1.00	6.14	O
ATOM	852	N	ASN	A	52	-0.265	-12.292	16.536	1.00	9.15	N
ATOM	853	CA	ASN	A	52	-0.745	-13.461	17.265	1.00	14.89	C
ATOM	854	C	ASN	A	52	0.340	-14.529	17.348	1.00	8.60	C
ATOM	855	O	ASN	A	52	0.572	-15.104	18.413	1.00	6.87	O
ATOM	856	CB	ASN	A	52	-2.018	-13.995	16.606	1.00	9.45	C
ATOM	857	CG	ASN	A	52	-3.269	-13.196	17.001	1.00	7.21	C
ATOM	858	OD1	ASN	A	52	-3.299	-12.518	18.037	1.00	13.80	O
ATOM	859	ND2	ASN	A	52	-4.294	-13.258	16.162	1.00	11.11	N
ATOM	866	N	LEU	A	53	1.061	-14.767	16.250	1.00	12.86	N
ATOM	867	CA	LEU	A	53	2.040	-15.858	16.273	1.00	7.31	C
ATOM	868	C	LEU	A	53	3.206	-15.544	17.174	1.00	7.49	C
ATOM	869	O	LEU	A	53	3.848	-16.465	17.686	1.00	8.09	O
ATOM	870	CB	LEU	A	53	2.592	-16.181	14.887	1.00	7.65	C
ATOM	871	CG	LEU	A	53	3.436	-17.466	14.823	1.00	8.32	C
ATOM	872	CD1	LEU	A	53	2.687	-18.742	15.225	1.00	8.30	C
ATOM	873	CD2	LEU	A	53	3.940	-17.606	13.454	1.00	8.33	C
ATOM	885	N	TYR	A	54	3.565	-14.278	17.300	1.00	7.65	N
ATOM	886	CA	TYR	A	54	4.689	-13.905	18.148	1.00	6.36	C
ATOM	887	C	TYR	A	54	4.302	-13.512	19.557	1.00	11.85	C
ATOM	888	O	TYR	A	54	5.198	-13.267	20.368	1.00	12.81	O
ATOM	889	CB	TYR	A	54	5.439	-12.745	17.527	1.00	6.17	C
ATOM	890	CG	TYR	A	54	5.882	-13.001	16.128	1.00	6.46	C
ATOM	891	CD1	TYR	A	54	6.240	-14.257	15.728	1.00	6.83	C
ATOM	892	CD2	TYR	A	54	5.946	-11.954	15.197	1.00	11.92	C
ATOM	893	CE1	TYR	A	54	6.653	-14.502	14.439	1.00	7.16	C
ATOM	894	CE2	TYR	A	54	6.355	-12.170	13.929	1.00	6.71	C
ATOM	895	CZ	TYR	A	54	6.717	-13.468	13.551	1.00	8.93	C
ATOM	896	OH	TYR	A	54	7.144	-13.734	12.289	1.00	7.47	O
ATOM	906	N	ASN	A	55	3.015	-13.473	19.879	1.00	6.11	N
ATOM	907	CA	ASN	A	55	2.552	-13.054	21.201	1.00	7.26	C
ATOM	908	C	ASN	A	55	2.874	-11.598	21.539	1.00	6.66	C
ATOM	909	O	ASN	A	55	3.190	-11.281	22.685	1.00	7.63	O
ATOM	910	CB	ASN	A	55	3.129	-13.965	22.277	1.00	10.93	C
ATOM	911	CG	ASN	A	55	3.190	-15.398	21.846	1.00	6.29	C
ATOM	912	OD1	ASN	A	55	2.230	-15.957	21.355	1.00	16.09	O
ATOM	913	ND2	ASN	A	55	4.342	-16.006	22.040	1.00	31.82	N
ATOM	920	N	VAL	A	56	2.777	-10.693	20.556	1.00	15.73	N
ATOM	921	CA	VAL	A	56	3.048	-9.276	20.770	1.00	5.29	C
ATOM	922	C	VAL	A	56	1.841	-8.462	20.315	1.00	5.95	C
ATOM	923	O	VAL	A	56	1.010	-8.906	19.521	1.00	7.02	O
ATOM	924	CB	VAL	A	56	4.341	-8.762	20.038	1.00	6.47	C
ATOM	925	CG1	VAL	A	56	5.588	-9.444	20.575	1.00	9.16	C
ATOM	926	CG2	VAL	A	56	4.289	-8.975	18.599	1.00	5.39	C
ATOM	936	N	SER	A	57	1.794	-7.234	20.797	1.00	6.48	N
ATOM	937	CA	SER	A	57	0.746	-6.289	20.447	1.00	12.79	C
ATOM	938	C	SER	A	57	1.028	-5.643	19.098	1.00	5.24	C
ATOM	939	O	SER	A	57	2.176	-5.424	18.715	1.00	5.13	O
ATOM	940	CB	SER	A	57	0.651	-5.208	21.519	1.00	10.47	C

```
ATOM    941  OG  SER A  57       1.835  -4.419  21.563  1.00 12.15           O
ATOM    947  N   THR A  58      -0.029  -5.379  18.349  1.00  5.43           N
ATOM    948  CA  THR A  58       0.163  -4.697  17.068  1.00 11.89           C
ATOM    949  C   THR A  58       0.680  -3.269  17.274  1.00  9.22           C
ATOM    950  O   THR A  58       1.501  -2.786  16.487  1.00  5.27           O
ATOM    951  CB  THR A  58      -1.144  -4.760  16.286  1.00 12.71           C
ATOM    952  OG1 THR A  58      -2.132  -3.954  16.946  1.00  9.09           O
ATOM    953  CG2 THR A  58      -1.646  -6.232  16.230  1.00  6.04           C
ATOM    961  N   ASP A  59       0.258  -2.629  18.376  1.00 15.48           N
ATOM    962  C   ASP A  59       2.359  -1.457  18.938  1.00  4.84           C
ATOM    963  O   ASP A  59       3.077  -0.517  18.597  1.00  7.78           O
ATOM    964  CA  ASP A  59       0.849  -1.365  18.814  1.00 11.39           C
ATOM    965  CB  ASP A  59       0.265  -0.981  20.154  1.00  7.19           C
ATOM    966  CG  ASP A  59      -1.190  -0.679  20.083  1.00  7.14           C
ATOM    967  OD1 ASP A  59      -1.514   0.443  19.758  1.00  8.15           O
ATOM    968  OD2 ASP A  59      -2.028  -1.564  20.329  1.00 15.94           O
ATOM    982  N   TYR A  60       2.855  -2.582  19.470  1.00 12.04           N
ATOM    983  CA  TYR A  60       4.288  -2.757  19.597  1.00  4.66           C
ATOM    984  C   TYR A  60       4.923  -2.805  18.229  1.00  5.03           C
ATOM    985  O   TYR A  60       5.877  -2.065  17.926  1.00  4.65           O
ATOM    986  CB  TYR A  60       4.637  -4.043  20.361  1.00  4.66           C
ATOM    987  CG  TYR A  60       6.059  -4.476  19.983  1.00  4.63           C
ATOM    988  CD1 TYR A  60       7.181  -3.755  20.424  1.00  4.50           C
ATOM    989  CD2 TYR A  60       6.279  -5.527  19.134  1.00  4.79           C
ATOM    990  CE1 TYR A  60       8.509  -4.113  20.038  1.00 11.21           C
ATOM    991  CE2 TYR A  60       7.645  -5.917  18.740  1.00 15.42           C
ATOM    992  CZ  TYR A  60       8.733  -5.187  19.188  1.00  4.69           C
ATOM    993  OH  TYR A  60      10.043  -5.501  18.835  1.00  8.44           O
ATOM   1003  N   LEU A  61       4.406  -3.669  17.368  1.00  6.90           N
ATOM   1004  CA  LEU A  61       5.029  -3.790  16.069  1.00  5.28           C
ATOM   1005  C   LEU A  61       5.010  -2.457  15.328  1.00  8.71           C
ATOM   1006  O   LEU A  61       5.890  -2.191  14.509  1.00 11.18           O
ATOM   1007  CB  LEU A  61       4.329  -4.881  15.240  1.00  8.28           C
ATOM   1008  CG  LEU A  61       4.355  -6.285  15.815  1.00  8.12           C
ATOM   1009  CD1 LEU A  61       3.543  -7.148  14.806  1.00  5.76           C
ATOM   1010  CD2 LEU A  61       5.776  -6.822  16.023  1.00  5.36           C
ATOM   1041  N   LEU A  62       4.006  -1.633  15.572  1.00 12.90           N
ATOM   1042  CA  LEU A  62       3.854  -0.418  14.807  1.00  5.11           C
ATOM   1043  C   LEU A  62       4.707   0.716  15.328  1.00 10.29           C
ATOM   1044  O   LEU A  62       4.890   1.706  14.610  1.00  4.97           O
ATOM   1045  CB  LEU A  62       2.383  -0.001  14.802  1.00  5.52           C
ATOM   1046  CG  LEU A  62       1.390  -0.862  13.980  1.00 10.41           C
ATOM   1047  CD1 LEU A  62       0.065  -0.166  14.036  1.00  5.71           C
ATOM   1048  CD2 LEU A  62       1.783  -1.112  12.571  1.00  5.77           C
ATOM   1061  N   GLY A  63       5.224   0.596  16.544  1.00  4.72           N
ATOM   1062  CA  GLY A  63       5.943   1.695  17.144  1.00  7.49           C
ATOM   1063  C   GLY A  63       5.043   2.697  17.832  1.00 10.24           C
ATOM   1064  O   GLY A  63       5.285   3.903  17.757  1.00  9.96           O
ATOM   1068  N   LYS A  64       3.993   2.213  18.484  1.00 14.24           N
ATOM   1069  CA  LYS A  64       3.169   3.013  19.361  1.00 21.07           C
ATOM   1070  C   LYS A  64       3.685   2.791  20.774  1.00 26.38           C
ATOM   1071  O   LYS A  64       4.518   1.899  21.000  1.00 32.15           O
ATOM   1072  CB  LYS A  64       1.721   2.611  19.231  1.00 25.24           C
ATOM   1073  CG  LYS A  64       0.800   3.503  19.946  1.00 24.95           C
ATOM   1074  CD  LYS A  64      -0.543   3.457  19.271  1.00 11.46           C
ATOM   1075  CE  LYS A  64      -1.520   4.332  20.002  1.00 17.45           C
ATOM   1076  NZ  LYS A  64      -2.528   4.900  19.052  1.00 11.65           N
TER     522      LYS A  64
```

ATOM	1090	N	GLY	B	0	-19.907	-9.529	10.533	1.00	42.89	N
ATOM	1091	CA	GLY	B	0	-20.671	-8.684	11.490	1.00	18.79	C
ATOM	1092	C	GLY	B	0	-19.886	-7.492	12.033	1.00	14.15	C
ATOM	1093	O	GLY	B	0	-20.366	-6.373	11.914	1.00	16.83	O
ATOM	1099	N	MET	B	1	-18.711	-7.724	12.635	1.00	13.51	N
ATOM	1100	CA	MET	B	1	-17.854	-6.659	13.145	1.00	18.66	C
ATOM	1101	C	MET	B	1	-16.548	-6.631	12.352	1.00	12.54	C
ATOM	1102	O	MET	B	1	-16.128	-7.642	11.780	1.00	9.60	O
ATOM	1103	CB	MET	B	1	-17.552	-6.808	14.647	1.00	6.53	C
ATOM	1104	CG	MET	B	1	-16.932	-5.530	15.235	1.00	6.56	C
ATOM	1105	SD	MET	B	1	-17.978	-4.031	14.995	1.00	19.20	S
ATOM	1106	CE	MET	B	1	-19.042	-4.240	16.402	1.00	23.80	C
ATOM	1116	N	SER	B	2	-15.944	-5.442	12.261	1.00	12.87	N
ATOM	1117	CA	SER	B	2	-14.867	-5.224	11.303	1.00	16.18	C
ATOM	1118	C	SER	B	2	-14.024	-4.047	11.762	1.00	6.11	C
ATOM	1119	O	SER	B	2	-14.436	-3.273	12.622	1.00	6.09	O
ATOM	1120	CB	SER	B	2	-15.408	-4.931	9.894	1.00	7.33	C
ATOM	1121	OG	SER	B	2	-15.987	-3.642	9.856	1.00	6.54	O
ATOM	1127	N	LEU	B	3	-12.848	-3.903	11.127	1.00	6.05	N
ATOM	1128	CA	LEU	B	3	-12.007	-2.737	11.355	1.00	6.00	C
ATOM	1129	C	LEU	B	3	-12.737	-1.447	11.008	1.00	6.14	C
ATOM	1130	O	LEU	B	3	-12.651	-0.471	11.742	1.00	6.13	O
ATOM	1131	CB	LEU	B	3	-10.700	-2.852	10.557	1.00	7.73	C
ATOM	1132	CG	LEU	B	3	-9.775	-1.624	10.506	1.00	6.04	C
ATOM	1133	CD1	LEU	B	3	-9.177	-1.297	11.829	1.00	5.97	C
ATOM	1134	CD2	LEU	B	3	-8.664	-1.797	9.490	1.00	15.53	C
ATOM	1146	N	GLY	B	4	-13.425	-1.409	9.869	1.00	6.31	N
ATOM	1147	CA	GLY	B	4	-14.206	-0.254	9.484	1.00	6.51	C
ATOM	1148	C	GLY	B	4	-15.200	0.227	10.531	1.00	9.95	C
ATOM	1149	O	GLY	B	4	-15.389	1.435	10.753	1.00	7.21	O
ATOM	1153	N	MLZ	B	5	-15.824	-0.734	11.200	1.00	10.91	N
ATOM	1154	CA	MLZ	B	5	-16.912	-0.436	12.170	1.00	8.46	C
ATOM	1155	C	MLZ	B	5	-16.278	0.105	13.460	1.00	10.73	C
ATOM	1156	O	MLZ	B	5	-16.833	1.082	14.008	1.00	6.42	O
ATOM	1157	CB	MLZ	B	5	-17.771	-1.681	12.408	1.00	6.56	C
ATOM	1158	CG	MLZ	B	5	-18.709	-2.078	11.267	1.00	7.90	C
ATOM	1159	CD	MLZ	B	5	-19.693	-3.161	11.687	1.00	12.43	C
ATOM	1160	CE	MLZ	B	5	-20.904	-3.388	10.796	1.00	21.39	C
ATOM	1161	NZ	MLZ	B	5	-21.892	-4.249	11.464	1.00	22.11	N
ATOM	1162	CM	MLZ	B	5	-22.740	-3.553	12.454	1.00	17.69	C
ATOM	1177	N	ARG	B	6	-15.137	-0.442	13.889	1.00	6.16	N
ATOM	1178	CA	ARG	B	6	-14.435	0.109	15.032	1.00	6.08	C
ATOM	1179	C	ARG	B	6	-13.798	1.467	14.672	1.00	14.80	C
ATOM	1180	O	ARG	B	6	-13.666	2.302	15.544	1.00	6.19	O
ATOM	1181	CB	ARG	B	6	-13.373	-0.842	15.554	1.00	10.13	C
ATOM	1182	CG	ARG	B	6	-13.919	-2.119	16.154	1.00	5.93	C
ATOM	1183	CD	ARG	B	6	-12.808	-2.945	16.721	1.00	14.35	C
ATOM	1184	NE	ARG	B	6	-13.263	-4.301	17.012	1.00	13.51	N
ATOM	1185	CZ	ARG	B	6	-13.286	-5.288	16.126	1.00	6.73	C
ATOM	1186	NH1	ARG	B	6	-12.779	-5.150	14.910	1.00	18.18	N
ATOM	1187	NH2	ARG	B	6	-13.865	-6.426	16.451	1.00	6.03	N
ATOM	1201	N	LEU	B	7	-13.362	1.692	13.425	1.00	6.23	N
ATOM	1202	CA	LEU	B	7	-12.851	3.022	13.080	1.00	6.38	C
ATOM	1203	C	LEU	B	7	-13.966	4.051	13.157	1.00	6.57	C
ATOM	1204	O	LEU	B	7	-13.797	5.129	13.733	1.00	6.68	O
ATOM	1205	CB	LEU	B	7	-12.234	3.032	11.701	1.00	6.48	C
ATOM	1206	CG	LEU	B	7	-10.875	2.395	11.487	1.00	9.03	C
ATOM	1207	CD1	LEU	B	7	-10.686	2.294	9.993	1.00	6.53	C

ATOM	1208	CD2	LEU	B	7	-9.763	3.160	12.151	1.00	6.44	C
ATOM	1220	N	LYS	B	8	-15.133	3.726	12.595	1.00	12.91	N
ATOM	1221	CA	LYS	B	8	-16.269	4.634	12.737	1.00	12.99	C
ATOM	1222	C	LYS	B	8	-16.547	4.915	14.205	1.00	6.79	C
ATOM	1223	O	LYS	B	8	-16.606	6.071	14.629	1.00	14.31	O
ATOM	1224	CB	LYS	B	8	-17.512	4.030	12.072	1.00	13.46	C
ATOM	1225	CG	LYS	B	8	-18.760	4.894	12.174	1.00	35.34	C
ATOM	1226	CD	LYS	B	8	-19.874	4.378	11.277	1.00	46.24	C
ATOM	1227	CE	LYS	B	8	-19.462	4.364	9.807	1.00	26.67	C
ATOM	1228	NZ	LYS	B	8	-20.646	4.316	8.905	1.00	32.99	N
ATOM	1242	N	GLU	B	9	-16.711	3.862	14.995	1.00	6.60	N
ATOM	1243	CA	GLU	B	9	-17.064	4.031	16.400	1.00	17.04	C
ATOM	1244	C	GLU	B	9	-15.992	4.809	17.167	1.00	14.28	C
ATOM	1245	O	GLU	B	9	-16.322	5.657	18.004	1.00	9.09	O
ATOM	1246	CB	GLU	B	9	-17.345	2.652	17.044	1.00	13.67	C
ATOM	1247	CG	GLU	B	9	-17.453	2.725	18.587	1.00	24.10	C
ATOM	1248	CD	GLU	B	9	-18.251	1.583	19.246	1.00	29.05	C
ATOM	1249	OE1	GLU	B	9	-18.717	0.649	18.554	1.00	40.79	O
ATOM	1250	OE2	GLU	B	9	-18.358	1.585	20.491	1.00	38.73	O
ATOM	1257	N	ALA	B	10	-14.703	4.534	16.915	1.00	6.46	N
ATOM	1258	CA	ALA	B	10	-13.651	5.262	17.613	1.00	6.52	C
ATOM	1259	C	ALA	B	10	-13.674	6.739	17.239	1.00	11.34	C
ATOM	1260	O	ALA	B	10	-13.525	7.599	18.111	1.00	6.91	O
ATOM	1261	CB	ALA	B	10	-12.290	4.618	17.303	1.00	6.42	C
ATOM	1267	N	ARG	B	11	-13.890	7.051	15.941	1.00	17.81	N
ATOM	1268	CA	ARG	B	11	-14.003	8.443	15.478	1.00	10.32	C
ATOM	1269	C	ARG	B	11	-15.180	9.140	16.129	1.00	7.32	C
ATOM	1270	O	ARG	B	11	-15.079	10.297	16.533	1.00	7.56	O
ATOM	1271	CB	ARG	B	11	-14.165	8.487	13.949	1.00	12.82	C
ATOM	1272	CG	ARG	B	11	-14.454	9.878	13.378	1.00	9.55	C
ATOM	1273	CD	ARG	B	11	-14.630	9.881	11.836	1.00	14.21	C
ATOM	1274	NE	ARG	B	11	-15.625	8.890	11.457	1.00	19.00	N
ATOM	1275	CZ	ARG	B	11	-16.930	9.079	11.551	1.00	11.90	C
ATOM	1276	NH1	ARG	B	11	-17.429	10.221	12.006	1.00	10.94	N
ATOM	1277	NH2	ARG	B	11	-17.755	8.092	11.213	1.00	17.83	N
ATOM	1291	N	GLN	B	12	-16.302	8.429	16.250	1.00	10.44	N
ATOM	1292	CA	GLN	B	12	-17.458	8.994	16.919	1.00	7.81	C
ATOM	1293	C	GLN	B	12	-17.145	9.245	18.375	1.00	7.32	C
ATOM	1294	O	GLN	B	12	-17.427	10.326	18.886	1.00	11.08	O
ATOM	1295	CB	GLN	B	12	-18.671	8.102	16.716	1.00	7.31	C
ATOM	1296	CG	GLN	B	12	-19.094	8.139	15.229	1.00	7.49	C
ATOM	1297	CD	GLN	B	12	-20.309	7.295	14.849	1.00	15.53	C
ATOM	1298	OE1	GLN	B	12	-20.742	6.404	15.582	1.00	7.40	O
ATOM	1299	NE2	GLN	B	12	-20.854	7.578	13.660	1.00	25.06	N
ATOM	1308	N	LYS	B	13	-16.444	8.327	19.033	1.00	7.09	N
ATOM	1309	CA	LYS	B	13	-16.073	8.591	20.422	1.00	7.13	C
ATOM	1310	C	LYS	B	13	-15.181	9.808	20.548	1.00	10.40	C
ATOM	1311	O	LYS	B	13	-15.311	10.558	21.519	1.00	7.54	O
ATOM	1312	CB	LYS	B	13	-15.388	7.390	21.053	1.00	10.99	C
ATOM	1313	CG	LYS	B	13	-16.352	6.418	21.605	1.00	7.19	C
ATOM	1314	CD	LYS	B	13	-15.634	5.323	22.337	1.00	26.72	C
ATOM	1315	CE	LYS	B	13	-16.542	4.137	22.574	1.00	18.01	C
ATOM	1316	NZ	LYS	B	13	-17.352	3.824	21.358	1.00	33.05	N
ATOM	1330	N	ALA	B	14	-14.255	10.024	19.585	1.00	16.58	N
ATOM	1331	CA	ALA	B	14	-13.328	11.159	19.691	1.00	20.04	C
ATOM	1332	C	ALA	B	14	-14.006	12.498	19.400	1.00	13.33	C
ATOM	1333	O	ALA	B	14	-13.483	13.542	19.788	1.00	14.64	O
ATOM	1334	CB	ALA	B	14	-12.119	10.968	18.749	1.00	7.69	C

ATOM	1340	N	GLY	B	15	-15.156	12.480	18.741	1.00	15.53	N
ATOM	1341	CA	GLY	B	15	-15.938	13.654	18.487	1.00	8.36	C
ATOM	1342	C	GLY	B	15	-15.874	14.158	17.082	1.00	11.47	C
ATOM	1343	O	GLY	B	15	-16.651	15.063	16.749	1.00	17.22	O
ATOM	1347	N	TYR	B	16	-15.010	13.587	16.236	1.00	8.47	N
ATOM	1348	CA	TYR	B	16	-14.835	14.088	14.876	1.00	10.74	C
ATOM	1349	C	TYR	B	16	-15.935	13.595	13.961	1.00	8.73	C
ATOM	1350	O	TYR	B	16	-16.348	12.441	14.047	1.00	8.40	O
ATOM	1351	CB	TYR	B	16	-13.500	13.654	14.235	1.00	13.57	C
ATOM	1352	CG	TYR	B	16	-12.298	14.131	14.953	1.00	8.80	C
ATOM	1353	CD1	TYR	B	16	-11.735	13.367	15.944	1.00	8.50	C
ATOM	1354	CD2	TYR	B	16	-11.735	15.363	14.677	1.00	14.54	C
ATOM	1355	CE1	TYR	B	16	-10.674	13.782	16.636	1.00	8.66	C
ATOM	1356	CE2	TYR	B	16	-10.634	15.786	15.378	1.00	10.55	C
ATOM	1357	CZ	TYR	B	16	-10.117	14.986	16.370	1.00	9.13	C
ATOM	1358	OH	TYR	B	16	-9.008	15.353	17.096	1.00	24.63	O
ATOM	1368	N	THR	B	17	-16.301	14.424	12.979	1.00	16.84	N
ATOM	1369	CA	THR	B	17	-17.229	13.934	11.927	1.00	9.27	C
ATOM	1370	C	THR	B	17	-16.349	13.286	10.848	1.00	12.05	C
ATOM	1371	O	THR	B	17	-15.110	13.444	10.932	1.00	11.12	O
ATOM	1372	CB	THR	B	17	-18.112	15.057	11.366	1.00	15.07	C
ATOM	1373	OG1	THR	B	17	-17.315	15.892	10.524	1.00	10.35	O
ATOM	1374	CG2	THR	B	17	-18.768	15.887	12.447	1.00	9.98	C
ATOM	1382	N	GLN	B	18	-16.939	12.603	9.865	1.00	11.31	N
ATOM	1383	CA	GLN	B	18	-16.114	11.876	8.857	1.00	9.65	C
ATOM	1384	C	GLN	B	18	-15.161	12.856	8.157	1.00	9.91	C
ATOM	1385	O	GLN	B	18	-13.972	12.507	8.010	1.00	9.74	O
ATOM	1386	CB	GLN	B	18	-17.019	11.138	7.868	1.00	24.75	C
ATOM	1387	CG	GLN	B	18	-16.290	10.542	6.673	1.00	9.74	C
ATOM	1388	CD	GLN	B	18	-17.252	10.099	5.597	1.00	10.90	C
ATOM	1389	OE1	GLN	B	18	-17.082	10.408	4.420	1.00	15.27	O
ATOM	1390	NE2	GLN	B	18	-18.286	9.379	5.999	1.00	8.79	N
ATOM	1399	N	LYS	B	19	-15.651	14.036	7.761	1.00	13.42	N
ATOM	1400	CA	LYS	B	19	-14.808	15.012	7.011	1.00	29.21	C
ATOM	1401	C	LYS	B	19	-13.719	15.609	7.913	1.00	21.33	C
ATOM	1402	O	LYS	B	19	-12.566	15.682	7.451	1.00	10.78	O
ATOM	1403	CB	LYS	B	19	-15.676	16.121	6.408	1.00	23.08	C
ATOM	1404	CG	LYS	B	19	-16.497	15.710	5.194	1.00	24.35	C
ATOM	1405	CD	LYS	B	19	-16.263	16.580	3.975	1.00	43.07	C
ATOM	1406	CE	LYS	B	19	-14.800	16.763	3.634	1.00	27.89	C
ATOM	1407	NZ	LYS	B	19	-14.621	17.553	2.393	1.00	31.21	N
ATOM	1421	N	GLU	B	20	-14.071	16.034	9.131	1.00	10.63	N
ATOM	1422	CA	GLU	B	20	-13.086	16.684	10.042	1.00	10.38	C
ATOM	1423	C	GLU	B	20	-11.854	15.789	10.189	1.00	10.03	C
ATOM	1424	O	GLU	B	20	-10.733	16.301	10.011	1.00	11.50	O
ATOM	1425	CB	GLU	B	20	-13.718	16.942	11.410	1.00	18.01	C
ATOM	1426	CG	GLU	B	20	-14.836	17.967	11.378	1.00	18.00	C
ATOM	1427	CD	GLU	B	20	-15.623	18.096	12.672	1.00	17.81	C
ATOM	1428	OE1	GLU	B	20	-15.304	17.370	13.635	1.00	10.09	O
ATOM	1429	OE2	GLU	B	20	-16.557	18.920	12.711	1.00	11.04	O
ATOM	1436	N	ALA	B	21	-12.059	14.508	10.503	1.00	14.53	N
ATOM	1437	CA	ALA	B	21	-10.956	13.555	10.680	1.00	9.17	C
ATOM	1438	C	ALA	B	21	-10.102	13.416	9.432	1.00	9.96	C
ATOM	1439	O	ALA	B	21	-8.871	13.306	9.519	1.00	9.38	O
ATOM	1440	CB	ALA	B	21	-11.523	12.202	11.060	1.00	8.65	C
ATOM	1446	N	ALA	B	22	-10.751	13.318	8.270	1.00	11.10	N
ATOM	1447	CA	ALA	B	22	-10.028	13.209	7.012	1.00	12.35	C
ATOM	1448	C	ALA	B	22	-9.168	14.436	6.772	1.00	18.26	C

ATOM	1449	O	ALA	B	22	-7.997	14.327	6.392	1.00	25.70	O
ATOM	1450	CB	ALA	B	22	-11.021	13.005	5.867	1.00	17.57	C
ATOM	1456	N	GLU	B	23	-9.730	15.622	6.988	1.00	10.70	N
ATOM	1457	CA	GLU	B	23	-8.962	16.835	6.768	1.00	11.31	C
ATOM	1458	C	GLU	B	23	-7.782	16.916	7.722	1.00	19.67	C
ATOM	1459	O	GLU	B	23	-6.653	17.177	7.298	1.00	20.87	O
ATOM	1460	CB	GLU	B	23	-9.872	18.049	6.936	1.00	15.91	C
ATOM	1461	CG	GLU	B	23	-10.936	18.158	5.869	1.00	32.91	C
ATOM	1462	CD	GLU	B	23	-11.871	19.331	6.100	1.00	47.30	C
ATOM	1463	OE1	GLU	B	23	-11.800	19.940	7.195	1.00	43.74	O
ATOM	1464	OE2	GLU	B	23	-12.663	19.639	5.182	1.00	41.47	O
ATOM	1471	N	LYS	B	24	-8.008	16.619	9.004	1.00	18.89	N
ATOM	1472	CA	LYS	B	24	-6.905	16.604	9.956	1.00	20.97	C
ATOM	1473	C	LYS	B	24	-5.817	15.607	9.562	1.00	18.79	C
ATOM	1474	O	LYS	B	24	-4.627	15.871	9.764	1.00	19.30	O
ATOM	1475	CB	LYS	B	24	-7.449	16.304	11.341	1.00	10.39	C
ATOM	1476	CG	LYS	B	24	-7.930	17.535	12.074	1.00	40.26	C
ATOM	1477	CD	LYS	B	24	-7.354	17.603	13.453	1.00	10.70	C
ATOM	1478	CE	LYS	B	24	-5.815	17.650	13.428	1.00	25.48	C
ATOM	1479	NZ	LYS	B	24	-5.283	18.343	14.653	1.00	47.87	N
ATOM	1493	N	LEU	B	25	-6.194	14.455	9.006	1.00	27.76	N
ATOM	1494	CA	LEU	B	25	-5.221	13.442	8.627	1.00	9.94	C
ATOM	1495	C	LEU	B	25	-4.745	13.596	7.200	1.00	23.10	C
ATOM	1496	O	LEU	B	25	-3.935	12.786	6.740	1.00	34.86	O
ATOM	1497	CB	LEU	B	25	-5.803	12.045	8.851	1.00	9.32	C
ATOM	1498	CG	LEU	B	25	-5.878	11.646	10.333	1.00	32.33	C
ATOM	1499	CD1	LEU	B	25	-6.291	10.173	10.556	1.00	20.96	C
ATOM	1500	CD2	LEU	B	25	-4.538	11.938	11.059	1.00	13.43	C
ATOM	1512	N	ASN	B	26	-5.194	14.662	6.524	1.00	25.77	N
ATOM	1513	CA	ASN	B	26	-4.766	14.954	5.126	1.00	38.27	C
ATOM	1514	C	ASN	B	26	-5.176	13.805	4.193	1.00	36.21	C
ATOM	1515	O	ASN	B	26	-4.853	13.886	2.991	1.00	32.33	O
ATOM	1516	CB	ASN	B	26	-3.271	15.286	5.037	1.00	34.91	C
ATOM	1517	CG	ASN	B	26	-2.942	16.275	3.939	1.00	44.05	C
ATOM	1518	OD1	ASN	B	26	-3.832	16.908	3.376	1.00	43.15	O
ATOM	1519	ND2	ASN	B	26	-1.664	16.419	3.632	1.00	50.92	N
ATOM	1526	N	ILE	B	27	-5.863	12.785	4.717	1.00	13.10	N
ATOM	1527	CA	ILE	B	27	-6.371	11.685	3.843	1.00	20.62	C
ATOM	1528	C	ILE	B	27	-7.647	12.170	3.146	1.00	22.88	C
ATOM	1529	O	ILE	B	27	-8.277	13.124	3.651	1.00	52.00	O
ATOM	1530	CB	ILE	B	27	-6.610	10.388	4.646	1.00	40.02	C
ATOM	1531	CG1	ILE	B	27	-7.702	10.559	5.705	1.00	20.54	C
ATOM	1532	CG2	ILE	B	27	-5.311	9.881	5.253	1.00	41.15	C
ATOM	1533	CD1	ILE	B	27	-8.055	9.285	6.435	1.00	15.33	C
ATOM	1545	N	GLY	B	28	-8.010	11.543	2.025	1.00	27.39	N
ATOM	1546	CA	GLY	B	28	-9.251	11.917	1.320	1.00	44.26	C
ATOM	1547	C	GLY	B	28	-10.475	11.665	2.182	1.00	41.27	C
ATOM	1548	O	GLY	B	28	-10.462	10.685	2.953	1.00	17.55	O
ATOM	1552	N	ASN	B	29	-11.494	12.522	2.076	1.00	25.64	N
ATOM	1553	CA	ASN	B	29	-12.759	12.282	2.820	1.00	35.26	C
ATOM	1554	C	ASN	B	29	-13.341	10.947	2.356	1.00	30.26	C
ATOM	1555	O	ASN	B	29	-13.745	10.143	3.221	1.00	22.26	O
ATOM	1556	CB	ASN	B	29	-13.769	13.408	2.607	1.00	20.47	C
ATOM	1557	CG	ASN	B	29	-15.119	13.100	3.216	1.00	34.50	C
ATOM	1558	OD1	ASN	B	29	-16.143	13.192	2.545	1.00	25.46	O
ATOM	1559	ND2	ASN	B	29	-15.129	12.728	4.485	1.00	43.06	N
ATOM	1566	N	ASN	B	30	-13.358	10.722	1.040	1.00	41.23	N
ATOM	1567	CA	ASN	B	30	-13.872	9.449	0.473	1.00	55.56	C

ATOM	1568	C	ASN	B	30	-13.006	8.282	0.956	1.00	31.59	C
ATOM	1569	O	ASN	B	30	-13.543	7.169	1.073	1.00	24.29	O
ATOM	1570	CB	ASN	B	30	-13.903	9.508	-1.052	1.00	35.60	C
ATOM	1571	CG	ASN	B	30	-12.532	9.712	-1.660	1.00	54.81	C
ATOM	1572	OD1	ASN	B	30	-12.010	10.824	-1.665	1.00	48.85	O
ATOM	1573	ND2	ASN	B	30	-11.940	8.641	-2.164	1.00	53.09	N
ATOM	1580	N	ASN	B	31	-11.716	8.535	1.202	1.00	27.85	N
ATOM	1581	CA	ASN	B	31	-10.793	7.465	1.667	1.00	24.35	C
ATOM	1582	C	ASN	B	31	-11.238	7.012	3.060	1.00	17.91	C
ATOM	1583	O	ASN	B	31	-11.400	5.795	3.246	1.00	8.46	O
ATOM	1584	CB	ASN	B	31	-9.328	7.910	1.624	1.00	36.08	C
ATOM	1585	CG	ASN	B	31	-8.868	8.274	0.228	1.00	29.34	C
ATOM	1586	OD1	ASN	B	31	-9.584	8.048	-0.745	1.00	44.91	O
ATOM	1587	ND2	ASN	B	31	-7.678	8.838	0.117	1.00	29.91	N
ATOM	1594	N	LEU	B	32	-11.454	7.953	3.987	1.00	9.90	N
ATOM	1595	CA	LEU	B	32	-11.972	7.574	5.324	1.00	9.81	C
ATOM	1596	C	LEU	B	32	-13.297	6.831	5.128	1.00	13.28	C
ATOM	1597	O	LEU	B	32	-13.524	5.842	5.846	1.00	8.00	O
ATOM	1598	CB	LEU	B	32	-12.150	8.827	6.185	1.00	11.63	C
ATOM	1599	CG	LEU	B	32	-12.412	8.553	7.665	1.00	8.20	C
ATOM	1600	CD1	LEU	B	32	-11.487	7.466	8.189	1.00	11.86	C
ATOM	1601	CD2	LEU	B	32	-12.255	9.820	8.484	1.00	8.42	C
ATOM	1613	N	SER	B	33	-14.120	7.271	4.169	1.00	17.21	N
ATOM	1614	CA	SER	B	33	-15.410	6.625	3.994	1.00	11.52	C
ATOM	1615	C	SER	B	33	-15.229	5.180	3.544	1.00	8.60	C
ATOM	1616	O	SER	B	33	-15.894	4.277	4.046	1.00	8.39	O
ATOM	1617	CB	SER	B	33	-16.223	7.428	2.969	1.00	9.40	C
ATOM	1618	OG	SER	B	33	-17.452	6.805	2.705	1.00	9.52	O
ATOM	1624	N	ASN	B	34	-14.319	4.952	2.596	1.00	8.71	N
ATOM	1625	CA	ASN	B	34	-14.003	3.617	2.117	1.00	8.55	C
ATOM	1626	C	ASN	B	34	-13.470	2.725	3.236	1.00	8.03	C
ATOM	1627	O	ASN	B	34	-13.803	1.539	3.303	1.00	7.87	O
ATOM	1628	CB	ASN	B	34	-13.020	3.750	0.974	1.00	8.79	C
ATOM	1629	CG	ASN	B	34	-13.700	4.189	-0.323	1.00	9.37	C
ATOM	1630	OD1	ASN	B	34	-14.922	4.121	-0.456	1.00	9.57	O
ATOM	1631	ND2	ASN	B	34	-12.911	4.638	-1.269	1.00	9.69	N
ATOM	1638	N	TYR	B	35	-12.637	3.297	4.111	1.00	8.92	N
ATOM	1639	CA	TYR	B	35	-12.044	2.506	5.222	1.00	7.40	C
ATOM	1640	C	TYR	B	35	-13.146	2.095	6.202	1.00	7.22	C
ATOM	1641	O	TYR	B	35	-13.183	0.909	6.582	1.00	7.00	O
ATOM	1642	CB	TYR	B	35	-10.982	3.310	5.977	1.00	7.32	C
ATOM	1643	CG	TYR	B	35	-9.841	3.842	5.147	1.00	7.52	C
ATOM	1644	CD1	TYR	B	35	-9.440	3.212	3.980	1.00	7.59	C
ATOM	1645	CD2	TYR	B	35	-9.146	4.973	5.543	1.00	16.36	C
ATOM	1646	CE1	TYR	B	35	-8.392	3.704	3.220	1.00	10.15	C
ATOM	1647	CE2	TYR	B	35	-8.095	5.476	4.795	1.00	9.81	C
ATOM	1648	CZ	TYR	B	35	-7.714	4.837	3.629	1.00	7.99	C
ATOM	1649	OH	TYR	B	35	-6.677	5.325	2.888	1.00	8.27	O
ATOM	1659	N	GLU	B	36	-14.013	3.037	6.586	1.00	7.35	N
ATOM	1660	CA	GLU	B	36	-15.063	2.753	7.602	1.00	15.51	C
ATOM	1661	C	GLU	B	36	-16.199	1.935	6.975	1.00	8.04	C
ATOM	1662	O	GLU	B	36	-17.105	1.523	7.727	1.00	13.01	O
ATOM	1663	CB	GLU	B	36	-15.561	4.061	8.219	1.00	16.29	C
ATOM	1664	CG	GLU	B	36	-14.491	4.765	9.033	1.00	7.38	C
ATOM	1665	CD	GLU	B	36	-14.882	6.110	9.619	1.00	11.74	C
ATOM	1666	OE1	GLU	B	36	-15.894	6.682	9.168	1.00	11.33	O
ATOM	1667	OE2	GLU	B	36	-14.172	6.580	10.530	1.00	7.60	O
ATOM	1674	N	ARG	B	37	-16.148	1.693	5.662	1.00	11.56	N

ATOM	1675	CA	ARG	B	37	-17.181	0.849	5.000	1.00	17.15	C
ATOM	1676	C	ARG	B	37	-16.550	-0.466	4.527	1.00	18.85	C
ATOM	1677	O	ARG	B	37	-17.235	-1.195	3.779	1.00	14.25	O
ATOM	1678	CB	ARG	B	37	-17.838	1.619	3.851	1.00	18.27	C
ATOM	1679	CG	ARG	B	37	-18.958	2.548	4.298	1.00	8.98	C
ATOM	1680	CD	ARG	B	37	-19.166	3.697	3.333	1.00	29.35	C
ATOM	1681	NE	ARG	B	37	-19.194	3.258	1.947	1.00	47.65	N
ATOM	1682	CZ	ARG	B	37	-19.305	4.070	0.904	1.00	51.77	C
ATOM	1683	NH1	ARG	B	37	-19.399	5.376	1.085	1.00	54.94	N
ATOM	1684	NH2	ARG	B	37	-19.319	3.573	-0.319	1.00	29.22	N
ATOM	1698	N	ASP	B	38	-15.300	-0.741	4.923	1.00	7.37	N
ATOM	1699	CA	ASP	B	38	-14.610	-2.018	4.575	1.00	13.35	C
ATOM	1700	C	ASP	B	38	-14.387	-2.128	3.061	1.00	8.68	C
ATOM	1701	O	ASP	B	38	-14.097	-3.249	2.596	1.00	7.57	O
ATOM	1702	CB	ASP	B	38	-15.361	-3.234	5.128	1.00	7.12	C
ATOM	1703	CG	ASP	B	38	-15.611	-3.154	6.622	1.00	6.84	C
ATOM	1704	OD1	ASP	B	38	-14.684	-2.746	7.347	1.00	6.61	O
ATOM	1705	OD2	ASP	B	38	-16.736	-3.484	7.045	1.00	10.85	O
ATOM	1710	N	TYR	B	39	-14.509	-1.019	2.324	1.00	7.83	N
ATOM	1711	CA	TYR	B	39	-14.228	-1.038	0.865	1.00	8.17	C
ATOM	1712	C	TYR	B	39	-12.732	-1.284	0.654	1.00	15.59	C
ATOM	1713	O	TYR	B	39	-12.382	-2.184	-0.134	1.00	13.92	O
ATOM	1714	CB	TYR	B	39	-14.663	0.277	0.216	1.00	8.62	C
ATOM	1715	CG	TYR	B	39	-16.099	0.323	-0.241	1.00	21.43	C
ATOM	1716	CD1	TYR	B	39	-16.416	0.539	-1.572	1.00	44.24	C
ATOM	1717	CD2	TYR	B	39	-17.142	0.146	0.651	1.00	46.06	C
ATOM	1718	CE1	TYR	B	39	-17.732	0.583	-2.003	1.00	42.31	C
ATOM	1719	CE2	TYR	B	39	-18.463	0.184	0.238	1.00	38.64	C
ATOM	1720	CZ	TYR	B	39	-18.760	0.403	-1.095	1.00	38.83	C
ATOM	1721	OH	TYR	B	39	-20.059	0.445	-1.511	1.00	22.78	O
ATOM	1731	N	ARG	B	40	-11.894	-0.469	1.301	1.00	9.74	N
ATOM	1732	C	ARG	B	40	-9.845	-1.068	2.514	1.00	7.32	C
ATOM	1733	O	ARG	B	40	-10.633	-1.493	3.386	1.00	8.32	O
ATOM	1755	CA	ARG	B	40	-10.420	-0.613	1.170	1.00	7.72	C
ATOM	1756	CB	ARG	B	40	-9.785	0.714	0.742	1.00	11.97	C
ATOM	1757	CG	ARG	B	40	-10.494	1.417	-0.407	1.00	13.09	C
ATOM	1758	CD	ARG	B	40	-10.548	0.616	-1.694	1.00	18.77	C
ATOM	1759	NE	ARG	B	40	-11.881	0.652	-2.280	1.00	19.18	N
ATOM	1760	CZ	ARG	B	40	-12.308	1.561	-3.150	1.00	14.14	C
ATOM	1761	NH1	ARG	B	40	-11.502	2.528	-3.556	1.00	11.05	N
ATOM	1762	NH2	ARG	B	40	-13.543	1.499	-3.615	1.00	8.86	N
ATOM	1776	N	ASP	B	41	-8.523	-0.977	2.673	1.00	8.84	N
ATOM	1777	CA	ASP	B	41	-7.866	-1.377	3.945	1.00	8.37	C
ATOM	1778	C	ASP	B	41	-6.701	-0.421	4.208	1.00	7.01	C
ATOM	1779	O	ASP	B	41	-5.647	-0.602	3.575	1.00	7.22	O
ATOM	1780	CB	ASP	B	41	-7.429	-2.842	3.887	1.00	6.83	C
ATOM	1781	CG	ASP	B	41	-6.872	-3.372	5.193	1.00	9.11	C
ATOM	1782	OD1	ASP	B	41	-7.344	-2.924	6.257	1.00	6.44	O
ATOM	1783	OD2	ASP	B	41	-5.968	-4.229	5.132	1.00	9.48	O
ATOM	1788	N	PRO	B	42	-6.816	0.545	5.146	1.00	6.90	N
ATOM	1789	CA	PRO	B	42	-5.767	1.555	5.353	1.00	18.06	C
ATOM	1790	C	PRO	B	42	-4.372	0.942	5.547	1.00	7.03	C
ATOM	1791	O	PRO	B	42	-4.259	-0.030	6.279	1.00	6.80	O
ATOM	1792	CB	PRO	B	42	-6.202	2.285	6.636	1.00	17.15	C
ATOM	1793	CG	PRO	B	42	-7.254	1.389	7.259	1.00	6.63	C
ATOM	1794	CD	PRO	B	42	-7.920	0.690	6.093	1.00	6.68	C
ATOM	1802	N	ASP	B	43	-3.352	1.521	4.900	1.00	8.95	N
ATOM	1803	CA	ASP	B	43	-1.955	1.031	5.072	1.00	7.89	C

ATOM	1804	C	ASP	B	43	-1.573	1.135	6.552	1.00	7.25	C
ATOM	1805	O	ASP	B	43	-2.197	1.939	7.270	1.00	7.20	O
ATOM	1806	CB	ASP	B	43	-0.974	1.741	4.132	1.00	10.65	C
ATOM	1807	CG	ASP	B	43	-1.082	3.254	4.150	1.00	8.05	C
ATOM	1808	OD1	ASP	B	43	-0.243	3.892	4.812	1.00	8.61	O
ATOM	1809	OD2	ASP	B	43	-2.000	3.784	3.493	1.00	24.47	O
ATOM	1814	N	THR	B	44	-0.560	0.385	6.989	1.00	9.79	N
ATOM	1815	CA	THR	B	44	-0.255	0.347	8.426	1.00	18.89	C
ATOM	1816	C	THR	B	44	0.099	1.726	8.987	1.00	7.41	C
ATOM	1817	O	THR	B	44	-0.172	2.000	10.154	1.00	8.33	O
ATOM	1818	CB	THR	B	44	0.866	-0.665	8.700	1.00	10.78	C
ATOM	1819	OG1	THR	B	44	2.007	-0.343	7.897	1.00	14.62	O
ATOM	1820	CG2	THR	B	44	0.405	-2.051	8.339	1.00	9.81	C
ATOM	1828	N	ASP	B	45	0.702	2.596	8.166	1.00	7.76	N
ATOM	1829	CA	ASP	B	45	0.997	3.981	8.560	1.00	10.35	C
ATOM	1830	C	ASP	B	45	-0.282	4.741	8.899	1.00	15.38	C
ATOM	1831	O	ASP	B	45	-0.405	5.374	9.958	1.00	8.04	O
ATOM	1832	CB	ASP	B	45	1.721	4.658	7.401	1.00	15.97	C
ATOM	1833	CG	ASP	B	45	2.621	5.783	7.832	1.00	30.04	C
ATOM	1834	OD1	ASP	B	45	2.418	6.338	8.936	1.00	46.30	O
ATOM	1835	OD2	ASP	B	45	3.532	6.111	7.037	1.00	43.45	O
ATOM	1840	N	THR	B	46	-1.246	4.662	7.990	1.00	7.89	N
ATOM	1841	CA	THR	B	46	-2.557	5.281	8.147	1.00	7.82	C
ATOM	1842	C	THR	B	46	-3.284	4.690	9.342	1.00	11.43	C
ATOM	1843	O	THR	B	46	-3.837	5.425	10.176	1.00	7.48	O
ATOM	1844	CB	THR	B	46	-3.351	5.043	6.854	1.00	7.83	C
ATOM	1845	OG1	THR	B	46	-2.549	5.416	5.736	1.00	9.85	O
ATOM	1846	CG2	THR	B	46	-4.681	5.825	6.791	1.00	8.61	C
ATOM	1854	N	LEU	B	47	-3.217	3.368	9.474	1.00	7.18	N
ATOM	1855	CA	LEU	B	47	-3.850	2.697	10.588	1.00	6.88	C
ATOM	1856	C	LEU	B	47	-3.355	3.273	11.894	1.00	6.98	C
ATOM	1857	O	LEU	B	47	-4.148	3.554	12.795	1.00	6.89	O
ATOM	1858	CB	LEU	B	47	-3.578	1.190	10.518	1.00	6.67	C
ATOM	1859	CG	LEU	B	47	-4.062	0.319	11.689	1.00	6.44	C
ATOM	1860	CD1	LEU	B	47	-5.505	0.571	12.045	1.00	6.31	C
ATOM	1861	CD2	LEU	B	47	-3.933	-1.078	11.315	1.00	6.30	C
ATOM	1873	N	LEU	B	48	-2.033	3.453	12.011	1.00	7.21	N
ATOM	1874	CA	LEU	B	48	-1.469	4.071	13.208	1.00	9.76	C
ATOM	1875	C	LEU	B	48	-1.890	5.544	13.350	1.00	11.24	C
ATOM	1876	O	LEU	B	48	-2.180	6.004	14.461	1.00	7.68	O
ATOM	1877	CB	LEU	B	48	0.058	3.956	13.159	1.00	13.26	C
ATOM	1878	CG	LEU	B	48	0.862	4.594	14.312	1.00	16.06	C
ATOM	1879	CD1	LEU	B	48	0.652	3.755	15.525	1.00	8.10	C
ATOM	1880	CD2	LEU	B	48	2.368	4.774	14.050	1.00	8.48	C
ATOM	1892	N	LYS	B	49	-1.876	6.314	12.252	1.00	7.87	N
ATOM	1893	CA	LYS	B	49	-2.310	7.716	12.316	1.00	8.15	C
ATOM	1894	C	LYS	B	49	-3.747	7.819	12.824	1.00	10.28	C
ATOM	1895	O	LYS	B	49	-4.085	8.729	13.582	1.00	10.21	O
ATOM	1896	CB	LYS	B	49	-2.220	8.358	10.941	1.00	8.43	C
ATOM	1897	CG	LYS	B	49	-0.927	9.071	10.575	1.00	19.21	C
ATOM	1898	CD	LYS	B	49	0.351	8.294	10.853	1.00	19.16	C
ATOM	1899	CE	LYS	B	49	1.609	9.164	10.485	1.00	25.68	C
ATOM	1900	NZ	LYS	B	49	2.918	8.557	10.803	1.00	14.88	N
ATOM	1914	N	LEU	B	50	-4.621	6.914	12.371	1.00	13.53	N
ATOM	1915	C	LEU	B	50	-6.124	6.536	14.282	1.00	10.07	C
ATOM	1916	O	LEU	B	50	-6.837	7.185	15.054	1.00	14.20	O
ATOM	1933	CA	LEU	B	50	-6.010	6.952	12.815	1.00	7.38	C
ATOM	1934	CB	LEU	B	50	-6.886	6.082	11.900	1.00	10.58	C

ATOM	1935	CG	LEU	B	50	-7.224	6.553	10.461	1.00	10.98	C
ATOM	1936	CD1	LEU	B	50	-7.487	5.350	9.583	1.00	10.59	C
ATOM	1937	CD2	LEU	B	50	-8.458	7.483	10.369	1.00	10.19	C
ATOM	1949	N	SER	B	51	-5.422	5.474	14.697	1.00	7.05	N
ATOM	1950	CA	SER	B	51	-5.430	5.140	16.119	1.00	10.82	C
ATOM	1951	C	SER	B	51	-4.976	6.342	16.943	1.00	7.31	C
ATOM	1952	O	SER	B	51	-5.557	6.677	17.986	1.00	7.32	O
ATOM	1953	CB	SER	B	51	-4.524	3.946	16.362	1.00	12.41	C
ATOM	1954	OG	SER	B	51	-3.510	4.246	17.309	1.00	7.15	O
ATOM	1960	N	ASN	B	52	-3.986	7.044	16.421	1.00	11.15	N
ATOM	1961	CA	ASN	B	52	-3.436	8.217	17.075	1.00	13.28	C
ATOM	1962	C	ASN	B	52	-4.484	9.318	17.196	1.00	19.12	C
ATOM	1963	O	ASN	B	52	-4.609	9.947	18.260	1.00	11.57	O
ATOM	1964	CB	ASN	B	52	-2.184	8.665	16.288	1.00	8.37	C
ATOM	1965	CG	ASN	B	52	-0.949	7.792	16.594	1.00	8.42	C
ATOM	1966	OD1	ASN	B	52	-0.900	7.078	17.593	1.00	11.47	O
ATOM	1967	ND2	ASN	B	52	0.052	7.881	15.750	1.00	20.65	N
ATOM	1974	N	LEU	B	53	-5.262	9.548	16.122	1.00	8.04	N
ATOM	1975	CA	LEU	B	53	-6.217	10.650	16.122	1.00	8.21	C
ATOM	1976	C	LEU	B	53	-7.383	10.366	17.022	1.00	8.24	C
ATOM	1977	O	LEU	B	53	-7.930	11.298	17.630	1.00	12.84	O
ATOM	1978	CB	LEU	B	53	-6.748	10.950	14.714	1.00	16.09	C
ATOM	1979	CG	LEU	B	53	-7.651	12.209	14.576	1.00	8.56	C
ATOM	1980	CD1	LEU	B	53	-6.968	13.523	14.981	1.00	9.06	C
ATOM	1981	CD2	LEU	B	53	-8.173	12.325	13.189	1.00	8.85	C
ATOM	1993	N	TYR	B	54	-7.791	9.101	17.116	1.00	7.82	N
ATOM	1994	CA	TYR	B	54	-8.933	8.709	17.930	1.00	7.36	C
ATOM	1995	C	TYR	B	54	-8.571	8.259	19.343	1.00	7.33	C
ATOM	1996	O	TYR	B	54	-9.471	7.919	20.119	1.00	9.72	O
ATOM	1997	CB	TYR	B	54	-9.695	7.594	17.244	1.00	7.05	C
ATOM	1998	CG	TYR	B	54	-10.100	7.892	15.825	1.00	8.98	C
ATOM	1999	CD1	TYR	B	54	-10.432	9.152	15.453	1.00	7.41	C
ATOM	2000	CD2	TYR	B	54	-10.136	6.876	14.853	1.00	6.94	C
ATOM	2001	CE1	TYR	B	54	-10.815	9.446	14.158	1.00	12.98	C
ATOM	2002	CE2	TYR	B	54	-10.506	7.155	13.547	1.00	10.99	C
ATOM	2003	CZ	TYR	B	54	-10.850	8.441	13.213	1.00	7.37	C
ATOM	2004	OH	TYR	B	54	-11.202	8.726	11.913	1.00	14.41	O
ATOM	2014	N	ASN	B	55	-7.300	8.242	19.704	1.00	7.50	N
ATOM	2015	CA	ASN	B	55	-6.880	7.881	21.063	1.00	7.56	C
ATOM	2016	C	ASN	B	55	-7.172	6.429	21.442	1.00	7.26	C
ATOM	2017	O	ASN	B	55	-7.497	6.143	22.587	1.00	18.45	O
ATOM	2018	CB	ASN	B	55	-7.544	8.803	22.088	1.00	16.08	C
ATOM	2019	CG	ASN	B	55	-7.587	10.283	21.658	1.00	15.81	C
ATOM	2020	OD1	ASN	B	55	-6.576	10.873	21.324	1.00	13.16	O
ATOM	2021	ND2	ASN	B	55	-8.784	10.872	21.673	1.00	22.61	N
ATOM	2028	N	VAL	B	56	-7.044	5.498	20.495	1.00	10.99	N
ATOM	2029	CA	VAL	B	56	-7.260	4.070	20.721	1.00	6.80	C
ATOM	2030	C	VAL	B	56	-6.015	3.320	20.262	1.00	6.81	C
ATOM	2031	O	VAL	B	56	-5.158	3.851	19.555	1.00	7.46	O
ATOM	2032	CB	VAL	B	56	-8.517	3.485	19.999	1.00	6.53	C
ATOM	2033	CG1	VAL	B	56	-9.843	4.085	20.516	1.00	7.91	C
ATOM	2034	CG2	VAL	B	56	-8.415	3.644	18.531	1.00	6.48	C
ATOM	2044	N	SER	B	57	-5.906	2.080	20.715	1.00	6.72	N
ATOM	2045	CA	SER	B	57	-4.834	1.170	20.322	1.00	9.66	C
ATOM	2046	C	SER	B	57	-5.166	0.453	19.024	1.00	6.49	C
ATOM	2047	O	SER	B	57	-6.299	0.027	18.823	1.00	6.30	O
ATOM	2048	CB	SER	B	57	-4.614	0.129	21.412	1.00	7.46	C
ATOM	2049	OG	SER	B	57	-5.743	-0.692	21.608	1.00	6.61	O

```
ATOM   2055  N   THR B  58      -4.163   0.242  18.176  1.00  6.54           N
ATOM   2056  CA  THR B  58      -4.450  -0.494  16.944  1.00 14.90           C
ATOM   2057  C   THR B  58      -4.929  -1.920  17.236  1.00 12.79           C
ATOM   2058  O   THR B  58      -5.784  -2.444  16.510  1.00  6.04           O
ATOM   2059  CB  THR B  58      -3.217  -0.505  16.045  1.00 10.10           C
ATOM   2060  OG1 THR B  58      -2.179  -1.286  16.663  1.00  7.83           O
ATOM   2061  CG2 THR B  58      -2.745   0.950  15.811  1.00  6.70           C
ATOM   2069  N   ASP B  59      -4.407  -2.542  18.308  1.00  6.32           N
ATOM   2070  CA  ASP B  59      -4.975  -3.772  18.855  1.00 10.48           C
ATOM   2071  C   ASP B  59      -6.499  -3.703  19.037  1.00 17.11           C
ATOM   2072  O   ASP B  59      -7.198  -4.708  18.829  1.00  9.92           O
ATOM   2073  CB  ASP B  59      -4.305  -4.074  20.204  1.00  6.50           C
ATOM   2074  CG  ASP B  59      -2.886  -4.636  20.051  1.00  6.68           C
ATOM   2075  OD1 ASP B  59      -2.687  -5.630  19.352  1.00  6.62           O
ATOM   2076  OD2 ASP B  59      -1.968  -4.048  20.621  1.00 22.97           O
ATOM   2081  N   TYR B  60      -7.032  -2.549  19.480  1.00  6.14           N
ATOM   2082  CA  TYR B  60      -8.482  -2.437  19.605  1.00  6.05           C
ATOM   2083  C   TYR B  60      -9.142  -2.490  18.229  1.00 15.57           C
ATOM   2084  O   TYR B  60     -10.105  -3.235  18.011  1.00  5.89           O
ATOM   2085  CB  TYR B  60      -8.885  -1.140  20.331  1.00  6.74           C
ATOM   2086  CG  TYR B  60     -10.332  -0.742  20.049  1.00  6.06           C
ATOM   2087  CD1 TYR B  60     -11.415  -1.451  20.588  1.00 21.83           C
ATOM   2088  CD2 TYR B  60     -10.618   0.304  19.189  1.00  6.57           C
ATOM   2089  CE1 TYR B  60     -12.789  -1.088  20.284  1.00 13.43           C
ATOM   2090  CE2 TYR B  60     -11.978   0.667  18.871  1.00  6.03           C
ATOM   2091  CZ  TYR B  60     -13.038  -0.030  19.417  1.00  9.95           C
ATOM   2092  OH  TYR B  60     -14.329   0.331  19.085  1.00 15.78           O
ATOM   2102  N   LEU B  61      -8.645  -1.695  17.280  1.00  5.92           N
ATOM   2103  CA  LEU B  61      -9.320  -1.646  15.996  1.00 14.47           C
ATOM   2104  C   LEU B  61      -9.264  -3.007  15.313  1.00  5.98           C
ATOM   2105  O   LEU B  61     -10.175  -3.378  14.574  1.00  5.78           O
ATOM   2106  CB  LEU B  61      -8.706  -0.553  15.108  1.00  5.95           C
ATOM   2107  CG  LEU B  61      -8.658   0.853  15.680  1.00 10.84           C
ATOM   2108  CD1 LEU B  61      -7.769   1.761  14.836  1.00  6.22           C
ATOM   2109  CD2 LEU B  61     -10.111   1.406  15.798  1.00  6.06           C
ATOM   2121  N   LEU B  62      -8.228  -3.779  15.578  1.00 16.14           N
ATOM   2122  CA  LEU B  62      -8.051  -4.963  14.770  1.00  6.33           C
ATOM   2123  C   LEU B  62      -8.888  -6.113  15.260  1.00  5.81           C
ATOM   2124  O   LEU B  62      -9.077  -7.066  14.507  1.00 11.31           O
ATOM   2125  CB  LEU B  62      -6.574  -5.375  14.704  1.00  6.20           C
ATOM   2126  CG  LEU B  62      -5.675  -4.458  13.875  1.00  5.90           C
ATOM   2127  CD1 LEU B  62      -4.284  -5.044  13.776  1.00  8.02           C
ATOM   2128  CD2 LEU B  62      -6.239  -4.203  12.468  1.00  7.30           C
ATOM   2140  N   GLY B  63      -9.423  -6.047  16.479  1.00 10.72           N
ATOM   2141  CA  GLY B  63     -10.121  -7.217  16.975  1.00  9.22           C
ATOM   2142  C   GLY B  63      -9.193  -8.250  17.587  1.00  9.23           C
ATOM   2143  O   GLY B  63      -9.157  -9.379  17.099  1.00  6.92           O
ATOM   2147  N   LYS B  64      -8.422  -7.882  18.624  1.00 20.63           N
ATOM   2148  CA  LYS B  64      -7.495  -8.810  19.282  1.00  8.06           C
ATOM   2149  C   LYS B  64      -8.207  -9.695  20.299  1.00 37.72           C
ATOM   2150  O   LYS B  64      -7.599 -10.574  20.910  1.00 30.29           O
ATOM   2151  CB  LYS B  64      -6.387  -8.033  20.001  1.00 16.03           C
ATOM   2152  CG  LYS B  64      -5.223  -8.883  20.496  1.00 15.63           C
ATOM   2153  CD  LYS B  64      -4.037  -8.877  19.500  1.00 12.40           C
ATOM   2154  CE  LYS B  64      -2.729  -9.201  20.199  1.00 13.27           C
ATOM   2155  NZ  LYS B  64      -1.821 -10.252  19.581  1.00 17.72           N
ATOM    981  O5'  DA C   1     -16.802  28.342   1.182  1.00121.74           O
ATOM    982  C5'  DA C   1     -17.437  27.072   1.086  1.00133.28           C
```

```
ATOM    983  C4'  DA C   1     -18.342  26.813   2.279  1.00140.86           C
ATOM    984  O4'  DA C   1     -19.696  27.222   1.948  1.00128.59           O
ATOM    985  C3'  DA C   1     -18.444  25.352   2.714  1.00136.03           C
ATOM    986  O3'  DA C   1     -17.451  25.034   3.701  1.00148.84           O
ATOM    987  C2'  DA C   1     -19.865  25.282   3.263  1.00120.79           C
ATOM    988  C1'  DA C   1     -20.617  26.214   2.323  1.00118.27           C
ATOM    989  N9   DA C   1     -21.103  25.542   1.120  1.00109.33           N
ATOM    990  C8   DA C   1     -20.511  25.531  -0.111  1.00109.43           C
ATOM    991  N7   DA C   1     -21.169  24.840  -1.011  1.00105.97           N
ATOM    992  C5   DA C   1     -22.266  24.360  -0.321  1.00 95.48           C
ATOM    993  C6   DA C   1     -23.349  23.554  -0.719  1.00 96.35           C
ATOM    994  N6   DA C   1     -23.495  23.080  -1.960  1.00 89.18           N
ATOM    995  N1   DA C   1     -24.276  23.258   0.215  1.00 99.84           N
ATOM    996  C2   DA C   1     -24.123  23.736   1.455  1.00103.85           C
ATOM    997  N3   DA C   1     -23.148  24.501   1.945  1.00106.53           N
ATOM    998  C4   DA C   1     -22.242  24.780   0.995  1.00102.74           C
ATOM    999  P    DA C   2     -16.661  23.631   3.657  1.00149.55           P
ATOM   1000  OP1  DA C   2     -16.528  23.216   2.242  1.00125.52           O
ATOM   1001  OP2  DA C   2     -15.449  23.778   4.493  1.00119.97           O
ATOM   1002  O5'  DA C   2     -17.643  22.604   4.393  1.00128.22           O
ATOM   1003  C5'  DA C   2     -18.269  22.939   5.627  1.00129.22           C
ATOM   1004  C4'  DA C   2     -19.600  22.219   5.772  1.00124.39           C
ATOM   1005  O4'  DA C   2     -20.529  22.683   4.758  1.00126.06           O
ATOM   1006  C3'  DA C   2     -19.533  20.708   5.596  1.00116.50           C
ATOM   1007  O3'  DA C   2     -19.297  20.083   6.850  1.00123.01           O
ATOM   1008  C2'  DA C   2     -20.905  20.368   5.024  1.00104.19           C
ATOM   1009  C1'  DA C   2     -21.227  21.594   4.179  1.00111.31           C
ATOM   1010  N9   DA C   2     -20.832  21.472   2.775  1.00103.82           N
ATOM   1011  C8   DA C   2     -19.680  21.924   2.192  1.00104.62           C
ATOM   1012  N7   DA C   2     -19.595  21.676   0.908  1.00102.31           N
ATOM   1013  C5   DA C   2     -20.775  21.014   0.622  1.00 93.59           C
ATOM   1014  C6   DA C   2     -21.294  20.480  -0.574  1.00 94.91           C
ATOM   1015  N6   DA C   2     -20.651  20.540  -1.745  1.00 87.67           N
ATOM   1016  N1   DA C   2     -22.503  19.883  -0.514  1.00 90.74           N
ATOM   1017  C2   DA C   2     -23.137  19.828   0.664  1.00 97.37           C
ATOM   1018  N3   DA C   2     -22.750  20.293   1.850  1.00 96.38           N
ATOM   1019  C4   DA C   2     -21.549  20.881   1.760  1.00 90.57           C
ATOM   1020  P    DA C   3     -18.340  18.796   6.945  1.00113.96           P
ATOM   1021  OP1  DA C   3     -17.929  18.670   8.362  1.00111.52           O
ATOM   1022  OP2  DA C   3     -17.318  18.883   5.879  1.00 84.53           O
ATOM   1023  O5'  DA C   3     -19.298  17.576   6.566  1.00108.42           O
ATOM   1024  C5'  DA C   3     -20.495  17.349   7.297  1.00104.43           C
ATOM   1025  C4'  DA C   3     -21.490  16.567   6.456  1.00 92.07           C
ATOM   1026  O4'  DA C   3     -21.863  17.346   5.288  1.00 99.07           O
ATOM   1027  C3'  DA C   3     -20.960  15.252   5.892  1.00100.14           C
ATOM   1028  O3'  DA C   3     -21.066  14.195   6.852  1.00103.20           O
ATOM   1029  C2'  DA C   3     -21.862  15.059   4.676  1.00 97.24           C
ATOM   1030  C1'  DA C   3     -22.037  16.489   4.173  1.00 80.30           C
ATOM   1031  N9   DA C   3     -21.077  16.862   3.137  1.00 95.97           N
ATOM   1032  C8   DA C   3     -19.820  17.379   3.298  1.00105.42           C
ATOM   1033  N7   DA C   3     -19.196  17.615   2.167  1.00 93.47           N
ATOM   1034  C5   DA C   3     -20.108  17.226   1.200  1.00 86.86           C
ATOM   1035  C6   DA C   3     -20.060  17.222  -0.208  1.00 93.69           C
ATOM   1036  N6   DA C   3     -19.002  17.644  -0.911  1.00 99.43           N
ATOM   1037  N1   DA C   3     -21.148  16.766  -0.864  1.00 86.74           N
ATOM   1038  C2   DA C   3     -22.204  16.344  -0.153  1.00 88.55           C
ATOM   1039  N3   DA C   3     -22.364  16.300   1.165  1.00 86.63           N
ATOM   1040  C4   DA C   3     -21.269  16.759   1.783  1.00 90.96           C
```

```
ATOM   1041  P    DG C   4     -19.981  13.008   6.871  1.00 92.41           P
ATOM   1042  OP1  DG C   4     -20.116  12.299   8.161  1.00 87.47           O
ATOM   1043  OP2  DG C   4     -18.673  13.577   6.476  1.00 87.04           O
ATOM   1044  O5'  DG C   4     -20.464  12.015   5.710  1.00 96.10           O
ATOM   1045  C5'  DG C   4     -21.686  11.281   5.836  1.00 81.65           C
ATOM   1046  C4'  DG C   4     -22.122  10.779   4.472  1.00 88.14           C
ATOM   1047  O4'  DG C   4     -21.861  11.815   3.487  1.00 90.34           O
ATOM   1048  C3'  DG C   4     -21.356   9.537   4.020  1.00 93.71           C
ATOM   1049  O3'  DG C   4     -22.220   8.406   3.872  1.00 91.78           O
ATOM   1050  C2'  DG C   4     -20.711   9.927   2.693  1.00 85.41           C
ATOM   1051  C1'  DG C   4     -21.402  11.223   2.292  1.00 80.79           C
ATOM   1052  N9   DG C   4     -20.515  12.151   1.599  1.00 79.58           N
ATOM   1053  C8   DG C   4     -19.459  12.861   2.120  1.00 80.66           C
ATOM   1054  N7   DG C   4     -18.850  13.606   1.238  1.00 81.45           N
ATOM   1055  C5   DG C   4     -19.549  13.374   0.057  1.00 78.22           C
ATOM   1056  C6   DG C   4     -19.357  13.900  -1.244  1.00 76.88           C
ATOM   1057  O6   DG C   4     -18.500  14.708  -1.626  1.00 83.86           O
ATOM   1058  N1   DG C   4     -20.289  13.396  -2.152  1.00 76.68           N
ATOM   1059  C2   DG C   4     -21.283  12.499  -1.841  1.00 81.56           C
ATOM   1060  N2   DG C   4     -22.095  12.121  -2.843  1.00 82.66           N
ATOM   1061  N3   DG C   4     -21.471  12.003  -0.628  1.00 86.43           N
ATOM   1062  C4   DG C   4     -20.573  12.480   0.265  1.00 80.54           C
ATOM   1063  P    DT C   5     -21.614   6.966   3.492  1.00 73.32           P
ATOM   1064  OP1  DT C   5     -20.167   6.974   3.805  1.00 66.76           O
ATOM   1065  OP2  DT C   5     -22.505   5.945   4.085  1.00 71.55           O
ATOM   1066  O5'  DT C   5     -21.782   6.890   1.900  1.00 73.66           O
ATOM   1067  C5'  DT C   5     -23.059   7.071   1.315  1.00 81.60           C
ATOM   1068  C4'  DT C   5     -22.961   7.227  -0.190  1.00 73.03           C
ATOM   1069  O4'  DT C   5     -22.140   8.375  -0.520  1.00 68.14           O
ATOM   1070  C3'  DT C   5     -22.314   6.046  -0.902  1.00 87.71           C
ATOM   1071  O3'  DT C   5     -23.333   5.184  -1.407  1.00 94.91           O
ATOM   1072  C2'  DT C   5     -21.449   6.671  -2.001  1.00 74.48           C
ATOM   1073  C1'  DT C   5     -21.591   8.179  -1.807  1.00 74.75           C
ATOM   1074  N1   DT C   5     -20.309   8.938  -1.892  1.00 68.72           N
ATOM   1075  C2   DT C   5     -19.843   9.345  -3.124  1.00 82.39           C
ATOM   1076  O2   DT C   5     -20.422   9.112  -4.170  1.00 95.21           O
ATOM   1077  N3   DT C   5     -18.659  10.039  -3.089  1.00 81.60           N
ATOM   1078  C4   DT C   5     -17.917  10.361  -1.968  1.00 77.38           C
ATOM   1079  O4   DT C   5     -16.865  10.988  -2.039  1.00 73.71           O
ATOM   1080  C5   DT C   5     -18.464   9.908  -0.715  1.00 68.39           C
ATOM   1081  C7   DT C   5     -17.752  10.194   0.572  1.00 60.32           C
ATOM   1082  C6   DT C   5     -19.617   9.229  -0.734  1.00 77.95           C
ATOM   1083  P    DT C   6     -22.944   3.902  -2.293  1.00105.93           P
ATOM   1084  OP1  DT C   6     -21.582   3.478  -1.904  1.00 81.08           O
ATOM   1085  OP2  DT C   6     -24.063   2.938  -2.218  1.00 94.38           O
ATOM   1086  O5'  DT C   6     -22.891   4.481  -3.779  1.00 87.74           O
ATOM   1087  C5'  DT C   6     -22.197   3.766  -4.778  1.00 91.35           C
ATOM   1088  C4'  DT C   6     -21.605   4.727  -5.787  1.00 91.92           C
ATOM   1089  O4'  DT C   6     -20.830   5.743  -5.109  1.00 90.35           O
ATOM   1090  C3'  DT C   6     -20.651   4.074  -6.775  1.00102.82           C
ATOM   1091  O3'  DT C   6     -21.365   3.778  -7.969  1.00 99.94           O
ATOM   1092  C2'  DT C   6     -19.528   5.095  -6.974  1.00101.97           C
ATOM   1093  C1'  DT C   6     -19.823   6.217  -5.981  1.00 88.79           C
ATOM   1094  N1   DT C   6     -18.642   6.650  -5.165  1.00 82.02           N
ATOM   1095  C2   DT C   6     -17.716   7.512  -5.715  1.00 83.12           C
ATOM   1096  O2   DT C   6     -17.793   7.947  -6.849  1.00 80.88           O
ATOM   1097  N3   DT C   6     -16.679   7.848  -4.881  1.00 92.10           N
ATOM   1098  C4   DT C   6     -16.481   7.422  -3.582  1.00 77.89           C
```

```
ATOM   1099  O4   DT C   6     -15.516   7.782  -2.913  1.00 67.06           O
ATOM   1100  C5   DT C   6     -17.486   6.527  -3.068  1.00 72.17           C
ATOM   1101  C7   DT C   6     -17.380   5.995  -1.670  1.00 79.37           C
ATOM   1102  C6   DT C   6     -18.503   6.188  -3.870  1.00 88.66           C
ATOM   1103  P    DC C   7     -20.598   3.186  -9.248  1.00104.76           P
ATOM   1104  OP1  DC C   7     -21.612   2.540 -10.110  1.00111.84           O
ATOM   1105  OP2  DC C   7     -19.432   2.411  -8.765  1.00107.32           O
ATOM   1106  O5'  DC C   7     -20.044   4.488  -9.989  1.00 88.63           O
ATOM   1107  C5'  DC C   7     -19.029   4.367 -10.966  1.00105.05           C
ATOM   1108  C4'  DC C   7     -18.338   5.701 -11.163  1.00 98.37           C
ATOM   1109  O4'  DC C   7     -17.995   6.257  -9.873  1.00101.77           O
ATOM   1110  C3'  DC C   7     -17.038   5.621 -11.944  1.00106.13           C
ATOM   1111  O3'  DC C   7     -17.303   5.920 -13.304  1.00118.36           O
ATOM   1112  C2'  DC C   7     -16.117   6.649 -11.288  1.00 91.93           C
ATOM   1113  C1'  DC C   7     -16.756   6.935  -9.933  1.00 95.95           C
ATOM   1114  N1   DC C   7     -15.941   6.504  -8.758  1.00 88.39           N
ATOM   1115  C2   DC C   7     -14.810   7.243  -8.397  1.00 94.16           C
ATOM   1116  O2   DC C   7     -14.496   8.232  -9.070  1.00 92.03           O
ATOM   1117  N3   DC C   7     -14.083   6.849  -7.322  1.00 94.31           N
ATOM   1118  C4   DC C   7     -14.452   5.774  -6.624  1.00 92.22           C
ATOM   1119  N4   DC C   7     -13.708   5.426  -5.571  1.00 90.76           N
ATOM   1120  C5   DC C   7     -15.606   5.011  -6.971  1.00 94.69           C
ATOM   1121  C6   DC C   7     -16.314   5.408  -8.034  1.00 94.05           C
ATOM   1122  P    DT C   8     -16.547   5.127 -14.501  1.00122.47           P
ATOM   1123  OP1  DT C   8     -17.081   5.633 -15.790  1.00130.95           O
ATOM   1124  OP2  DT C   8     -16.595   3.666 -14.215  1.00112.09           O
ATOM   1125  O5'  DT C   8     -15.036   5.624 -14.385  1.00 92.77           O
ATOM   1126  C5'  DT C   8     -14.670   6.967 -14.754  1.00103.97           C
ATOM   1127  C4'  DT C   8     -13.202   7.216 -14.496  1.00108.66           C
ATOM   1128  O4'  DT C   8     -12.929   7.129 -13.076  1.00119.64           O
ATOM   1129  C3'  DT C   8     -12.248   6.231 -15.181  1.00118.91           C
ATOM   1130  O3'  DT C   8     -11.219   6.954 -15.866  1.00144.84           O
ATOM   1131  C2'  DT C   8     -11.665   5.424 -14.034  1.00108.98           C
ATOM   1132  C1'  DT C   8     -11.733   6.399 -12.878  1.00108.32           C
ATOM   1133  N1   DT C   8     -11.804   5.761 -11.552  1.00 96.89           N
ATOM   1134  C2   DT C   8     -10.956   6.211 -10.566  1.00100.67           C
ATOM   1135  O2   DT C   8     -10.153   7.114 -10.738  1.00 96.87           O
ATOM   1136  N3   DT C   8     -11.083   5.563  -9.362  1.00101.19           N
ATOM   1137  C4   DT C   8     -11.950   4.531  -9.057  1.00102.62           C
ATOM   1138  O4   DT C   8     -11.948   4.044  -7.930  1.00101.87           O
ATOM   1139  C5   DT C   8     -12.807   4.106 -10.140  1.00100.87           C
ATOM   1140  C7   DT C   8     -13.781   2.995  -9.904  1.00105.82           C
ATOM   1141  C6   DT C   8     -12.691   4.733 -11.318  1.00 97.68           C
ATOM   1142  P    DC C   9     -10.246   6.205 -16.899  1.00133.24           P
ATOM   1143  OP1  DC C   9     -10.152   7.039 -18.124  1.00132.94           O
ATOM   1144  OP2  DC C   9     -10.679   4.788 -17.010  1.00 73.87           O
ATOM   1145  O5'  DC C   9      -8.835   6.257 -16.162  1.00119.88           O
ATOM   1146  C5'  DC C   9      -8.323   7.503 -15.651  1.00121.43           C
ATOM   1147  C4'  DC C   9      -7.403   7.259 -14.477  1.00115.61           C
ATOM   1148  O4'  DC C   9      -8.115   6.565 -13.431  1.00114.65           O
ATOM   1149  C3'  DC C   9      -6.172   6.402 -14.788  1.00116.90           C
ATOM   1150  O3'  DC C   9      -5.003   7.230 -14.800  1.00121.05           O
ATOM   1151  C2'  DC C   9      -6.099   5.405 -13.639  1.00112.09           C
ATOM   1152  C1'  DC C   9      -7.166   5.860 -12.660  1.00112.03           C
ATOM   1153  N1   DC C   9      -7.861   4.760 -11.977  1.00103.11           N
ATOM   1154  C2   DC C   9      -7.616   4.558 -10.618  1.00 95.66           C
ATOM   1155  O2   DC C   9      -6.831   5.318 -10.033  1.00103.63           O
ATOM   1156  N3   DC C   9      -8.243   3.547  -9.975  1.00 90.79           N
```

```
ATOM   1157  C4   DC C   9      -9.083   2.753 -10.641  1.00 93.31           C
ATOM   1158  N4   DC C   9      -9.681   1.769  -9.966  1.00 87.93           N
ATOM   1159  C5   DC C   9      -9.349   2.934 -12.030  1.00 94.96           C
ATOM   1160  C6   DC C   9      -8.718   3.937 -12.654  1.00103.80           C
ATOM   1161  P    DT C  10      -3.563   6.606 -15.141  1.00109.09           P
ATOM   1162  OP1  DT C  10      -2.748   7.665 -15.788  1.00123.53           O
ATOM   1163  OP2  DT C  10      -3.767   5.311 -15.840  1.00 90.57           O
ATOM   1164  O5'  DT C  10      -2.939   6.326 -13.702  1.00 99.93           O
ATOM   1165  C5'  DT C  10      -2.891   7.363 -12.703  1.00101.08           C
ATOM   1166  C4'  DT C  10      -2.292   6.840 -11.417  1.00104.85           C
ATOM   1167  O4'  DT C  10      -3.193   5.880 -10.816  1.00112.44           O
ATOM   1168  C3'  DT C  10      -0.943   6.131 -11.577  1.00114.67           C
ATOM   1169  O3'  DT C  10       0.030   6.698 -10.680  1.00115.28           O
ATOM   1170  C2'  DT C  10      -1.241   4.676 -11.247  1.00108.85           C
ATOM   1171  C1'  DT C  10      -2.458   4.766 -10.349  1.00105.53           C
ATOM   1172  N1   DT C  10      -3.337   3.586 -10.412  1.00 97.66           N
ATOM   1173  C2   DT C  10      -3.547   2.869  -9.256  1.00 99.23           C
ATOM   1174  O2   DT C  10      -3.044   3.168  -8.185  1.00 90.05           O
ATOM   1175  N3   DT C  10      -4.376   1.785  -9.399  1.00 99.11           N
ATOM   1176  C4   DT C  10      -5.002   1.358 -10.554  1.00 97.79           C
ATOM   1177  O4   DT C  10      -5.721   0.363 -10.529  1.00102.88           O
ATOM   1178  C5   DT C  10      -4.735   2.156 -11.729  1.00 95.77           C
ATOM   1179  C7   DT C  10      -5.365   1.767 -13.029  1.00102.89           C
ATOM   1180  C6   DT C  10      -3.925   3.214 -11.601  1.00 88.79           C
ATOM   1181  P    DT C  11       1.580   6.183 -10.647  1.00124.49           P
ATOM   1182  OP1  DT C  11       2.425   7.355 -10.325  1.00131.90           O
ATOM   1183  OP2  DT C  11       1.838   5.394 -11.873  1.00111.94           O
ATOM   1184  O5'  DT C  11       1.601   5.175  -9.404  1.00127.37           O
ATOM   1185  C5'  DT C  11       1.238   5.655  -8.115  1.00123.95           C
ATOM   1186  C4'  DT C  11       1.052   4.519  -7.124  1.00105.37           C
ATOM   1187  O4'  DT C  11       0.112   3.548  -7.645  1.00104.31           O
ATOM   1188  C3'  DT C  11       2.322   3.734  -6.802  1.00105.96           C
ATOM   1189  O3'  DT C  11       2.754   4.063  -5.483  1.00104.34           O
ATOM   1190  C2'  DT C  11       1.922   2.265  -6.949  1.00 98.14           C
ATOM   1191  C1'  DT C  11       0.401   2.318  -7.024  1.00 95.50           C
ATOM   1192  N1   DT C  11      -0.217   1.214  -7.809  1.00 93.78           N
ATOM   1193  C2   DT C  11      -0.758   0.144  -7.136  1.00 91.82           C
ATOM   1194  O2   DT C  11      -0.761   0.050  -5.923  1.00102.27           O
ATOM   1195  N3   DT C  11      -1.302  -0.822  -7.940  1.00 94.04           N
ATOM   1196  C4   DT C  11      -1.355  -0.821  -9.320  1.00101.78           C
ATOM   1197  O4   DT C  11      -1.867  -1.740  -9.950  1.00110.63           O
ATOM   1198  C5   DT C  11      -0.770   0.329  -9.961  1.00 90.95           C
ATOM   1199  C7   DT C  11      -0.771   0.439 -11.457  1.00 92.75           C
ATOM   1200  C6   DT C  11      -0.235   1.281  -9.187  1.00 91.15           C
ATOM   1201  P    DT C  12       4.178   3.556  -4.938  1.00100.88           P
ATOM   1202  OP1  DT C  12       4.683   4.585  -4.000  1.00 66.72           O
ATOM   1203  OP2  DT C  12       4.998   3.133  -6.096  1.00103.89           O
ATOM   1204  O5'  DT C  12       3.820   2.242  -4.106  1.00101.70           O
ATOM   1205  C5'  DT C  12       2.827   2.318  -3.100  1.00 86.37           C
ATOM   1206  C4'  DT C  12       2.161   0.968  -2.930  1.00 89.87           C
ATOM   1207  O4'  DT C  12       1.723   0.476  -4.215  1.00 90.14           O
ATOM   1208  C3'  DT C  12       3.060  -0.131  -2.383  1.00 84.42           C
ATOM   1209  O3'  DT C  12       2.856  -0.224  -0.976  1.00 86.65           O
ATOM   1210  C2'  DT C  12       2.634  -1.390  -3.145  1.00 82.64           C
ATOM   1211  C1'  DT C  12       1.525  -0.913  -4.079  1.00 83.58           C
ATOM   1212  N1   DT C  12       1.509  -1.520  -5.445  1.00 87.12           N
ATOM   1213  C2   DT C  12       0.555  -2.468  -5.744  1.00 94.02           C
ATOM   1214  O2   DT C  12      -0.282  -2.851  -4.947  1.00 98.51           O
```

```
ATOM   1215  N3   DT C  12       0.614  -2.959  -7.024  1.00 92.68           N
ATOM   1216  C4   DT C  12       1.511  -2.601  -8.010  1.00 91.91           C
ATOM   1217  O4   DT C  12       1.480  -3.102  -9.128  1.00 99.85           O
ATOM   1218  C5   DT C  12       2.478  -1.603  -7.634  1.00 91.25           C
ATOM   1219  C7   DT C  12       3.501  -1.142  -8.629  1.00 92.91           C
ATOM   1220  C6   DT C  12       2.433  -1.113  -6.386  1.00 91.95           C
ATOM   1221  P    DA C  13       3.798  -1.149  -0.029  1.00 97.83           P
ATOM   1222  OP1  DA C  13       3.719  -0.604   1.347  1.00 87.77           O
ATOM   1223  OP2  DA C  13       5.135  -1.308  -0.669  1.00 89.36           O
ATOM   1224  O5'  DA C  13       3.058  -2.562  -0.042  1.00 81.82           O
ATOM   1225  C5'  DA C  13       1.622  -2.656   0.074  1.00 73.49           C
ATOM   1226  C4'  DA C  13       1.102  -3.930  -0.558  1.00 70.26           C
ATOM   1227  O4'  DA C  13       1.360  -3.944  -1.985  1.00 77.31           O
ATOM   1228  C3'  DA C  13       1.712  -5.222  -0.017  1.00 65.97           C
ATOM   1229  O3'  DA C  13       0.711  -6.233  -0.031  1.00 66.97           O
ATOM   1230  C2'  DA C  13       2.718  -5.603  -1.081  1.00 73.15           C
ATOM   1231  C1'  DA C  13       1.989  -5.168  -2.332  1.00 82.73           C
ATOM   1232  N9   DA C  13       2.848  -4.922  -3.488  1.00 95.97           N
ATOM   1233  C8   DA C  13       4.082  -4.321  -3.515  1.00 86.54           C
ATOM   1234  N7   DA C  13       4.603  -4.238  -4.714  1.00 87.70           N
ATOM   1235  C5   DA C  13       3.652  -4.831  -5.532  1.00 85.88           C
ATOM   1236  C6   DA C  13       3.609  -5.063  -6.918  1.00 89.24           C
ATOM   1237  N6   DA C  13       4.582  -4.708  -7.759  1.00 87.90           N
ATOM   1238  N1   DA C  13       2.517  -5.682  -7.416  1.00 86.79           N
ATOM   1239  C2   DA C  13       1.541  -6.039  -6.572  1.00 90.34           C
ATOM   1240  N3   DA C  13       1.467  -5.876  -5.253  1.00 81.20           N
ATOM   1241  C4   DA C  13       2.568  -5.261  -4.789  1.00 83.58           C
ATOM   1242  P    DG C  14       0.155  -6.838   1.328  1.00 71.20           P
ATOM   1243  OP1  DG C  14      -0.662  -5.793   1.991  1.00 72.18           O
ATOM   1244  OP2  DG C  14       1.291  -7.458   2.051  1.00 72.15           O
ATOM   1245  O5'  DG C  14      -0.801  -8.005   0.818  1.00 57.39           O
ATOM   1246  C5'  DG C  14      -1.145  -8.110  -0.578  1.00 64.08           C
ATOM   1247  C4'  DG C  14      -0.359  -9.216  -1.245  1.00 72.51           C
ATOM   1248  O4'  DG C  14       0.675  -8.670  -2.096  1.00 75.94           O
ATOM   1249  C3'  DG C  14       0.346 -10.192  -0.295  1.00 75.64           C
ATOM   1250  O3'  DG C  14      -0.258 -11.474  -0.461  1.00 71.26           O
ATOM   1251  C2'  DG C  14       1.785 -10.224  -0.786  1.00 62.61           C
ATOM   1252  C1'  DG C  14       1.689  -9.647  -2.182  1.00 69.33           C
ATOM   1253  N9   DG C  14       2.908  -9.001  -2.657  1.00 64.14           N
ATOM   1254  C8   DG C  14       3.917  -8.466  -1.894  1.00 78.56           C
ATOM   1255  N7   DG C  14       4.892  -7.972  -2.608  1.00 85.03           N
ATOM   1256  C5   DG C  14       4.516  -8.221  -3.920  1.00 78.20           C
ATOM   1257  C6   DG C  14       5.179  -7.922  -5.139  1.00 80.17           C
ATOM   1258  O6   DG C  14       6.266  -7.356  -5.307  1.00 89.53           O
ATOM   1259  N1   DG C  14       4.445  -8.354  -6.239  1.00 79.79           N
ATOM   1260  C2   DG C  14       3.230  -8.990  -6.177  1.00 88.02           C
ATOM   1261  N2   DG C  14       2.678  -9.325  -7.350  1.00 93.26           N
ATOM   1262  N3   DG C  14       2.602  -9.275  -5.048  1.00 85.43           N
ATOM   1263  C4   DG C  14       3.298  -8.864  -3.967  1.00 74.64           C
ATOM   1264  P    DA C  15      -0.044 -12.641   0.617  1.00 69.73           P
ATOM   1265  OP1  DA C  15      -1.134 -12.546   1.619  1.00 72.44           O
ATOM   1266  OP2  DA C  15       1.371 -12.625   1.066  1.00 76.74           O
ATOM   1267  O5'  DA C  15      -0.278 -13.947  -0.262  1.00 97.88           O
ATOM   1268  C5'  DA C  15      -1.228 -13.917  -1.344  1.00 96.14           C
ATOM   1269  C4'  DA C  15      -0.561 -14.226  -2.665  1.00 79.71           C
ATOM   1270  O4'  DA C  15       0.455 -13.249  -2.978  1.00 84.12           O
ATOM   1271  C3'  DA C  15       0.141 -15.584  -2.727  1.00 95.17           C
ATOM   1272  O3'  DA C  15      -0.683 -16.487  -3.478  1.00113.14           O
```

```
ATOM   1273  C2'  DA C  15       1.451 -15.304  -3.449  1.00 89.17           C
ATOM   1274  C1'  DA C  15       1.340 -13.857  -3.896  1.00 83.40           C
ATOM   1275  N9   DA C  15       2.603 -13.124  -3.863  1.00 80.12           N
ATOM   1276  C8   DA C  15       3.310 -12.700  -2.765  1.00 85.34           C
ATOM   1277  N7   DA C  15       4.422 -12.072  -3.062  1.00 79.93           N
ATOM   1278  C5   DA C  15       4.457 -12.095  -4.449  1.00 76.17           C
ATOM   1279  C6   DA C  15       5.382 -11.594  -5.380  1.00 84.71           C
ATOM   1280  N6   DA C  15       6.499 -10.947  -5.040  1.00 97.47           N
ATOM   1281  N1   DA C  15       5.120 -11.787  -6.691  1.00 75.88           N
ATOM   1282  C2   DA C  15       4.001 -12.437  -7.032  1.00 78.16           C
ATOM   1283  N3   DA C  15       3.054 -12.951  -6.251  1.00 73.08           N
ATOM   1284  C4   DA C  15       3.345 -12.744  -4.955  1.00 71.69           C
ATOM   1285  P    DG C  16      -0.354 -18.073  -3.565  1.00122.43           P
ATOM   1286  OP1  DG C  16      -1.576 -18.745  -4.061  1.00 95.38           O
ATOM   1287  OP2  DG C  16       0.254 -18.497  -2.283  1.00104.99           O
ATOM   1288  O5'  DG C  16       0.785 -18.162  -4.683  1.00103.09           O
ATOM   1289  C5'  DG C  16       0.538 -17.689  -6.002  1.00101.88           C
ATOM   1290  C4'  DG C  16       1.774 -17.873  -6.864  1.00104.91           C
ATOM   1291  O4'  DG C  16       2.712 -16.783  -6.643  1.00 94.28           O
ATOM   1292  C3'  DG C  16       2.546 -19.161  -6.575  1.00119.61           C
ATOM   1293  O3'  DG C  16       2.901 -19.830  -7.789  1.00131.66           O
ATOM   1294  C2'  DG C  16       3.764 -18.658  -5.804  1.00103.35           C
ATOM   1295  C1'  DG C  16       4.010 -17.330  -6.504  1.00 90.65           C
ATOM   1296  N9   DG C  16       4.881 -16.401  -5.781  1.00 90.55           N
ATOM   1297  C8   DG C  16       4.849 -16.083  -4.444  1.00 88.13           C
ATOM   1298  N7   DG C  16       5.762 -15.216  -4.094  1.00 75.50           N
ATOM   1299  C5   DG C  16       6.443 -14.942  -5.272  1.00 76.72           C
ATOM   1300  C6   DG C  16       7.534 -14.074  -5.519  1.00 90.59           C
ATOM   1301  O6   DG C  16       8.138 -13.351  -4.714  1.00 92.77           O
ATOM   1302  N1   DG C  16       7.920 -14.093  -6.857  1.00 90.44           N
ATOM   1303  C2   DG C  16       7.325 -14.853  -7.836  1.00 96.50           C
ATOM   1304  N2   DG C  16       7.834 -14.742  -9.073  1.00 98.73           N
ATOM   1305  N3   DG C  16       6.300 -15.669  -7.618  1.00 90.40           N
ATOM   1306  C4   DG C  16       5.913 -15.662  -6.319  1.00 83.88           C
ATOM   1307  P    DA C  17       2.918 -21.437  -7.855  1.00113.74           P
ATOM   1308  OP1  DA C  17       3.098 -21.823  -9.272  1.00121.75           O
ATOM   1309  OP2  DA C  17       1.743 -21.935  -7.105  1.00112.58           O
ATOM   1310  O5'  DA C  17       4.243 -21.843  -7.055  1.00112.33           O
ATOM   1311  C5'  DA C  17       5.475 -22.068  -7.747  1.00123.63           C
ATOM   1312  C4'  DA C  17       6.006 -20.815  -8.425  1.00111.25           C
ATOM   1313  O4'  DA C  17       6.173 -19.759  -7.451  1.00111.54           O
ATOM   1314  C3'  DA C  17       7.382 -20.974  -9.064  1.00120.55           C
ATOM   1315  O3'  DA C  17       7.241 -21.353 -10.435  1.00138.99           O
ATOM   1316  C2'  DA C  17       8.048 -19.606  -8.894  1.00 97.64           C
ATOM   1317  C1'  DA C  17       7.180 -18.865  -7.880  1.00 96.99           C
ATOM   1318  N9   DA C  17       7.886 -18.385  -6.691  1.00 93.25           N
ATOM   1319  C8   DA C  17       7.593 -18.688  -5.389  1.00 94.99           C
ATOM   1320  N7   DA C  17       8.383 -18.113  -4.515  1.00 87.43           N
ATOM   1321  C5   DA C  17       9.258 -17.378  -5.295  1.00 91.58           C
ATOM   1322  C6   DA C  17      10.341 -16.542  -4.963  1.00 97.95           C
ATOM   1323  N6   DA C  17      10.725 -16.308  -3.704  1.00 88.99           N
ATOM   1324  N1   DA C  17      11.008 -15.955  -5.979  1.00104.17           N
ATOM   1325  C2   DA C  17      10.610 -16.197  -7.235  1.00104.85           C
ATOM   1326  N3   DA C  17       9.610 -16.964  -7.669  1.00 94.12           N
ATOM   1327  C4   DA C  17       8.965 -17.532  -6.638  1.00 92.62           C
ATOM   1328  P    DA C  18       8.382 -22.224 -11.160  1.00141.38           P
ATOM   1329  OP1  DA C  18       7.741 -22.943 -12.284  1.00124.15           O
ATOM   1330  OP2  DA C  18       9.106 -22.988 -10.119  1.00119.12           O
```

```
ATOM   1331  O5'  DA C  18       9.379 -21.125 -11.756  1.00111.14           O
ATOM   1332  C5'  DA C  18      10.779 -21.367 -11.809  1.00105.91           C
ATOM   1333  C4'  DA C  18      11.532 -20.167 -11.264  1.00105.65           C
ATOM   1334  O4'  DA C  18      10.917 -19.740 -10.025  1.00107.19           O
ATOM   1335  C3'  DA C  18      12.992 -20.420 -10.917  1.00112.75           C
ATOM   1336  O3'  DA C  18      13.826 -20.180 -12.046  1.00120.29           O
ATOM   1337  C2'  DA C  18      13.261 -19.435  -9.782  1.00116.61           C
ATOM   1338  C1'  DA C  18      11.885 -19.143  -9.185  1.00118.73           C
ATOM   1339  N9   DA C  18      11.711 -19.653  -7.823  1.00113.91           N
ATOM   1340  C8   DA C  18      10.972 -20.733  -7.425  1.00110.30           C
ATOM   1341  N7   DA C  18      10.999 -20.948  -6.131  1.00104.85           N
ATOM   1342  C5   DA C  18      11.815 -19.939  -5.645  1.00102.42           C
ATOM   1343  C6   DA C  18      12.245 -19.613  -4.345  1.00107.12           C
ATOM   1344  N6   DA C  18      11.892 -20.306  -3.257  1.00 91.05           N
ATOM   1345  N1   DA C  18      13.056 -18.542  -4.207  1.00110.02           N
ATOM   1346  C2   DA C  18      13.405 -17.854  -5.300  1.00106.04           C
ATOM   1347  N3   DA C  18      13.065 -18.064  -6.570  1.00103.26           N
ATOM   1348  C4   DA C  18      12.261 -19.133  -6.674  1.00102.41           C
ATOM   1349  P    DC C  19      15.173 -21.032 -12.256  1.00125.16           P
ATOM   1350  OP1  DC C  19      15.626 -20.811 -13.646  1.00136.10           O
ATOM   1351  OP2  DC C  19      14.915 -22.410 -11.779  1.00115.64           O
ATOM   1352  O5'  DC C  19      16.224 -20.348 -11.262  1.00105.72           O
ATOM   1353  C5'  DC C  19      16.772 -19.077 -11.588  1.00103.85           C
ATOM   1354  C4'  DC C  19      17.194 -18.335 -10.334  1.00112.66           C
ATOM   1355  O4'  DC C  19      16.161 -18.490  -9.331  1.00125.86           O
ATOM   1356  C3'  DC C  19      18.460 -18.849  -9.663  1.00121.15           C
ATOM   1357  O3'  DC C  19      19.614 -18.224 -10.214  1.00119.94           O
ATOM   1358  C2'  DC C  19      18.249 -18.470  -8.201  1.00128.24           C
ATOM   1359  C1'  DC C  19      16.732 -18.438  -8.036  1.00128.70           C
ATOM   1360  N1   DC C  19      16.197 -19.567  -7.209  1.00126.02           N
ATOM   1361  C2   DC C  19      16.100 -19.412  -5.821  1.00121.71           C
ATOM   1362  O2   DC C  19      16.460 -18.344  -5.312  1.00118.99           O
ATOM   1363  N3   DC C  19      15.616 -20.435  -5.072  1.00108.10           N
ATOM   1364  C4   DC C  19      15.239 -21.571  -5.661  1.00116.11           C
ATOM   1365  N4   DC C  19      14.767 -22.552  -4.883  1.00114.30           N
ATOM   1366  C5   DC C  19      15.330 -21.749  -7.073  1.00114.38           C
ATOM   1367  C6   DC C  19      15.810 -20.734  -7.801  1.00111.87           C
ATOM   1368  P    DA C  20      21.025 -18.991 -10.213  1.00127.50           P
ATOM   1369  OP1  DA C  20      21.961 -18.195 -11.040  1.00118.95           O
ATOM   1370  OP2  DA C  20      20.766 -20.410 -10.548  1.00120.81           O
ATOM   1371  O5'  DA C  20      21.500 -18.931  -8.685  1.00119.65           O
ATOM   1372  C5'  DA C  20      21.869 -17.685  -8.109  1.00109.56           C
ATOM   1373  C4'  DA C  20      21.612 -17.663  -6.612  1.00116.50           C
ATOM   1374  O4'  DA C  20      20.531 -18.580  -6.282  1.00118.34           O
ATOM   1375  C3'  DA C  20      22.777 -18.099  -5.731  1.00125.61           C
ATOM   1376  O3'  DA C  20      22.787 -17.321  -4.541  1.00136.75           O
ATOM   1377  C2'  DA C  20      22.450 -19.557  -5.445  1.00125.12           C
ATOM   1378  C1'  DA C  20      20.955 -19.415  -5.222  1.00119.91           C
ATOM   1379  N9   DA C  20      20.210 -20.667  -5.244  1.00117.86           N
ATOM   1380  C8   DA C  20      20.322 -21.698  -6.134  1.00123.30           C
ATOM   1381  N7   DA C  20      19.506 -22.698  -5.888  1.00121.89           N
ATOM   1382  C5   DA C  20      18.811 -22.290  -4.760  1.00115.32           C
ATOM   1383  C6   DA C  20      17.796 -22.896  -3.993  1.00111.14           C
ATOM   1384  N6   DA C  20      17.284 -24.101  -4.264  1.00112.39           N
ATOM   1385  N1   DA C  20      17.326 -22.209  -2.930  1.00101.28           N
ATOM   1386  C2   DA C  20      17.840 -21.003  -2.661  1.00104.07           C
ATOM   1387  N3   DA C  20      18.791 -20.334  -3.305  1.00108.46           N
ATOM   1388  C4   DA C  20      19.235 -21.040  -4.354  1.00111.97           C
```

ATOM	1389	P	DA	C	21	23.754	-17.716	-3.319	1.00161.14	P
ATOM	1390	OP1	DA	C	21	23.957	-16.495	-2.507	1.00163.21	O
ATOM	1391	OP2	DA	C	21	24.919	-18.427	-3.893	1.00143.11	O
ATOM	1392	O5'	DA	C	21	22.900	-18.768	-2.464	1.00139.44	O
ATOM	1393	C5'	DA	C	21	22.618	-18.516	-1.084	1.00144.68	C
ATOM	1394	C4'	DA	C	21	23.660	-19.120	-0.154	1.00136.50	C
ATOM	1395	O4'	DA	C	21	23.351	-20.521	0.050	1.00117.80	O
ATOM	1396	C3'	DA	C	21	25.102	-19.103	-0.648	1.00137.61	C
ATOM	1397	O3'	DA	C	21	26.022	-19.076	0.442	1.00117.34	O
ATOM	1398	C2'	DA	C	21	25.196	-20.402	-1.443	1.00120.83	C
ATOM	1399	C1'	DA	C	21	24.225	-21.328	-0.717	1.00121.16	C
ATOM	1400	N9	DA	C	21	23.422	-22.155	-1.613	1.00120.90	N
ATOM	1401	C8	DA	C	21	23.751	-22.587	-2.868	1.00123.43	C
ATOM	1402	N7	DA	C	21	22.826	-23.325	-3.436	1.00110.08	N
ATOM	1403	C5	DA	C	21	21.822	-23.381	-2.486	1.00106.04	C
ATOM	1404	C6	DA	C	21	20.564	-24.012	-2.476	1.00102.92	C
ATOM	1405	N6	DA	C	21	20.097	-24.737	-3.497	1.00105.40	N
ATOM	1406	N1	DA	C	21	19.807	-23.871	-1.369	1.00 98.22	N
ATOM	1407	C2	DA	C	21	20.282	-23.146	-0.350	1.00101.42	C
ATOM	1408	N3	DA	C	21	21.444	-22.508	-0.242	1.00104.45	N
ATOM	1409	C4	DA	C	21	22.173	-22.667	-1.357	1.00111.09	C
ATOM	1411	O5'	DA	D	1	14.583	-34.861	1.560	1.00119.45	O
ATOM	1412	C5'	DA	D	1	13.982	-33.590	1.798	1.00124.33	C
ATOM	1413	C4'	DA	D	1	14.969	-32.616	2.420	1.00123.83	C
ATOM	1414	O4'	DA	D	1	16.202	-32.561	1.656	1.00121.19	O
ATOM	1415	C3'	DA	D	1	14.522	-31.163	2.445	1.00124.63	C
ATOM	1416	O3'	DA	D	1	13.592	-30.916	3.491	1.00119.40	O
ATOM	1417	C2'	DA	D	1	15.848	-30.442	2.658	1.00119.18	C
ATOM	1418	C1'	DA	D	1	16.833	-31.309	1.879	1.00115.78	C
ATOM	1419	N9	DA	D	1	17.227	-30.721	0.599	1.00110.45	N
ATOM	1420	C8	DA	D	1	16.604	-30.872	-0.610	1.00112.39	C
ATOM	1421	N7	DA	D	1	17.188	-30.224	-1.591	1.00105.28	N
ATOM	1422	C5	DA	D	1	18.268	-29.603	-0.986	1.00 98.83	C
ATOM	1423	C6	DA	D	1	19.283	-28.762	-1.489	1.00 99.24	C
ATOM	1424	N6	DA	D	1	19.365	-28.397	-2.773	1.00103.27	N
ATOM	1425	N1	DA	D	1	20.209	-28.315	-0.616	1.00 85.85	N
ATOM	1426	C2	DA	D	1	20.117	-28.687	0.667	1.00 97.80	C
ATOM	1427	N3	DA	D	1	19.212	-29.468	1.253	1.00102.35	N
ATOM	1428	C4	DA	D	1	18.306	-29.898	0.363	1.00 98.83	C
ATOM	1429	P	DT	D	2	12.515	-29.733	3.347	1.00135.16	P
ATOM	1430	OP1	DT	D	2	12.853	-28.953	2.136	1.00124.60	O
ATOM	1431	OP2	DT	D	2	11.172	-30.343	3.471	1.00141.12	O
ATOM	1432	O5'	DT	D	2	12.766	-28.829	4.643	1.00118.98	O
ATOM	1433	C5'	DT	D	2	13.199	-27.478	4.523	1.00100.82	C
ATOM	1434	C4'	DT	D	2	14.634	-27.317	4.987	1.00100.75	C
ATOM	1435	O4'	DT	D	2	15.542	-27.857	3.995	1.00114.70	O
ATOM	1436	C3'	DT	D	2	15.077	-25.873	5.171	1.00106.09	C
ATOM	1437	O3'	DT	D	2	14.750	-25.413	6.475	1.00119.74	O
ATOM	1438	C2'	DT	D	2	16.585	-25.949	4.948	1.00 99.83	C
ATOM	1439	C1'	DT	D	2	16.750	-27.116	3.978	1.00112.97	C
ATOM	1440	N1	DT	D	2	17.058	-26.684	2.576	1.00109.51	N
ATOM	1441	C2	DT	D	2	18.237	-26.010	2.330	1.00102.77	C
ATOM	1442	O2	DT	D	2	19.050	-25.741	3.195	1.00104.03	O
ATOM	1443	N3	DT	D	2	18.433	-25.656	1.020	1.00 88.97	N
ATOM	1444	C4	DT	D	2	17.588	-25.905	-0.045	1.00 94.94	C
ATOM	1445	O4	DT	D	2	17.854	-25.546	-1.189	1.00 94.51	O
ATOM	1446	C5	DT	D	2	16.375	-26.613	0.280	1.00 91.17	C
ATOM	1447	C7	DT	D	2	15.379	-26.940	-0.794	1.00 93.62	C

ATOM	1448	C6	DT	D	2	16.169	-26.964	1.556	1.00	93.35	C
ATOM	1449	P	DT	D	3	13.882	-24.075	6.670	1.00	118.47	P
ATOM	1450	OP1	DT	D	3	12.766	-24.096	5.698	1.00	92.43	O
ATOM	1451	OP2	DT	D	3	13.595	-23.966	8.118	1.00	105.80	O
ATOM	1452	O5'	DT	D	3	14.890	-22.896	6.275	1.00	99.62	O
ATOM	1453	C5'	DT	D	3	16.024	-22.626	7.094	1.00	106.91	C
ATOM	1454	C4'	DT	D	3	17.105	-21.890	6.322	1.00	107.29	C
ATOM	1455	O4'	DT	D	3	17.437	-22.631	5.123	1.00	110.12	O
ATOM	1456	C3'	DT	D	3	16.723	-20.504	5.821	1.00	118.62	C
ATOM	1457	O3'	DT	D	3	16.972	-19.522	6.824	1.00	129.87	O
ATOM	1458	C2'	DT	D	3	17.625	-20.320	4.603	1.00	115.41	C
ATOM	1459	C1'	DT	D	3	17.924	-21.742	4.135	1.00	105.39	C
ATOM	1460	N1	DT	D	3	17.291	-22.073	2.826	1.00	79.46	N
ATOM	1461	C2	DT	D	3	17.940	-21.703	1.671	1.00	86.43	C
ATOM	1462	O2	DT	D	3	19.008	-21.117	1.661	1.00	96.17	O
ATOM	1463	N3	DT	D	3	17.288	-22.047	0.517	1.00	90.08	N
ATOM	1464	C4	DT	D	3	16.080	-22.709	0.403	1.00	91.61	C
ATOM	1465	O4	DT	D	3	15.575	-22.973	-0.685	1.00	92.68	O
ATOM	1466	C5	DT	D	3	15.455	-23.066	1.651	1.00	86.30	C
ATOM	1467	C7	DT	D	3	14.138	-23.789	1.654	1.00	100.11	C
ATOM	1468	C6	DT	D	3	16.080	-22.737	2.789	1.00	79.30	C
ATOM	1469	P	DG	D	4	15.871	-18.398	7.150	1.00	121.48	P
ATOM	1470	OP1	DG	D	4	16.256	-17.768	8.434	1.00	94.62	O
ATOM	1471	OP2	DG	D	4	14.536	-19.021	7.005	1.00	114.49	O
ATOM	1472	O5'	DG	D	4	16.044	-17.329	5.970	1.00	110.16	O
ATOM	1473	C5'	DG	D	4	17.173	-16.460	5.945	1.00	106.38	C
ATOM	1474	C4'	DG	D	4	17.646	-16.233	4.521	1.00	96.23	C
ATOM	1475	O4'	DG	D	4	17.290	-17.397	3.737	1.00	97.00	O
ATOM	1476	C3'	DG	D	4	17.023	-15.050	3.785	1.00	106.90	C
ATOM	1477	O3'	DG	D	4	17.892	-13.920	3.830	1.00	111.71	O
ATOM	1478	C2'	DG	D	4	16.821	-15.540	2.352	1.00	96.03	C
ATOM	1479	C1'	DG	D	4	17.181	-17.022	2.381	1.00	105.28	C
ATOM	1480	N9	DG	D	4	16.192	-17.877	1.731	1.00	110.09	N
ATOM	1481	C8	DG	D	4	15.181	-18.590	2.331	1.00	112.31	C
ATOM	1482	N7	DG	D	4	14.454	-19.267	1.485	1.00	100.89	N
ATOM	1483	C5	DG	D	4	15.019	-18.982	0.249	1.00	87.86	C
ATOM	1484	C6	DG	D	4	14.652	-19.433	-1.040	1.00	100.39	C
ATOM	1485	O6	DG	D	4	13.725	-20.196	-1.347	1.00	113.93	O
ATOM	1486	N1	DG	D	4	15.482	-18.906	-2.024	1.00	102.27	N
ATOM	1487	C2	DG	D	4	16.534	-18.053	-1.787	1.00	100.78	C
ATOM	1488	N2	DG	D	4	17.224	-17.650	-2.862	1.00	105.32	N
ATOM	1489	N3	DG	D	4	16.890	-17.624	-0.584	1.00	97.03	N
ATOM	1490	C4	DG	D	4	16.088	-18.128	0.382	1.00	94.94	C
ATOM	1491	P	DT	D	5	17.419	-12.509	3.224	1.00	108.78	P
ATOM	1492	OP1	DT	D	5	18.417	-11.496	3.631	1.00	98.75	O
ATOM	1493	OP2	DT	D	5	15.991	-12.324	3.568	1.00	97.36	O
ATOM	1494	O5'	DT	D	5	17.520	-12.709	1.639	1.00	93.26	O
ATOM	1495	C5'	DT	D	5	18.794	-12.863	1.030	1.00	95.92	C
ATOM	1496	C4'	DT	D	5	18.657	-13.144	-0.455	1.00	104.51	C
ATOM	1497	O4'	DT	D	5	17.910	-14.368	-0.660	1.00	105.83	O
ATOM	1498	C3'	DT	D	5	17.878	-12.104	-1.243	1.00	95.63	C
ATOM	1499	O3'	DT	D	5	18.713	-11.035	-1.655	1.00	105.61	O
ATOM	1500	C2'	DT	D	5	17.368	-12.896	-2.440	1.00	94.91	C
ATOM	1501	C1'	DT	D	5	17.280	-14.329	-1.928	1.00	104.96	C
ATOM	1502	N1	DT	D	5	15.875	-14.822	-1.806	1.00	108.15	N
ATOM	1503	C2	DT	D	5	15.232	-15.301	-2.928	1.00	108.73	C
ATOM	1504	O2	DT	D	5	15.748	-15.347	-4.031	1.00	103.45	O
ATOM	1505	N3	DT	D	5	13.949	-15.732	-2.710	1.00	100.68	N

```
ATOM   1506  C4   DT D   5      13.259 -15.732  -1.514  1.00 96.66           C
ATOM   1507  O4   DT D   5      12.106 -16.142  -1.425  1.00 94.24           O
ATOM   1508  C5   DT D   5      13.990 -15.218  -0.384  1.00 92.10           C
ATOM   1509  C7   DT D   5      13.347 -15.165   0.967  1.00 87.50           C
ATOM   1510  C6   DT D   5      15.244 -14.791  -0.579  1.00102.10           C
ATOM   1511  P    DT D   6      18.153  -9.936  -2.683  1.00121.02           P
ATOM   1512  OP1  DT D   6      19.072  -8.776  -2.650  1.00106.01           O
ATOM   1513  OP2  DT D   6      16.716  -9.748  -2.384  1.00129.71           O
ATOM   1514  O5'  DT D   6      18.255 -10.652  -4.110  1.00 88.33           O
ATOM   1515  C5'  DT D   6      17.668 -10.035  -5.248  1.00110.27           C
ATOM   1516  C4'  DT D   6      17.338 -11.068  -6.309  1.00112.34           C
ATOM   1517  O4'  DT D   6      16.752 -12.229  -5.669  1.00119.00           O
ATOM   1518  C3'  DT D   6      16.348 -10.589  -7.363  1.00108.63           C
ATOM   1519  O3'  DT D   6      17.032 -10.311  -8.582  1.00121.56           O
ATOM   1520  C2'  DT D   6      15.344 -11.729  -7.523  1.00 99.88           C
ATOM   1521  C1'  DT D   6      15.595 -12.673  -6.352  1.00113.50           C
ATOM   1522  N1   DT D   6      14.463 -12.754  -5.369  1.00113.72           N
ATOM   1523  C2   DT D   6      13.368 -13.539  -5.664  1.00112.06           C
ATOM   1524  O2   DT D   6      13.260 -14.175  -6.697  1.00112.20           O
ATOM   1525  N3   DT D   6      12.392 -13.550  -4.701  1.00107.62           N
ATOM   1526  C4   DT D   6      12.400 -12.873  -3.494  1.00107.76           C
ATOM   1527  O4   DT D   6      11.474 -12.947  -2.692  1.00 93.84           O
ATOM   1528  C5   DT D   6      13.574 -12.074  -3.247  1.00109.21           C
ATOM   1529  C7   DT D   6      13.702 -11.295  -1.970  1.00103.26           C
ATOM   1530  C6   DT D   6      14.535 -12.054  -4.181  1.00107.03           C
ATOM   1531  P    DC D   7      16.508  -9.143  -9.553  1.00136.27           P
ATOM   1532  OP1  DC D   7      17.080  -7.866  -9.070  1.00108.43           O
ATOM   1533  OP2  DC D   7      16.741  -9.575 -10.950  1.00113.88           O
ATOM   1534  O5'  DC D   7      14.930  -9.125  -9.306  1.00128.36           O
ATOM   1535  C5'  DC D   7      14.037  -8.940 -10.396  1.00126.81           C
ATOM   1536  C4'  DC D   7      13.621 -10.274 -10.990  1.00121.30           C
ATOM   1537  O4'  DC D   7      13.643 -11.295  -9.959  1.00121.96           O
ATOM   1538  C3'  DC D   7      12.217 -10.277 -11.582  1.00121.69           C
ATOM   1539  O3'  DC D   7      12.288 -10.134 -12.998  1.00123.64           O
ATOM   1540  C2'  DC D   7      11.623 -11.617 -11.152  1.00105.53           C
ATOM   1541  C1'  DC D   7      12.407 -11.981  -9.894  1.00108.59           C
ATOM   1542  N1   DC D   7      11.738 -11.629  -8.599  1.00 97.71           N
ATOM   1543  C2   DC D   7      10.713 -12.443  -8.105  1.00101.66           C
ATOM   1544  O2   DC D   7      10.364 -13.436  -8.754  1.00100.41           O
ATOM   1545  N3   DC D   7      10.123 -12.119  -6.927  1.00 99.51           N
ATOM   1546  C4   DC D   7      10.524 -11.041  -6.253  1.00107.85           C
ATOM   1547  N4   DC D   7       9.914 -10.757  -5.098  1.00108.33           N
ATOM   1548  C5   DC D   7      11.568 -10.201  -6.736  1.00 98.71           C
ATOM   1549  C6   DC D   7      12.139 -10.529  -7.898  1.00 95.90           C
ATOM   1550  P    DT D   8      11.317  -9.105 -13.792  1.00123.67           P
ATOM   1551  OP1  DT D   8      11.788  -9.027 -15.198  1.00134.64           O
ATOM   1552  OP2  DT D   8      11.183  -7.852 -12.997  1.00111.50           O
ATOM   1553  O5'  DT D   8       9.912  -9.858 -13.809  1.00 92.36           O
ATOM   1554  C5'  DT D   8       9.771 -11.145 -14.440  1.00115.03           C
ATOM   1555  C4'  DT D   8       8.538 -11.859 -13.935  1.00117.15           C
ATOM   1556  O4'  DT D   8       8.567 -11.925 -12.488  1.00120.77           O
ATOM   1557  C3'  DT D   8       7.210 -11.193 -14.305  1.00116.28           C
ATOM   1558  O3'  DT D   8       6.270 -12.189 -14.716  1.00132.16           O
ATOM   1559  C2'  DT D   8       6.753 -10.545 -13.012  1.00116.85           C
ATOM   1560  C1'  DT D   8       7.332 -11.472 -11.962  1.00118.79           C
ATOM   1561  N1   DT D   8       7.602 -10.822 -10.669  1.00119.15           N
ATOM   1562  C2   DT D   8       6.999 -11.339  -9.546  1.00111.87           C
ATOM   1563  O2   DT D   8       6.257 -12.306  -9.574  1.00111.67           O
```

ATOM	1564	N3	DT	D	8	7.293	-10.672	-8.382	1.00105.83	N
ATOM	1565	C4	DT	D	8	8.112	-9.569	-8.235	1.00103.46	C
ATOM	1566	O4	DT	D	8	8.279	-9.076	-7.123	1.00100.98	O
ATOM	1567	C5	DT	D	8	8.713	-9.081	-9.455	1.00 95.54	C
ATOM	1568	C7	DT	D	8	9.614	-7.889	-9.395	1.00 94.56	C
ATOM	1569	C6	DT	D	8	8.428	-9.721	-10.596	1.00108.08	C
ATOM	1570	P	DC	D	9	5.530	-12.063	-16.128	1.00150.26	P
ATOM	1571	OP1	DC	D	9	5.741	-13.331	-16.870	1.00126.43	O
ATOM	1572	OP2	DC	D	9	5.931	-10.773	-16.748	1.00115.18	O
ATOM	1573	O5'	DC	D	9	3.994	-11.981	-15.717	1.00155.34	O
ATOM	1574	C5'	DC	D	9	3.320	-10.713	-15.618	1.00126.37	C
ATOM	1575	C4'	DC	D	9	2.351	-10.724	-14.459	1.00111.64	C
ATOM	1576	O4'	DC	D	9	3.072	-10.560	-13.216	1.00130.54	O
ATOM	1577	C3'	DC	D	9	1.302	-9.609	-14.504	1.00110.98	C
ATOM	1578	O3'	DC	D	9	-0.011	-10.159	-14.357	1.00108.04	O
ATOM	1579	C2'	DC	D	9	1.632	-8.734	-13.308	1.00123.79	C
ATOM	1580	C1'	DC	D	9	2.355	-9.680	-12.373	1.00129.75	C
ATOM	1581	N1	DC	D	9	3.321	-9.015	-11.487	1.00117.49	N
ATOM	1582	C2	DC	D	9	3.800	-9.702	-10.368	1.00116.12	C
ATOM	1583	O2	DC	D	9	3.402	-10.858	-10.159	1.00121.31	O
ATOM	1584	N3	DC	D	9	4.683	-9.091	-9.545	1.00104.84	N
ATOM	1585	C4	DC	D	9	5.085	-7.845	-9.805	1.00104.62	C
ATOM	1586	N4	DC	D	9	5.955	-7.281	-8.966	1.00 95.15	N
ATOM	1587	C5	DC	D	9	4.609	-7.120	-10.935	1.00111.84	C
ATOM	1588	C6	DC	D	9	3.734	-7.736	-11.740	1.00111.58	C
ATOM	1589	P	DT	D	10	-1.244	-9.516	-15.151	1.00116.98	P
ATOM	1590	OP1	DT	D	10	-1.432	-10.284	-16.407	1.00131.40	O
ATOM	1591	OP2	DT	D	10	-1.042	-8.046	-15.211	1.00106.42	O
ATOM	1592	O5'	DT	D	10	-2.480	-9.811	-14.190	1.00125.89	O
ATOM	1593	C5'	DT	D	10	-2.824	-11.160	-13.825	1.00114.51	C
ATOM	1594	C4'	DT	D	10	-2.894	-11.299	-12.322	1.00110.37	C
ATOM	1595	O4'	DT	D	10	-1.608	-10.985	-11.744	1.00 98.05	O
ATOM	1596	C3'	DT	D	10	-3.870	-10.350	-11.632	1.00106.74	C
ATOM	1597	O3'	DT	D	10	-5.226	-10.838	-11.735	1.00110.72	O
ATOM	1598	C2'	DT	D	10	-3.288	-10.253	-10.231	1.00115.83	C
ATOM	1599	C1'	DT	D	10	-1.787	-10.424	-10.446	1.00108.52	C
ATOM	1600	N1	DT	D	10	-1.027	-9.162	-10.381	1.00103.57	N
ATOM	1601	C2	DT	D	10	-0.101	-9.014	-9.375	1.00113.01	C
ATOM	1602	O2	DT	D	10	0.129	-9.881	-8.548	1.00128.15	O
ATOM	1603	N3	DT	D	10	0.557	-7.810	-9.377	1.00105.50	N
ATOM	1604	C4	DT	D	10	0.379	-6.762	-10.260	1.00 96.60	C
ATOM	1605	O4	DT	D	10	1.047	-5.740	-10.139	1.00 97.78	O
ATOM	1606	C5	DT	D	10	-0.615	-6.982	-11.284	1.00102.24	C
ATOM	1607	C7	DT	D	10	-0.876	-5.904	-12.288	1.00 93.64	C
ATOM	1608	C6	DT	D	10	-1.262	-8.154	-11.291	1.00104.66	C
ATOM	1609	P	DA	D	11	-5.955	-11.718	-10.574	1.00121.95	P
ATOM	1610	OP1	DA	D	11	-5.054	-12.821	-10.173	1.00120.00	O
ATOM	1611	OP2	DA	D	11	-7.316	-12.025	-11.068	1.00138.08	O
ATOM	1612	O5'	DA	D	11	-6.107	-10.696	-9.352	1.00124.93	O
ATOM	1613	C5'	DA	D	11	-6.815	-11.091	-8.182	1.00125.06	C
ATOM	1614	C4'	DA	D	11	-6.081	-10.648	-6.928	1.00107.98	C
ATOM	1615	O4'	DA	D	11	-4.753	-10.187	-7.290	1.00114.33	O
ATOM	1616	C3'	DA	D	11	-6.724	-9.493	-6.174	1.00101.69	C
ATOM	1617	O3'	DA	D	11	-7.562	-9.983	-5.144	1.00102.08	O
ATOM	1618	C2'	DA	D	11	-5.534	-8.729	-5.607	1.00105.77	C
ATOM	1619	C1'	DA	D	11	-4.454	-8.960	-6.654	1.00 99.26	C
ATOM	1620	N9	DA	D	11	-4.380	-7.918	-7.673	1.00 96.32	N
ATOM	1621	C8	DA	D	11	-5.173	-7.776	-8.777	1.00100.24	C

ATOM	1622	N7	DA	D	11	-4.864	-6.741	-9.521	1.00	98.79	N
ATOM	1623	C5	DA	D	11	-3.794	-6.165	-8.860	1.00	89.52	C
ATOM	1624	C6	DA	D	11	-3.006	-5.030	-9.135	1.00	89.41	C
ATOM	1625	N6	DA	D	11	-3.191	-4.248	-10.201	1.00	91.25	N
ATOM	1626	N1	DA	D	11	-2.016	-4.730	-8.269	1.00	98.74	N
ATOM	1627	C2	DA	D	11	-1.837	-5.519	-7.204	1.00	103.89	C
ATOM	1628	N3	DA	D	11	-2.509	-6.608	-6.839	1.00	93.75	N
ATOM	1629	C4	DA	D	11	-3.484	-6.878	-7.719	1.00	94.37	C
ATOM	1630	P	DA	D	12	-8.753	-9.068	-4.577	1.00	110.30	P
ATOM	1631	OP1	DA	D	12	-9.449	-9.841	-3.525	1.00	95.08	O
ATOM	1632	OP2	DA	D	12	-9.498	-8.543	-5.743	1.00	107.11	O
ATOM	1633	O5'	DA	D	12	-7.996	-7.836	-3.897	1.00	92.75	O
ATOM	1634	C5'	DA	D	12	-7.099	-8.079	-2.823	1.00	97.92	C
ATOM	1635	C4'	DA	D	12	-6.375	-6.805	-2.437	1.00	90.46	C
ATOM	1636	O4'	DA	D	12	-5.700	-6.255	-3.600	1.00	81.25	O
ATOM	1637	C3'	DA	D	12	-7.289	-5.700	-1.919	1.00	89.89	C
ATOM	1638	O3'	DA	D	12	-6.692	-5.059	-0.806	1.00	70.86	O
ATOM	1639	C2'	DA	D	12	-7.418	-4.767	-3.115	1.00	79.78	C
ATOM	1640	C1'	DA	D	12	-6.027	-4.885	-3.714	1.00	76.96	C
ATOM	1641	N9	DA	D	12	-5.955	-4.457	-5.106	1.00	90.10	N
ATOM	1642	C8	DA	D	12	-6.752	-4.848	-6.146	1.00	96.98	C
ATOM	1643	N7	DA	D	12	-6.443	-4.280	-7.290	1.00	83.13	N
ATOM	1644	C5	DA	D	12	-5.371	-3.462	-6.974	1.00	94.94	C
ATOM	1645	C6	DA	D	12	-4.579	-2.587	-7.745	1.00	100.82	C
ATOM	1646	N6	DA	D	12	-4.763	-2.390	-9.055	1.00	95.85	N
ATOM	1647	N1	DA	D	12	-3.586	-1.922	-7.114	1.00	104.93	N
ATOM	1648	C2	DA	D	12	-3.405	-2.122	-5.802	1.00	93.75	C
ATOM	1649	N3	DA	D	12	-4.082	-2.914	-4.974	1.00	86.56	N
ATOM	1650	C4	DA	D	12	-5.059	-3.559	-5.631	1.00	92.97	C
ATOM	1651	P	DA	D	13	-7.570	-4.249	0.284	1.00	87.09	P
ATOM	1652	OP1	DA	D	13	-7.974	-5.196	1.354	1.00	86.09	O
ATOM	1653	OP2	DA	D	13	-8.603	-3.436	-0.419	1.00	66.23	O
ATOM	1654	O5'	DA	D	13	-6.511	-3.220	0.879	1.00	74.25	O
ATOM	1655	C5'	DA	D	13	-5.197	-3.095	0.304	1.00	72.42	C
ATOM	1656	C4'	DA	D	13	-5.007	-1.720	-0.294	1.00	58.91	C
ATOM	1657	O4'	DA	D	13	-5.245	-1.752	-1.716	1.00	68.03	O
ATOM	1658	C3'	DA	D	13	-5.935	-0.638	0.270	1.00	54.34	C
ATOM	1659	O3'	DA	D	13	-5.119	0.159	1.147	1.00	49.39	O
ATOM	1660	C2'	DA	D	13	-6.514	0.051	-0.963	1.00	71.10	C
ATOM	1661	C1'	DA	D	13	-5.674	-0.467	-2.115	1.00	73.29	C
ATOM	1662	N9	DA	D	13	-6.384	-0.612	-3.383	1.00	77.03	N
ATOM	1663	C8	DA	D	13	-7.525	-1.329	-3.644	1.00	81.38	C
ATOM	1664	N7	DA	D	13	-7.896	-1.293	-4.901	1.00	88.48	N
ATOM	1665	C5	DA	D	13	-6.932	-0.501	-5.509	1.00	82.17	C
ATOM	1666	C6	DA	D	13	-6.755	-0.079	-6.838	1.00	81.46	C
ATOM	1667	N6	DA	D	13	-7.586	-0.398	-7.833	1.00	86.82	N
ATOM	1668	N1	DA	D	13	-5.690	0.706	-7.110	1.00	83.49	N
ATOM	1669	C2	DA	D	13	-4.865	1.036	-6.110	1.00	81.57	C
ATOM	1670	N3	DA	D	13	-4.923	0.700	-4.824	1.00	69.38	N
ATOM	1671	C4	DA	D	13	-5.994	-0.077	-4.586	1.00	73.26	C
ATOM	1672	P	DG	D	14	-5.191	1.760	1.159	1.00	63.31	P
ATOM	1673	OP1	DG	D	14	-4.303	2.243	2.244	1.00	66.34	O
ATOM	1674	OP2	DG	D	14	-6.618	2.167	1.162	1.00	63.45	O
ATOM	1675	O5'	DG	D	14	-4.491	2.158	-0.215	1.00	74.27	O
ATOM	1676	C5'	DG	D	14	-3.584	3.269	-0.301	1.00	61.77	C
ATOM	1677	C4'	DG	D	14	-4.231	4.404	-1.059	1.00	65.42	C
ATOM	1678	O4'	DG	D	14	-4.835	3.906	-2.279	1.00	65.37	O
ATOM	1679	C3'	DG	D	14	-5.347	5.129	-0.297	1.00	65.65	C

ATOM	1680	O3'	DG	D	14	-5.232	6.542	-0.461	1.00	60.38	O
ATOM	1681	C2'	DG	D	14	-6.607	4.728	-1.038	1.00	67.89	C
ATOM	1682	C1'	DG	D	14	-6.060	4.588	-2.439	1.00	65.96	C
ATOM	1683	N9	DG	D	14	-6.894	3.837	-3.370	1.00	71.76	N
ATOM	1684	C8	DG	D	14	-7.945	3.004	-3.071	1.00	72.88	C
ATOM	1685	N7	DG	D	14	-8.541	2.531	-4.131	1.00	81.75	N
ATOM	1686	C5	DG	D	14	-7.859	3.107	-5.194	1.00	69.47	C
ATOM	1687	C6	DG	D	14	-8.054	2.973	-6.593	1.00	68.57	C
ATOM	1688	O6	DG	D	14	-8.888	2.285	-7.193	1.00	80.48	O
ATOM	1689	N1	DG	D	14	-7.134	3.731	-7.311	1.00	66.80	N
ATOM	1690	C2	DG	D	14	-6.156	4.519	-6.757	1.00	72.43	C
ATOM	1691	N2	DG	D	14	-5.367	5.178	-7.617	1.00	74.77	N
ATOM	1692	N3	DG	D	14	-5.968	4.656	-5.456	1.00	81.35	N
ATOM	1693	C4	DG	D	14	-6.848	3.926	-4.739	1.00	77.85	C
ATOM	1694	P	DA	D	15	-4.487	7.437	0.630	1.00	76.38	P
ATOM	1695	OP1	DA	D	15	-3.273	6.711	1.082	1.00	77.11	O
ATOM	1696	OP2	DA	D	15	-5.492	7.878	1.630	1.00	73.31	O
ATOM	1697	O5'	DA	D	15	-4.029	8.697	-0.230	1.00	85.60	O
ATOM	1698	C5'	DA	D	15	-3.171	8.518	-1.373	1.00	73.95	C
ATOM	1699	C4'	DA	D	15	-3.811	9.066	-2.630	1.00	66.77	C
ATOM	1700	O4'	DA	D	15	-4.770	8.133	-3.174	1.00	79.35	O
ATOM	1701	C3'	DA	D	15	-4.558	10.393	-2.465	1.00	98.42	C
ATOM	1702	O3'	DA	D	15	-3.798	11.393	-3.158	1.00119.96		O
ATOM	1703	C2'	DA	D	15	-5.912	10.156	-3.120	1.00102.38		C
ATOM	1704	C1'	DA	D	15	-5.705	8.884	-3.921	1.00	85.48	C
ATOM	1705	N9	DA	D	15	-6.897	8.058	-4.107	1.00	78.49	N
ATOM	1706	C8	DA	D	15	-7.680	7.473	-3.144	1.00	78.45	C
ATOM	1707	N7	DA	D	15	-8.675	6.769	-3.627	1.00	79.75	N
ATOM	1708	C5	DA	D	15	-8.539	6.902	-5.002	1.00	69.93	C
ATOM	1709	C6	DA	D	15	-9.284	6.391	-6.080	1.00	69.00	C
ATOM	1710	N6	DA	D	15	-10.364	5.620	-5.934	1.00	62.02	N
ATOM	1711	N1	DA	D	15	-8.884	6.715	-7.329	1.00	72.01	N
ATOM	1712	C2	DA	D	15	-7.806	7.495	-7.473	1.00	78.74	C
ATOM	1713	N3	DA	D	15	-7.020	8.029	-6.541	1.00	66.61	N
ATOM	1714	C4	DA	D	15	-7.448	7.692	-5.312	1.00	64.79	C
ATOM	1715	P	DG	D	16	-4.146	12.975	-3.054	1.00127.15		P
ATOM	1716	OP1	DG	D	16	-2.928	13.709	-3.468	1.00105.14		O
ATOM	1717	OP2	DG	D	16	-4.754	13.241	-1.731	1.00101.40		O
ATOM	1718	O5'	DG	D	16	-5.277	13.179	-4.167	1.00103.95		O
ATOM	1719	C5'	DG	D	16	-4.962	12.937	-5.532	1.00106.12		C
ATOM	1720	C4'	DG	D	16	-6.196	13.033	-6.411	1.00112.33		C
ATOM	1721	O4'	DG	D	16	-7.042	11.870	-6.203	1.00107.16		O
ATOM	1722	C3'	DG	D	16	-7.083	14.252	-6.151	1.00117.08		C
ATOM	1723	O3'	DG	D	16	-7.444	14.877	-7.388	1.00116.29		O
ATOM	1724	C2'	DG	D	16	-8.283	13.642	-5.435	1.00107.50		C
ATOM	1725	C1'	DG	D	16	-8.380	12.317	-6.177	1.00104.88		C
ATOM	1726	N9	DG	D	16	-9.237	11.305	-5.565	1.00	88.24	N
ATOM	1727	C8	DG	D	16	-9.393	11.021	-4.230	1.00	80.62	C
ATOM	1728	N7	DG	D	16	-10.238	10.051	-4.003	1.00	67.60	N
ATOM	1729	C5	DG	D	16	-10.664	9.665	-5.269	1.00	74.01	C
ATOM	1730	C6	DG	D	16	-11.581	8.659	-5.664	1.00	91.41	C
ATOM	1731	O6	DG	D	16	-12.222	7.879	-4.943	1.00	87.49	O
ATOM	1732	N1	DG	D	16	-11.727	8.603	-7.050	1.00	86.31	N
ATOM	1733	C2	DG	D	16	-11.069	9.417	-7.942	1.00	80.47	C
ATOM	1734	N2	DG	D	16	-11.336	9.214	-9.240	1.00	94.06	N
ATOM	1735	N3	DG	D	16	-10.208	10.362	-7.585	1.00	78.49	N
ATOM	1736	C4	DG	D	16	-10.058	10.429	-6.240	1.00	78.17	C
ATOM	1737	P	DA	D	17	-8.075	16.356	-7.410	1.00122.79		P

ATOM	1738	OP1	DA	D	17	-8.049	16.822	-8.813	1.00	120.25	O
ATOM	1739	OP2	DA	D	17	-7.412	17.160	-6.356	1.00	94.57	O
ATOM	1740	O5'	DA	D	17	-9.601	16.133	-6.982	1.00	125.68	O
ATOM	1741	C5'	DA	D	17	-10.671	16.486	-7.862	1.00	117.33	C
ATOM	1742	C4'	DA	D	17	-10.783	15.539	-9.048	1.00	116.52	C
ATOM	1743	O4'	DA	D	17	-10.453	14.190	-8.625	1.00	113.60	O
ATOM	1744	C3'	DA	D	17	-12.176	15.481	-9.676	1.00	127.56	C
ATOM	1745	O3'	DA	D	17	-12.175	16.077	-10.975	1.00	135.81	O
ATOM	1746	C2'	DA	D	17	-12.523	13.996	-9.746	1.00	113.50	C
ATOM	1747	C1'	DA	D	17	-11.560	13.322	-8.777	1.00	108.07	C
ATOM	1748	N9	DA	D	17	-12.131	13.043	-7.459	1.00	96.77	N
ATOM	1749	C8	DA	D	17	-11.746	13.586	-6.266	1.00	95.84	C
ATOM	1750	N7	DA	D	17	-12.431	13.151	-5.235	1.00	89.17	N
ATOM	1751	C5	DA	D	17	-13.331	12.258	-5.790	1.00	78.89	C
ATOM	1752	C6	DA	D	17	-14.339	11.458	-5.221	1.00	84.24	C
ATOM	1753	N6	DA	D	17	-14.610	11.441	-3.911	1.00	88.05	N
ATOM	1754	N1	DA	D	17	-15.060	10.677	-6.051	1.00	91.89	N
ATOM	1755	C2	DA	D	17	-14.783	10.702	-7.360	1.00	85.23	C
ATOM	1756	N3	DA	D	17	-13.861	11.411	-8.011	1.00	83.99	N
ATOM	1757	C4	DA	D	17	-13.161	12.178	-7.160	1.00	84.34	C
ATOM	1758	P	DA	D	18	-13.536	16.163	-11.827	1.00	131.98	P
ATOM	1759	OP1	DA	D	18	-13.242	16.887	-13.084	1.00	140.31	O
ATOM	1760	OP2	DA	D	18	-14.594	16.662	-10.922	1.00	96.93	O
ATOM	1761	O5'	DA	D	18	-13.864	14.638	-12.187	1.00	113.55	O
ATOM	1762	C5'	DA	D	18	-14.942	14.322	-13.054	1.00	110.78	C
ATOM	1763	C4'	DA	D	18	-16.092	13.677	-12.297	1.00	123.64	C
ATOM	1764	O4'	DA	D	18	-15.730	13.475	-10.898	1.00	106.97	O
ATOM	1765	C3'	DA	D	18	-17.386	14.490	-12.271	1.00	129.49	C
ATOM	1766	O3'	DA	D	18	-18.511	13.636	-12.471	1.00	133.18	O
ATOM	1767	C2'	DA	D	18	-17.348	15.078	-10.866	1.00	120.73	C
ATOM	1768	C1'	DA	D	18	-16.846	13.856	-10.119	1.00	116.47	C
ATOM	1769	N9	DA	D	18	-16.499	14.101	-8.720	1.00	113.76	N
ATOM	1770	C8	DA	D	18	-15.734	15.117	-8.216	1.00	112.51	C
ATOM	1771	N7	DA	D	18	-15.601	15.084	-6.909	1.00	95.13	N
ATOM	1772	C5	DA	D	18	-16.334	13.973	-6.528	1.00	90.99	C
ATOM	1773	C6	DA	D	18	-16.591	13.392	-5.270	1.00	89.19	C
ATOM	1774	N6	DA	D	18	-16.108	13.885	-4.125	1.00	87.44	N
ATOM	1775	N1	DA	D	18	-17.361	12.284	-5.235	1.00	76.40	N
ATOM	1776	C2	DA	D	18	-17.840	11.800	-6.387	1.00	89.55	C
ATOM	1777	N3	DA	D	18	-17.668	12.258	-7.627	1.00	97.17	N
ATOM	1778	C4	DA	D	18	-16.897	13.357	-7.630	1.00	97.94	C
ATOM	1779	P	DC	D	19	-19.582	13.954	-13.626	1.00	144.10	P
ATOM	1780	OP1	DC	D	19	-20.307	12.699	-13.924	1.00	124.65	O
ATOM	1781	OP2	DC	D	19	-18.871	14.655	-14.717	1.00	133.58	O
ATOM	1782	O5'	DC	D	19	-20.592	14.993	-12.944	1.00	142.12	O
ATOM	1783	C5'	DC	D	19	-20.808	14.991	-11.531	1.00	120.59	C
ATOM	1784	C4'	DC	D	19	-21.624	13.802	-11.058	1.00	132.26	C
ATOM	1785	O4'	DC	D	19	-21.016	13.268	-9.850	1.00	123.82	O
ATOM	1786	C3'	DC	D	19	-23.058	14.123	-10.663	1.00	135.61	C
ATOM	1787	O3'	DC	D	19	-23.898	12.977	-10.781	1.00	148.37	O
ATOM	1788	C2'	DC	D	19	-22.868	14.570	-9.218	1.00	118.33	C
ATOM	1789	C1'	DC	D	19	-21.818	13.579	-8.725	1.00	116.66	C
ATOM	1790	N1	DC	D	19	-20.951	14.125	-7.643	1.00	108.92	N
ATOM	1791	C2	DC	D	19	-20.930	13.522	-6.378	1.00	98.15	C
ATOM	1792	O2	DC	D	19	-21.635	12.527	-6.159	1.00	89.54	O
ATOM	1793	N3	DC	D	19	-20.131	14.048	-5.416	1.00	83.70	N
ATOM	1794	C4	DC	D	19	-19.383	15.121	-5.679	1.00	83.71	C
ATOM	1795	N4	DC	D	19	-18.611	15.602	-4.699	1.00	82.25	N

```
ATOM   1796  C5   DC D  19     -19.393  15.747  -6.959  1.00 97.53           C
ATOM   1797  C6   DC D  19     -20.183  15.223  -7.900  1.00104.30           C
ATOM   1798  P    DT D  20     -25.409  13.027 -10.237  1.00145.15           P
ATOM   1799  OP1  DT D  20     -26.161  11.909 -10.849  1.00120.91           O
ATOM   1800  OP2  DT D  20     -25.887  14.418 -10.405  1.00150.14           O
ATOM   1801  O5'  DT D  20     -25.259  12.749  -8.670  1.00119.47           O
ATOM   1802  C5'  DT D  20     -26.292  13.129  -7.774  1.00116.41           C
ATOM   1803  C4'  DT D  20     -26.018  12.593  -6.383  1.00113.86           C
ATOM   1804  O4'  DT D  20     -24.740  13.097  -5.914  1.00 97.29           O
ATOM   1805  C3'  DT D  20     -27.054  12.989  -5.337  1.00125.65           C
ATOM   1806  O3'  DT D  20     -27.377  11.859  -4.534  1.00132.02           O
ATOM   1807  C2'  DT D  20     -26.361  14.092  -4.543  1.00115.56           C
ATOM   1808  C1'  DT D  20     -24.894  13.697  -4.644  1.00 94.19           C
ATOM   1809  N1   DT D  20     -23.943  14.842  -4.540  1.00 93.35           N
ATOM   1810  C2   DT D  20     -23.312  15.079  -3.340  1.00 91.88           C
ATOM   1811  O2   DT D  20     -23.493  14.400  -2.344  1.00 88.36           O
ATOM   1812  N3   DT D  20     -22.456  16.150  -3.347  1.00 81.53           N
ATOM   1813  C4   DT D  20     -22.173  16.989  -4.407  1.00 91.50           C
ATOM   1814  O4   DT D  20     -21.384  17.924  -4.299  1.00 96.27           O
ATOM   1815  C5   DT D  20     -22.867  16.683  -5.634  1.00 91.19           C
ATOM   1816  C7   DT D  20     -22.639  17.526  -6.855  1.00 99.54           C
ATOM   1817  C6   DT D  20     -23.707  15.638  -5.644  1.00 87.87           C
ATOM   1818  P    DT D  21     -28.895  11.340  -4.428  1.00170.56           P
ATOM   1819  OP1  DT D  21     -29.642  11.844  -5.603  1.00144.36           O
ATOM   1820  OP2  DT D  21     -28.848   9.887  -4.152  1.00161.33           O
ATOM   1821  O5'  DT D  21     -29.460  12.072  -3.125  1.00146.52           O
ATOM   1822  C5'  DT D  21     -28.646  12.234  -1.970  1.00131.42           C
ATOM   1823  C4'  DT D  21     -29.201  13.338  -1.088  1.00130.57           C
ATOM   1824  O4'  DT D  21     -28.148  14.307  -0.829  1.00119.15           O
ATOM   1825  C3'  DT D  21     -30.355  14.130  -1.700  1.00130.77           C
ATOM   1826  O3'  DT D  21     -31.290  14.524  -0.705  1.00118.68           O
ATOM   1827  C2'  DT D  21     -29.627  15.322  -2.310  1.00126.12           C
ATOM   1828  C1'  DT D  21     -28.586  15.592  -1.230  1.00123.41           C
ATOM   1829  N1   DT D  21     -27.407  16.430  -1.641  1.00106.25           N
ATOM   1830  C2   DT D  21     -26.488  16.776  -0.675  1.00102.16           C
ATOM   1831  O2   DT D  21     -26.584  16.428   0.489  1.00114.63           O
ATOM   1832  N3   DT D  21     -25.444  17.546  -1.117  1.00 96.19           N
ATOM   1833  C4   DT D  21     -25.229  18.000  -2.404  1.00 97.12           C
ATOM   1834  O4   DT D  21     -24.257  18.687  -2.703  1.00 97.77           O
ATOM   1835  C5   DT D  21     -26.227  17.607  -3.368  1.00 92.58           C
ATOM   1836  C7   DT D  21     -26.102  18.039  -4.798  1.00 96.52           C
ATOM   1837  C6   DT D  21     -27.255  16.854  -2.949  1.00101.71           C
END
```
